# A qualitative exploration of a family self-help mental health program in El Salvador

**DOI:** 10.1186/s13033-016-0058-6

**Published:** 2016-04-01

**Authors:** Samuel V. Nickels, Nelson A. Flamenco Arvaiza, Myrna S. Rojas Valle

**Affiliations:** James Madison University, 800 S Main St, Harrisonburg, VA 22807 USA; The Center for Health and Human Development, 340 Maryland Ave, Harrisonburg, VA 22801 USA; Association for Training and Research in Mental Health, ACISAM, Residencial Montebello, Avenida Monte Urales, Casa #10F, Mexicanos, San Salvador, El Salvador

**Keywords:** Global mental health, El Salvador, Family program, Self-help group, Community based rehabilitation, Leadership, Empowerment, Social capital

## Abstract

**Background:**

There is a significant gap in our knowledge regarding community-based self-help groups and their benefits for persons living with mental conditions and their family caregivers in low and middle income countries. This study describes a such a program in El Salvador and explores participants’ perceptions of program effectiveness and benefits.

**Case description:**

The Family Education, Support and Empowerment Program is a multi-component program in the capital that is facilitated by nonprofit professionals but carried out primarily by volunteers. A focus group methodology to build evaluation and research capacity in the organization was used. The study consisted of a questionnaire completed by participants individually, followed by two focus group sessions with the same ten people.

**Results:**

The study found perceptions of multiple benefits across social, functional, and economic dimensions and a variety of achievements at organizational and national levels.

**Discussion:**

This study identified a family self-help program in El Salvador as a potentially highly beneficial program for its participants. This appears to be the first study to explore benefits across micro, mezzo and macro social levels and to include discussion of more diverse potential benefits such as individual and organizational social capital, leadership, and advocacy. These factors should be explored in future quantitative studies to help determine the relative importance and usefulness of such programs in meeting World Health Organization goals for access to mental health treatment and quality community-based services.

**Electronic supplementary material:**

The online version of this article (doi:10.1186/s13033-016-0058-6) contains supplementary material, which is available to authorized users.

## Background

Mental and substance use disorders are the leading cause of non-fatal illness worldwide, with a higher disease burden than HIV/AIDS, tuberculosis or diabetes. They affect persons of all ages but have the highest impact on children over 10 years of age and young adults. These disorders are also the leading cause of years lost to disability (YLDs). Of years lost, 21 % are due to substance abuse problems and 70 % are due to serious mental illnesses [1].

Low and middle-income country (LMIC) governments struggle with limited resources and yet are expected to meet the great needs of their populations. Mental and substance abuse disorders account for 13.5 % of the global burden of disease [2], yet Central American governments spend only 1 % of their national health budgets on mental health needs. In contrast, wealthier countries spend 5 % [3, 4].^1^ This budget gap is indicative of a treatment gap, where individuals with mental illnesses go untreated even though effective treatments exist [5]. Of those in need of mental health services in LMICs, 75–90 % are unable to access those services from their national healthcare systems [6].

### The role of user and family groups

One low-cost intervention for persons with mental illness and their family members is self-help groups (SHGs) [7]. Mental health self-help groups, also known as mutual or peer support groups, are directed by people with mental illness (users) or their family members and provide education, support, empowerment, advocacy or similar activities for other families or users [8]. While paid professionals assist some groups, peer instructors, family leaders and other volunteers do much of the work [9]. Over recent decades there has been increasing interest in the importance of these programs and their benefits for individuals with mental illness and their families, as well as their ability to improve mental health system services, increase the use of evidence based practices, and increase funding for mental health research [10, 11].

Cohen et al. [7] review the quantitative literature from high income countries (HICs) to summarize the many benefits that SHGs bring for help group participants: decreased use of inpatient facilities [12], decreased levels of worry and depression, increased feelings of empowerment [13, 14], positive effect on social support and social networks [15], improved patient functioning and decreased caregiver burden [16, 17]. They note that the formation of SHGs has become an important component of mental health programs run by non-governmental organizations in LMICs [18], yet there is a lack of research on SHGs in LMICs.

A review of the literature by the authors showed that, despite over 15 years of existence of similar programs in a number of LMICs, there have been few studies to describe the programs or to demonstrate effectiveness in order to build an evidence base for best practices. The little research that is available is primarily qualitative and descriptive [7, 9, 19, 20]. Two quantitative studies carried out in India [21, 22] showed: (1) participation in self-help groups was an independent predictor of improved social functioning, e.g., voting, attending festivals, and working; and (2) medication adherence, having a family engaged with the program, and being a member of a self-help group were independent predictors of good outcomes.

### Need for this study

The focus of this study, the Family Education, Support and Empowerment Program (FESEP) in El Salvador, currently has no clear, identifiable outcomes or impacts which can be evaluated. Thus, this study is important to help program participants and leaders understand how and why their program may be effective, to assist them in building evaluation capacity, and to provide an opportunity for learning research.

Studies on self-help mental health programs in high-income countries (HICs) have generally reflected positive benefits, but have only focused on outcomes for individuals [8, 23–26]. Interventions carried out in LMICs are different because family support is often included and programs are carried out by nonprofits that focus on needs that are not being met by government services or programs. Program components may include supportive employment, self-help groups, community day programs and club houses, empowerment for advocacy, and other best practices typically provided in high income countries [10]. Cohen et al. [7] in a qualitative study of SHGs in Ghana concluded that self-help groups provide a range of supports (social, financial, practical), foster greater acceptance of service users by their families and by communities at large, and are associated with more consistent treatment and better outcomes for those who are ill. Lund et al. [27] reviewed BasicNeed’s health and development model in Kenya (medication, clinical follow-up, self help groups, occupational training, grants, referrals and counseling) to show improvements in global mental health, functioning, income generation and quality of life. Others have looked at participation in advocacy [28], or a mixture of professional treatment and self-help group participation [9, 20]. However, these studies provide little to no detail regarding how the groups were structured, what activities they carried out, who provided leadership, or the impact of the program at various social levels (individual, family, community, organization and society). The present study will provide a unique qualitative analysis on a wide variety of benefits and levels of impact for participants, from typical psychosocial benefits for individuals to social capital, leadership, advocacy, and organizational role.

### Purpose of the study

This study seeks to increase understanding of user and family self-help programs within the context of Latin America; to explore whether and to what extent the Family Education, Support and Empowerment Program (FESEP) is perceived as potentially effective and satisfying by its participants; to help the program build evaluation capacity and opportunities for program improvements; and to lay the groundwork for further quantitative study by identifying potential indicators to help evaluate program impact.

### Intervention description

The Family Education, Support and Empowerment Program (FESEP) serves people with mental illness and/or their families. Volunteer professionals and paid staff partner with user and family caregivers to facilitate the program. The FESEP program provides education through trained volunteer family instructors, a monthly associational meeting for support, education and advocacy, limited home intervention for member families in crisis, a weekly psychosocial group for persons with mental illness, very limited income generation support for users and their family members, periodic national forums on mental health and disability rights, opportunities for legislative advocacy and service on national health and disability rights commissions, and training of community workers in institutions that have a direct impact on the quality of life of users and family members, such as psychiatric hospital personnel and police officers [28]. The program is located in El Salvador, a LMIC. It is facilitated by a nonprofit organization, the Association for Training and Research on Mental Health (ACISAM), located in the capital city San Salvador. ACISAM functions with collaborative support from the Center for Health and Human Development (CHHD), a US-based nonprofit which acts as liaison to the funding foundation and provides support in the form of organizational capacity building, best practices and research. Partners in the program include two nonprofit user and family groups, the Association of Families, Friends and Persons with Mental Disability (AFAPDIM) and the Salvadoran Association of Families and Friends of Persons Suffering Schizophrenia and other Mental Disorders (ASFAE). While the program includes several components similar to the NIMH RAISE Early Treatment Program (for example, family education and supportive employment) [29], the program lacks funding to provide medications or individual professional resiliency therapy. Rather, it focuses on self-help, peer leadership/instruction, and individual empowerment to achieve individual and social change.

For the purposes of this study, researchers defined leadership from the perspective of participatory leadership. That is, a leader is anyone within the group taking responsibility for assisting others, facilitating group dynamics or decision-making, serving as a representative to coalitions or commission, or participating in the Coordination Team. A leader is not defined by position within the legal structure of the organization (authority), training, being an employee of the organization, or educational degree.

### Research design and measures

Researchers carried out a qualitative evaluation using a focus group [30–32] and used a technique that adds value to the program by helping it to determine potential outcomes and impacts, help program participants see what they have achieved [33], and help organizational leaders develop greater evaluation and research capacity [34].^2^

Researchers employed a combination of pragmatic and critical-emancipatory approaches [35, 36]. The specific focus group approach employed in this study is based on that used by Averett, Carawan and Burroughs [37] in which program participants and staff would make up the focus group together. The benefits of this approach include respect for the intervention setting, greater opportunities for allowing the research process to contribute to goals of pedagogy and empowerment of participants, and building relationships among the various stakeholders that contribute to everyone’s deeper understanding of the intervention, including that of the researcher. Creswell [35] refers to the researcher as a “complete participant” in this mode of observation.

Researchers assisted participants to complete an individualized questionnaire as a concurrent validity tool. The questionnaire included sociodemographic information and leadership and trust questions in the form of Likert scales. These questions were modeled after questions from the World Values Survey, but were not piloted.

For the focus group sessions, we asked a series of 11 closed to open-ended questions to the focus group. Examples of questions included: “What are the most significant changes that you have seen in others resulting from the program?”, “Why do some family caregivers benefit more from the FESEP program than others?”, “Do you believe that family organizations [in Central America] like ASFAE, AFAPDIM and Cuenta Conmigo are important? Why?”, “How does the FESEP program compare with other services and programs available to users and families?”, and “How has the FESEP program helped to create greater social capital for individuals and the organization (both linking and bonding capital)?”. Questions were fully explained and participant questions responded to before launching into open discussion.

We managed interpretation through a group of three investigators from two different cultures, and we shared moderator responsibilities between a lead and an assistant moderator.

Creswell and Miller [35, 38] recommend that researchers engage in at least two of eight validity strategies for qualitative studies, this study engaged in six to try to strengthen the qualitative validity. Both APA Method guidelines [39] and the Consolidated Criteria for Reporting Qualitative Studies (COREQ) 32-item checklist [40] were used as guides in the preparation of the study’s methodology.

### Study population and sampling

According to data from 2014, 124 users and caregivers participated in various program components; within the weekly art therapy component for users, 8 of 28 persons were new (29 %); and within the family education component, 20 of 27 were new (74 %). Of 39 users in who participated in any program component, 72 % had schizophrenia, 13 % bipolar disorder, 8 % depression, 3 % anxiety, and 5 % other (mental retardation, autism, or undiagnosed).

Participation rates for most program components were not tracked at the time of the study, but a recent separate analysis of the participation rate of family caregivers in the “From Family to Family” education class showed participants attended an average of 36 % of the classes, a low participation rate. The most prominent barriers to better participation were inability to find others to care while the family caregiver attends the class, cost of transportation, inability to take time off every Saturday morning (evening classes are considered too dangerous, and public transportation stops running by 8 pm). For lack of epidemiological studies of mental health in El Salvador, we do not know how many people with mental illness there are in El Salvador or how many lack services. But applying one global estimate of 10 % would put the number of people with mental conditions at over 630,000 in El Salvador [41]. Tripling that number to 1.9 million would indicate how many family members/carers are potentially impacted by mental illness.

The focus group met twice in March 2013, and was composed of ten persons: three persons with diagnoses of schizophrenia (two men and one woman), four family caregivers (three female siblings and one mother; all had loved ones with a diagnosis of schizophrenia), and three program staff (a paid male psychologist, a volunteer female psychiatrist, and a volunteer program founder who is also a male caregiver with a brother with schizophrenia). User and caregiver study participants represent 10 % of the population of active program participants at the time of the study in early 2013. The program staff and research team are the same, and are composed of an American lead investigator who has volunteered with the program for 12 years and two Salvadoran co-investigators (psychologist and psychiatrist).

The sampling strategy was to identify three types of participants—users, caregivers, and staff/researchers—to facilitate comparisons among subgroups [38]. Criteria for focus group participants was jointly arrived at among the researchers. Inclusion criteria included being a user, family caregiver, or program staff or volunteer; being active participant of the program; and having substantial experience with a variety of program components over several years. Exclusion criteria included lack of illness stability and inability to communicate well one’s personal thoughts and feelings. Users and family caregivers were selected by the Salvadoran nonprofit mental health organization (ACISAM) that runs the intervention and has positive long-term relationships with users and family caregiver participants. Table 1 outlines selection criteria for user, family and professional subgroups (such as emotional stability, ability to articulate and share one’s thoughts, and longevity of participation) and characteristics of age, gender, and illness type. Participants were contacted by phone or in person. All those approached agreed to participate in the study and signed informed consent (for the consent form see Additional file 1). Focus group sessions were later held in a quiet, comfortable salon that provided lunch and snacks. None of the participants dropped out of the study.

**Table 1:** Participant demographics and selection criteria

Users	Family caregivers	Professionals
*Age range*: 21–45 years (average: 37 years) *Gender*: two males, one female (three persons total) *Illness*: all users suffered from schizophrenia *Selection criteria*: length of time participating in program, illness stability, and ability to communicate thoughts and feelings	*Age range*: 26–62 years (average: 37 years) *Gender*: no males, four females (four persons total) *Relationship to persons with mental Illness*: mother, daughter, and two siblings *Selection criteria*: length of time participating in program, emotional stability, and breadth of program participation	*Age range*: 37–53 years (average: 46 years) *Gender*: two males, one female (three persons total) *Principal investigator*: American white male, program founder, family caregiver, 17 years of program experience in El Salvador *Salvadorian professionals*: male psychologist and female psychiatrist *Selection criteria*: length of time participating in program and maximum variation of program role

Participants ranged from 21 to 62 in age, six were females, four were males, and all three users had the illness of schizophrenia while the family caregivers likewise had loved ones who suffered from schizophrenia, although the selection for this illness was not purposive but rather indicates the preponderance among program participants of this illness type. Participants were selected for stability, ability to communicate, and breadth and length of time participating in the program

### Data collection and analysis

Individualized questionnaires were completed by all 10 members prior to the focus group meetings in order to collect demographic and Likert scale responses on program effectiveness, satisfaction and social capital (measured by trust). A researcher was present to assist each person in completing the written questionnaire and clarify meanings (for the individualized questionnaire see Additional file 2). Researchers met to discuss questions and discrepancies in the responses. A second questionnaire was used to guide the focus group discussion, which was led by the principal investigator with assistance from the co-investigators. Questions were provided to the participants (but not piloted) and ranged from closed to open-ended. To minimize adverse power-dynamics, users responded first to questions, followed by family caregivers, and finally by professionals. Then the space was opened for anyone to comment again. This process allowed users to dominate the process and gave the different subgroups a chance to react to others’ comments, providing a sense of where subgroups had commonalities and differences. It was not necessary to repeat any assisted questionnaires or focus group sessions.

The observational protocol included recording on two devices, summary comments written on large paper for all to see, and the PI’s written notes. Recordings were transcribed (for the full anonymous transcript in Spanish see Additional file 3). The lead researcher and two Salvadoran co-investigators performed analysis, and themes were determined by consensus. Researchers first highlighted significant statements (“horizontalization”) and then organized the statements into “clusters of meaning” [38]. Conclusions were shared with focus group participants and others in the program for comments and reflection. The final write-up was approved by the Salvadoran co-investigators in order to minimize cultural bias and clarify meanings across two cultures/languages.

### Language

The questionnaires were first developed in English according to content related to studies in a number of fields (mental health studies, social capital, leadership, organizational behavior/development). The questionnaires were then translated into Spanish and the two Salvadoran researchers worked with the US lead researcher to ensure that the meaning was both accurately translated and would be clearly understood by focus group participants in the Salvadoran context. The data in the original Spanish was the basis for the summaries and analysis of the data.

### Ethical considerations

Risks and risk mitigation were addressed in the consent form (see Additional file 1). Participants received a $10 payment for each focus group session in order to reimburse transportation costs and partially off-set losses of income due to the focus group sessions, which were each half-day in length.

All investigators received human subjects research certification, either via James Madison University or a Spanish version hosted by the US National Institutes of Health. IRB protocol #13-0340 was approved by James Madison University Institutional Review Board.

## Results^3^

### Results related to the individual questionnaires

#### Program participation

The individual questionnaires demonstrated that focus group members were diverse in gender, age and stakeholder mix, but less so in relation to illness type. Focus group members generally reflected the lower end of the socioeconomic scale despite having a high educational level overall.

Program participation averaged of 5.5 years. Focus group members in all subgroups had moderate to high levels of participation in at least four program components (Table 2). All family members had completed the education class, and all users had participated in the weekly psychosocial user group. Family caregivers struggled to get their ill family member to attend the psychosocial group due to denial or illness severity. However, frequently the family member and professional support staff were able eventually to achieve participation by loved ones of family caregivers.

**Table 2:** Program participation—by program component and duration (in years)

Sub-group	Family caregivers	Users	Professionals
Family education class (total years)^a^	4	0	3
Average years	1	0	1
Monthly support Group (total years)	23.5	15^b^	22
Average years	5.9	5	7.3
Public awareness projects (total years)	12	2	22
Average years	3	0.7	7.3
Advocacy national service (Total years)	17	13	14
Average years	3.4	4.3	4.7
Visits to homes in crisis (total years)	2	1	18
Average	0.5	0.3	6
Psycho-social group (total years)	0	21	0
Average years	0	7	0
Other (total years)^c^	14	18	30
Average years	3.5	6	10

Comparison of users, caregivers and professionals by duration of participation in principal program components

^a^ Total years refers to the sum of years that all family caregivers together obtained; average years refers to the total years divided by the number of persons in the subgroup

^b^ Users state their participation was often sporadic, and some numbers had to be estimated by the researchers due to uncertainty in responses by some users. Due to these factors, the numbers listed here for users may be high

^c^ Trainings of professionals, participation in national annual forums

#### Improvements related to FESEP program participation

Regarding illness change over time, most replied that the user had experienced ups and downs in stability, but several also noted clear improvements, which they attributed at least in part to participation in the FESEP program (see Table 3 for exemplary quotes). Some improvements were indirect for users, such as when a user was non-compliant and uninterested in participating in the FESEP program, but family member participation resulted in perceived increase in understanding and better family treatment toward the ill person in the home.

**Table 3:** Improvements related to FESEP program participation

Subgroup	Comments
Users	Since I began to participate in FESEP I feel acceptance, I feel useful, I occupy my time, and I get moral support
	Now I don’t sleep so much of the day. My family situation has improved because everyone is participating in the program, including the other person with a mental illness, so there are not big fights now. There’s more income, more understanding between us. I get out of the house to go to the program. Family members are not so demanding and directing because they understand of my condition
	I’m able to relate to others now. I respect my grandfather. I coexist with others and think positively. I am happy. I have friends
Family caregivers	Our family members now have an understanding of my brother. We look for creative ways to treat him. For example, we hide his morning meds in his oatmeal
	Now we can talk and eat together, we laugh together. My older son stopped smoking. Home crisis intervention by ACISAM professionals was very helpful once when the police had to be called to take my psychotic son to the hospital

Quotes from users and caregivers related to feelings of being accepted, happy, useful, improved family dynamics and income, increased understanding, ability to relate to others, to have friends, to enjoy family, and to have support in times of crisis

#### Leadership development

Focus group subgroups differed sharply in their leadership roles and development (Table 4). Professionals had high levels of leadership responsibilities and experience, while family members had moderate levels and users had very low levels of leadership. Counting years according to program components, family caregivers totaled 35 years of leadership between four people, while the professionals totaled 121 years between three people. The three users totaled only 2 years. Family caregivers were strong in the areas of training for and acting as class instructors, coordination of monthly support groups, and facilitation of advocacy.

**Table 4:** Program leadership—by program component and duration (in years)

Sub-group	Family caregivers	Users	Professionals
Family class instructor (total years)^a^	7	0	17
Average years	1.8	0	5.3
Support group coordination (total years)	10	0	10
Average years	2.5	0	3.3
Public awareness projects (total years)	3.5	2	15
Average years	0.9	0.5	5
Board/coordination team (total years)	5	0	7
Average years	1.3	0	2.3
Advocacy national service (total years)	6.5	0	26
Average years	1.6	0	8.7
Psycho-social group (total years)	2	0^b^	16
Average years	0.5	0^b^	5.3
Other (total years)^c^	0	0	40
Average years	0	0	13.3

Comparison of users, caregivers and professionals by duration of leadership roles in principal program components, for example, coordination of public awareness projects, or service on coordination (leadership) team, or facilitator of the psycho-social group for users

^a^ Total years refers to the sum of years that all family caregivers together obtained; average years refers to the total years divided by the number of persons in the subgroup

^b^ Users stated they provided encouragement and listening to their peers, but did not consider themselves to have decision making power or to be leaders

^c^ Homes visits facilitation, leadership on fund raising, strategic planning with ACISAM and AFAPDIM, etc

Users did not perceive themselves as leaders. They stated that they did not participate in leadership for monthly support groups, as board members of the association, or as part of the Coordinating Team. Nor did they act as peer facilitators of the psychosocial group. Most of these components were facilitated and led by family caregivers. Regarding the psychosocial group, one user reflected a long time on whether or not he was a leader in this group and finally stated that while he was not the one with deciding power, he did help others by encouraging them not to abandon their treatment and providing a listening ear. However, he did not recognize these as peer leadership abilities. He stated his role in the group in terms of “compañerismo.”^4^

#### Participation in services and programs beyond FESEP

Focus group members’ participation in mental health programs beyond FESEP was minimal. While there has been participation in other interventions, there was a generally high level of dissatisfaction with those experiences. Examples included (1) a caregiver who at first went to “charlatans and witches” who promised they would cure her two sons, but now she see these were “stupidities and rip-offs”; and (2) the psychiatric hospital’s day program. Positive experiences included workshops by other non-governmental organizations (NGOs), treatment at a private hospital, support at church, and a short-lived day program that was closed because it was too expensive to maintain clientele.^5^

#### Program impact and satisfaction

Participants responded to several questions using a Likert scale. For example, for program effectiveness we used the following terms: 1 = not effective, 2 = little effectiveness, 3 = moderately effective, 4 = effective, 5 = very effective.

Effectiveness, satisfaction and sense of belonging were all rated highly. Effectiveness of the FESEP program at improving the mental health wellbeing for program participants was rated 4.5. Level of satisfaction with the program had the same rating. Sense of belonging had the highest response rate across subgroups, with an average of 4.7, indicating that all subgroups had a strong sense of belonging or “community” in the relationships created in the group (Table 5).

**Table 5:** Levels of perceived program effectiveness, satisfaction, and sense of belonging

Sub-group	Family caregivers	Users	Professionals
Program effectiveness	4	4	4.7
Program satisfaction	5	5	4.7
Sense of belonging	4.7	4.7	5

Comparison of users, caregivers and professionals using a Likert scale (1–5) to indicate perceived sense of effectiveness, satisfaction and sense of belonging that the program represents for each individual. For example, the first two questions read “How effective is the program at improving mental health wellbeing for families and users of mental health services?” and “How satisfied are you with the program?”

#### Civil society participation and levels of trust^6^

Participation was low in other civil society groups: one family caregiver participated in three (all related to disability), but two other caregivers and one user each participated in only one other civil society group (two of these were church communities). Four people did not participate in any civil society association outside of the FESEP program. For some, participation in FESEP was their first experience in a civil society group.

Regarding levels of trust, we asked further Likert questions related to generalized trust and trust related to specific mental health programs and institutions as measures of social capital (Table 6). We used the following terminology: 1 = you can never trust in…, 2 = rarely can you trust in…, 3 = sometimes you can trust in, 4 = often you can trust in…, 5 = you can always trust fully in…. The focus group’s average level of trust was 3.3, that is, this sample of program participants felt that they could sometimes or often trust in others.

**Table 6:** Levels of interpersonal and institutional trust

Sub-group	Family caregivers	Users	Professionals
Generalized trust	3.5	3	3.3
Private providers	3.5	3.5	3.7
National psychiatric hospital	2.8	3.3	2.7
ACISAM facilitating NPO^a^	4.5	4.7	4.7
AFAPDIM family/user NPO	3.3	4.7	4
CHHD foreign support NPO	4.5	5	5

Comparison of users, caregivers and professionals using a Likert scale (1–5) to indicate levels of interpersonal and institutional trust

^a^Non-Profit Organization is the same as NGO—Non-Governmental Organization

In terms of trust in specific organizations, international NGO collaborator CHHD was highest at 4.8. This was closely followed by the professional nonprofit ACISAM at 4.6. Trust in the family association AFAPDIM was 4.3 with family members’ trust in their own organization much lower than users’ trust. Private practice providers were rated at 3.6. Next, nearly a point down, came the national psychiatric hospital at a moderate 2.9, with users trusting more in the hospital than family caregivers or professionals. Finally at 2.7 and 2.6 (rated below “sometimes you can trust in…” by all three subgroups) were the Salvadoran mental health system and the Salvadoran government.

### Results related to the focus group sessions

We asked a series of 11 questions to the focus group. Detailed discussion of the responses are covered in the Additional file 4. Below we note the program benefit themes, divided into micro, mezzo, and macro social levels. We provide examples of quotations for each social level.

At the micro level we identified a number of themes related to individual benefits that people experienced through the program. These include improved quality of life, improved ability to participate in social groups outside the home, increased income earnings, improved self-esteem, improved abilities to deal with illness management, increased illness knowledge and acceptance, awareness of the illness and one’s ability to deal effectively with the illness (self-transformation), increased ability to communicate about mental illness with others in the broader society, increased human rights awareness and opportunities to address stigma (societal transformation), the discovery and development of leadership abilities, and increased purpose and meaning (see Table 7 for exemplary quotes).

**Table 7:** Individual level achievements (micro level)

Subgroup	Comments
Users	The program keeps me occupied
	The program channels my energies
	I receive personalized attention for dealing with my problems
	I learn about myself, my problem, and I find answers that help me to overcome the agony
	I felt my self-esteem grow when I began to earn money
	One feels useful, from the family to the organization and even for our society
Family caregivers	The program helps unburden family caregivers
	One can speak freely and express hidden feelings
	We find support in the program
	I receive help in emergency moments of crisis
	I feel this is my family; I can cry here
	I feel accepted and free to be myself
	Understanding and insight, for the illness and user, and for going to the streets to defend our rights in public protests
	Our empowerment evolves; we grow with time and practice
	I discovered I can help others
	I discovered a different way to working—in a group. There is no boss looking over my shoulder

Users and caregivers expressed individual level achievements and benefits of participation, including keeping occupied, channeling energy usefully, personal attention, self-understanding and self-management, improved self-esteem, feeling useful to others and to society, chance to unburden oneself and express feelings, find support, understanding the illness, opportunities to advocate for systemic change, increasing sense of empowerment, ability to help others

At the mezzo level, program benefits themes included improved family communication and relationships, establishment of a legal organization for the interests of consumers and families, increased bonding and bridging social capital, the provision of opportunities for participatory leadership, and perceived reduction of stigma in the community (see Table 8 for exemplary quotes related to family benefits/achievements, Table 9 for quotes related to community, and Table 10 for quotes related to organizational benefits/achievements).

**Table 8:** Family level achievements (mezzo level)

Subgroup	Comments
User	I learned to improve my relationships. I’m not jealous of my wife anymore
	I cooperate and help out more, like going out to buy tortillas and sweeping
	I don’t fight with family members now. We have lower levels of confrontation
	I feel less pressure and demands by my family members on me
	The program helps me to try to improve relationships with some family members who are indifferent and condescending towards me
Family caregiver	Stress relief: we can rest because the user is not in the house all the time. Families also don’t get bored of the user, we can enjoy our ill loved one more
	The level of understanding goes up in the family and this is transmitted and felt by the user too
	Life is easier in the family. Learning how to care for my two schizophrenic sons has helped us all improve our communication in the family with my deaf daughter
	I try to share what I’ve learned with other family members
	The program helps with unification of the family as myths and blaming disappear
	There is improved coordination of care by family caregivers
	Family members learn to respect the user and become more tolerant of the user’s behavior
	Families understand and fight against user dependency (co-dependency and dependency issues), and against their own caregiver burnout
Professional	As a volunteer, the program has helped me to create a conscience in my children, to the point where they encourage me to let go of family time and do my volunteer work with the program

Responses reflected benefits at the family level. Users learned to improve family relationships, to help out, and to reduce fighting. They also feel empowered to deal with critical and demanding family members. Caregivers feel less stress and enjoy their loved one more. They experience greater levels of understanding, communication, and motivation to share what they’ve learned. They are able to identify myths, to coordinate care better, to deal with their caregiver burnout, and to respect the human rights of their loved one. Professionals describe benefits gained through volunteering

**Table 9:** Community achievements (mezzo level)^a^

Subgroup	Comments
User	I have a relaxed life in my neighborhood. People greet me. I feel good in the street
	Before, I was in the house because neighbors could not stand me. But now we talk. They even encourage me to keep making hammocks
	I know now how to avoid neighbors to avoid problems when I go out
	I get along with everybody, I’m proud and share it with church friends, that I can leave the house on my own to go to art therapy and go out looking for work
	I have a friend now in my neighborhood. It’s easier to talk to people
	Some people say I’ve changed dramatically
Family caregiver	Now I’m not afraid that my son is not going to return when he goes out
	We’re better understood by others in the community
	I have more insight now. I can talk with whoever about mental health
	I am more empathetic with other in the community, especially families with disabled persons
	The program has helped improve dialogue, knowing how to listen, to respect the opinions of others
	It’s helped to create a shield I can use to discern when someone wants to help or not
	The program has helped us to confront the community on mental health. We’re able to overcome stigma to be able to talk with others. We don’t feel attacked but empowered to relate to others, to create greater understanding about human rights
Professional	When we enter dangerous neighborhoods like “Italia,” [the gangs] don’t bother us because they respect our work
	Psychiatrist: After working with this program, I would like to work at the community level and not in the psychiatric hospital

Participants reflected on benefits experienced at the level of their community or neighborhood. Users feel they are treated as normal people now, not trapped inside their homes. They know how to manage situations and are able to make friends. Neighbors are sounding boards who affirm how users have changed or improved. Caregivers are not afraid when their loved ones go out, they feel understood by others in the community, are empathetic with others who have disabled family members, have improved ability to listen to others, can create a shield of protection against those who would be destructive towards them, and are able to overcome community stigma to be able to talk with others. They feel empowered to relate to others in order to create greater understanding about human rights. Professionals said they can often enter dangerous neighborhoods because their work is appreciated. One now wants to work now at the level of community services (rather than in a clinic or hospital)

^a^ Community is broadly defined here, referring to achievements in the neighborhood, but also other communities of support such as church communities

**Table 10:** Organizational achievements (mezzo level)

Achievements	Comments
Across subgroups	The program promotes horizontal leadership
	It develops skills to self-organize
	The formation of our group [as a government-recognized nonprofit organization] facilitates advocacy and participation at the governmental level. We are empowered to fight for the defense of human rights
	We are no longer invisible; we have an identity
	It develops our understanding of the importance of self-organization to resolve problems, like the Medications Law^a^
	It promotes collaborations with other nonprofits

All subgroups of participants agreed that there were organizational benefits at the mezzo level as well. These included that the program develops horizontal leadership, the ability to organize, and that this results in the establishment of a formal group and advocacy by the group. Rather than feeling invisible, they feel they have an identity as a respected organization and this results in collaboration with other organizations and achievements in advocacy. Achievements through the development and functioning of an organization

^a^ The “Ley de Medicamentos” (Medications Law) is a law that was proposed to reduce the exorbitant cost of medications in El Salvador. Members of the FESEP program joined many other civil society groups in holding forums and marching in street protests calling for passage of the law. For the mentally ill and their family members, this was a huge achievement, moving from stigmatized isolation to public protest. Despite significant odds, the coalition of groups succeeded in passage of the law in early 2013

At the macro level, benefit themes included increased advocacy on a national level for the rights of those with mental disabilities, changed laws and policies, and attainment of a national reputation as the civil society voice for those with mental illness and family caregivers (see Table 11 for exemplary quotes).

**Table 11:** National achievements (macro level)

Achievement	Comments
National level advocacy	The program has opened spaces for participation in advocacy, which is really important because there are no other organizations in the country working in the psychosocial area
	We are now established as the non-governmental mental health entity in national forums
	Advocacy as members of the CONAIPD coalition. Participation [in this governmental and NGO advocacy council] has been a great way to relate to other nonprofits in the country. To be part of a large network increases our impact. It allows us to educate other nonprofits about mental health and shows users and their family members are part of the movement for disability rights too^a^
	We are positioned in the civil society. The Ministry of Health has taken notice of us and invited us to participate in the first revision of the 2008 national law on mental health. The Pan American Health Organization (PAHO) invited our representative members to participate in international conferences in Panama (2010) and Brazil (2013)
Anti-stigma activities and advocacy	Educational and sensitization trainings directed at the sectors of society that work with users and families in the community, such as programs with the national civilian police force and national psychiatric hospital workers about the human rights of persons with mental health problems, the role of the family as partners in treatment, the needs of families in the community, and information about our FESEP program that serves as a referral resource for police, hospital personnel, and public health clinic workers
	National forums that have brought together diverse sectors of society
	We’ve worked to sensitize society through the media, especially radio. I think our society is more educated about mental health and there is less stigma
Needs of users	We’ve identified and promoted needs of users that have not been identified by the government, for example, education and work opportunities
Thinking big	We are growing. There are more groups in El Salvador now than before
	We need groups all over Central America to help users and families in other countries, to promote advocacy, and to form international networks to strengthen our advocacy

Participants identified national macro level benefits and achievements. These included the opening of spaces for advocacy on a national level, participation as members of national commissions for disability rights and health care reform, holding national forums to highlight mental health needs, and awareness trainings held for health, security, and other professionals. These are important achievements because no one else is advocating for disability rights from the psychosocial perspective. As a result of these and radio programs, they feel they have reduced stigma in the country. They are also recognized by and invited to events of the ministry of health and the Pan American Health Organization on an international basis. They recognize there are more groups now than ever in El Salvador, and they are starting to assist sister groups in other countries

^a^ CONAIPD (Consejo Nacional de Atención Integral a la Persona con Discapacidad) (National Council for Integral Attention for the Person with Disability) is the lead entity in charge of federal guidelines related to disabled persons and coordination for actions in support of this population

## Discussion

### Analysis related to the pre-focus-group individualized questionnaire

Users have been slow to develop leadership in the FESEP program. The cause of this was not determined, but factors may include internal and external stigma,^7^ ACISAM’s slowness at turning responsibility over to users due to traditional authoritarianism, the severity of the illnesses with which users struggle, and the dominance of family members, several of whom are very strong leaders and highly educated.

**Fig. 1:**
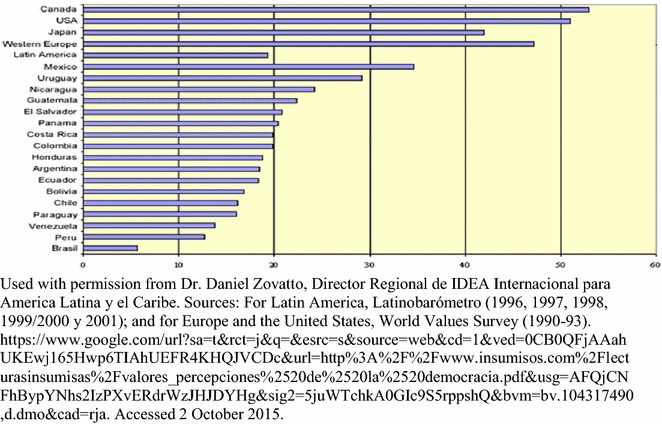
Levels of interpersonal trust in Latin America (average 1996–2001). The World Values Survey and other international surveys ask questions about trust towards other people as a reflection of each society’s level of social capital. Measures of social capital reflect levels of satisfaction and efficiency that citizens feel towards their governments and other institutions within their societies. Social capital helps institutions function well and achieve goals that citizens want. Trust, then, is a major means of measuring how well people can work together to accomplish larger goals. This chart shows that El Salvador is in the middle of measures of interpersonal trust among countries in the Americas, with Canada and the USA having relatively high levels of trust, and Peru and Brazil at the bottom. At 21 %, people in El Salvador have low levels of trust in others. This study compares a small sample of users and caregivers in a community mental health program very favorably against this measure of trust from the general Salvadoran population, with the average response falling between one being able to “sometimes” or “often” trust in others. The question then arises, do community self-help groups and organizations help to create higher levels of social capital among participants that facilitates more effective and satisfying organizations? Does increased social capital at both the individual and organizational levels help these organizations to accomplish their goals?

The group had moderate to moderately high generalized trust of others, with 30 % stating one can often trust in other people. This appears to contrast with World Values Survey results for El Salvador that were averaged between 1996 and 2001 [42]. That analysis showed that only 21 % of Salvadorans generally have trust in other people (Fig. 1).

Users in this small study tended to trust at higher levels than families or professionals when asked about places they received services (nonprofits and psychiatric hospital), yet they had lower generalized trust, which refers to how they are treated by people in the wider society. This is a dynamic that researchers of social capital and trust should take into account—that different types of people, specifically those with mental illnesses, will have different levels of trust towards different people and institutions due to their experiences of stigma as well as assistance from those institutions.

Overall, responses regarding generalized trust and trust in private mental health services was moderate, while trust in the government and governmental mental health services was low. Trust in nonprofit programs benefiting people with mental illness and their families was very high. For example, one user noted,

“If you go to the hospital to see a doctor and your case is not grave, they don’t attend to you. Hospital workers try to avoid working hard in both public health clinics and hospitals. The situation is the same in both the mental health system and the general health care system. Workers complain we patients are not cooperating, not making an effort, but it’s not true. If we’re not taking our meds or going to follow-up appointments it’s often because we don’t have the money to do so. The psychiatric hospital staff doesn’t trust anyone. They are more concerned with themselves than with the patients.”

Comparison of educational levels of focus group participants revealed that both user and family subgroups had several participants with professional degrees. Calling the staff and volunteers “professionals” as a group differentiator is thus a misnomer and reflects bias on the part of we researchers. In future studies we recommend changing terminology to “non-family volunteers and staff,” although participants have also recommended “friends.”

Despite many participants having higher education, only the professional group had stable employment. This reflected challenges faced by focus group members who have mental illness or caregiver commitments to care for loved ones with mental disabilities limiting their ability to obtain and maintain employment. It is consistent with World Health Organization and International Labor Organization reports on extremely high unemployment rates for those with mental disabilities [43]. According to a family leader in Ecuador, family caregivers of persons with disabilities in that country are eligible for government funding at $300/month to offset these types of challenges and costs (personal communication, Marta Monge, October 16, 2013), a model the authors recommend for consideration in other countries.

### Analysis of data from the focus group sessions

In general, focus group analysis indicates that participants responded positively to questions about the program due to the program’s ability to meet their needs, to transform their perspective through new understanding, and to provide them with tools to address the challenges of mental illness. We identified a large number of themes related to the benefits of program participation across micro (individual), mezzo (family/community/organizational), and macro (national/society-wide) levels.

This study potentially indicates benefits such as increased and effective advocacy, inclusion of persons with disabilities, perception of reduced stigma in both communities and across society, and civil society development. Focus group members felt an identity through the organization and a sense of common purpose, which we think may be heightened through the methodology of supported peer leadership and service provision.

Family support repeated surfaced as a benefit. It was seen as facilitating both improvements in family relations within the home and moving the user toward participation in the program, from which the user could progress on many fronts. This is consistent with research related to the influence of family attitude on relapse (research on expressed emotion and family psycho education [24, 25, 44] and the interactive influences of stigma and self-stigma [45, 46], wherein the family and larger society can impact a user’s beliefs about himself/herself).

For this focus group sample whose users and caregivers’ loved ones all had diagnoses of schizophrenia, responses demonstrated that the FESEP program is achieving its goal of improved mental health wellbeing. Focus group members, whether users, family caregivers or professionals, all expressed deep feelings regarding the positive impact the FESEP program had on them across social, functional and emotional dimensions. The perceived benefits were often tied to their position within the program, reflecting different needs for users, family members and professionals. It shows that this type of multi-component program may be able to address a wide variety of needs for people connected in different ways to mental illness. Participants clearly and easily delineated program benefits according to social level analysis.

Focus group members agreed on the importance of leadership to organizational success and outlined leadership qualities, roles, and methodology that contributed to this success. Special emphasis was given to the perceived benefits of horizontal leadership. Family members felt leadership was shared, and a variety of participants were offered opportunities for responsibilities and leadership roles.

All subgroups of the focus group identified the program’s participatory methodology as a key influence for creating a variety of benefits, which is reflected in similar studies on user and family programs in HICs [7, 10]. Specifically, the peer model of organizational work with professional facilitation/support used by the FESEP program in El Salvador has been successful at developing leadership that is horizontal, shared, participative, and empowering for participants. It is a methodology that allows participants to discover and develop leadership skills. In the context of disabilities, it has been found to be particularly useful in helping stigmatized populations to improve physical and mental health [47].

Social capital was recognized as important. All subgroups agreed that the organization had done tremendous work increasing its social capital (bonding internally and bridging trust and respect with other groups and networks across society, especially within the disability rights movement), but that more remained to be achieved. We think social capital is essential if groups are to assume a leadership role in grassroots advocacy for change in mental health systems.

Participants reflected progress in understanding illness and acceptance of illness, both of which are key to successfully participating in treatment [10, 48–50]. They also showed an awareness of human rights for those with mental illness, which one professional called “un paso gigante,” a giant step. This is related to another conceptual achievement, the growth in self understanding developed over years that has allowed users and family members to learn how to “transformarse.” This word has multiple meanings—to evolve, turn around, change into, and become. The idea is that participants have changed from being afraid and isolated to being public advocates, from feeling lost to gaining insight and understanding and finding community, and from living in conflict to widely improved social relations. It’s a transformation not only at the personal level of understanding an illness but also at the societal level of seeing the need for and becoming involved in advocacy for systemic change.

Comments reflected how this transformational change results in practical gains—people take on responsibilities in the group, they develop leadership skills, and they offer their voluntary services [51–54]. Such individual and social transformation was perceived as a significant achievement because of the extent of stigma against those with mental illness in the Salvadoran society. Only recently through the efforts of the FESEP program have persons with mental illnesses been recognized by the government and by the NGO human rights community as an additional group of persons with disability rights in El Salvador.

### Linking individual and focus group data

Generally the data from the individual questionnaire and the focus group data corresponded well, especially related to high levels of support for the FESEP program, defining a broad array of benefits, and seeing those benefits across multiple social levels. While the focus group espoused horizontal leadership and leadership development, the reality reflected in the individual questionnaires showed that in fact was quite different for users. They did not perceive themselves as leaders and had not been given leadership positions in the organization. As a result, their social capital was not being fully realized.

### General observations

Group interviews are recognized as having multiple benefits, including stimulating discussion, opening up new perspectives for the participants, hearing the ideas of others helps participants to formulate their own opinions, and encouragement to speak for marginalized participants [55]. We found these benefits in our focus group. During the focus group sessions, the authors observed how the process methodology of bringing together users, family members, and professionals contributed to everyone gaining an understanding of the program’s benefits for others in the group. Authors also observed how the process methodology contributed to learning about benefits they had not heard of or thought about, and gaining an increased appreciation of the program.

The facilitation and support of ACISAM (Salvadoran NGO) and CHHD (international NGO) were perceived as key to the sustainable development of a quality program for families and users that fostered their own development as leaders and as advocates in the defense of their rights. We note that the methodology employed by ACISAM in facilitating the FESEP program is similar to the bio-psycho-social model of community based rehabilitation promoted by the Pan American Health Organization, which carries the following benefits: empowerment, participation, strengthening of civil organizations, decentralization of services for accessibility, and multi-sectorial participation [56].

In our study, professionals and support organizations were strategic partners with families and users. Chien and Norman [25] reviewed 25 studies in HICs that usually included the participation or leadership of nurses and other professionals. Programs in LMICs that focus on community health workers and other non-professional community level workers who are successfully carrying out “task-shifting” work in lieu of professional mental health workers who are in short supply in LMICs [57–59] demonstrate the important involvement of professionals and semi-professionals to address the global treatment gap for mental health services. However, the methodology of professionals acting not as trainers or therapists or task-shifters but acting as allies, facilitating the development of user and family leadership and organizations in LMICs, continues to need further research.

### Suggested program improvements

We identified suggestions for program improvements: further development of user leadership skills and increased participation for users in leadership positions; incorporation of logic model outcomes and impacts into planning, evaluation processes, and organizational reports; strengthening the employment component of the program; and continued nurturing of trust with key mental health institutions in the country. The high levels of trust in NGO mental health programs serving users and families should be leveraged to work more closely with low-trust institutions such as the government and the institutions of the mental health system, because these institutions are key to systemic change.

### Building program evaluation capacity

All focus group participants were thus able to gain knowledge regarding how and why their program may be effective, as well as what outcomes might be useful and measurable for future program evaluation that could include their participation. Salvadoran co-investigators developed knowledge and skills in helping to carry out this type of research, including standards for research on human subjects, and established a foundation for future work on evaluations and research for their organizations and programs.

### Organizational and policy implications

The study highlighted the need for community based mental health organizations in Latin America to improve evaluation processes by focusing more on outcome and impact measures and to involve professionals and program participants in research in order to build research capacity. This could result in an appreciation for the important role that they themselves must play in program improvements and the identification of best practices for mental health within the Latin American socioeconomic and political context.

Users and family caregivers are a potential large pool of volunteers to assist in carrying out community based programs such as support, education, income generation, and advocacy. They are a powerhouse of volunteerism that the government should nurture and support as it seeks low-cost ways to address the gap in treatment and services. User and family self help groups, organizations and programs appear to have a dramatic impact on the mental health wellbeing of both users and their family members, and these integrated, multi-component programs should be an important part of national collaborative interventions. Lund et al. [27] state that economic empowerment and social inclusion can have a substantial impact on clinical outcomes, functioning, quality of life, and economic outcomes for people living with severe mental disorders in conditions of poverty. Thus, governments should support such grassroots organizations by including them in policy discussions, strategic planning, and human rights commissions, and support structures that nourish and sustain such groups, including financial support, which is common in HICs.

Combining best practices in community-oriented psychiatry with best practices in organizational development for civil society mental health groups could result in significant improvements in mental health systems for LMICs. Partnerships between multiple stakeholders that include those with the most at stake (users and their family caregivers) hold promise for achieving significant change—improved services and closing the treatment gap for persons with mental illness and the budget gap for government health ministries.

It is clear that governments in LMICs cannot address the challenge of mental illness alone. But nor can the nonprofit and civil society sectors. Together they must address the gap in public awareness around mental health needs, stigma, and lack of services. National mental health care budgets need to be drastically increased, services decentralized and improved, and marginalized users and families placed in the center of the mental health care model.

### Recommendations for future research

This is the first study to explore potential benefits of these groups across micro, mezzo and macro social levels and to include discussion of more diverse potential benefits such as individual and organizational social capital, leadership, and advocacy. These are factors that should be explored in future quantitative studies that could confirm the broad types of benefits and effects of those benefits for these populations. Such studies should contrast these impacts with treatment as usual and cost effectiveness in order to help determine the relative importance and usefulness of such programs in meeting World Health Organization goals for access to mental health treatment and quality community-based services [60, 61].

Based on the outcomes of this focus group study, we recommend further study, particularly quantitative and mixed methods studies of the multi-component FESEP and similar programs in order to establish causality and generalizability. Two other studies of similar programs call for randomized trials, longitudinal and qualitative descriptive studies of people who participate in these types of programs [7, 27].

Programs vary widely from country to country. Until recently across Central America these programs had little contact to share program models, knowledge, experiences or resources. As a result, further comparative qualitative studies could lay the groundwork for identifying commonalities and variables that would be measurable across different programs in different Latin American countries, contributing to an increased ability to use quantitative studies and to identify best practices for such groups.

This study does not address the use of medications. We recommend evaluation of the role of pharmacological treatment in community programs, because it appears to us that smaller doses are achievable for maintaining stability for those in community based programs versus hospital based programs.

### Global applications

While the FESEP program is designed for LMIC countries, many of the benefits identified may appear to be universal. Is the FESEP model something HIC countries can use? We don’t think so because the program is so heavily molded to the socio-political-economic reality of El Salvador, where there is no disability income, very high unemployment, no government community mental health programs, a frequent shortage of medications, very low educational levels, very high stigma, constant violence, and many other differences. One thing we can share is a common understanding that our struggle is global—users and family carers the world over share a common experience. We also share a need for the same basic qualities of life—health, work, respect, friends, opportunities, and love.

## Limitations

The greatest limitation of this study was the small sample size; the study could have been strengthened through a larger number of focus groups [62]. However, with 2 days of intensive work with a small group, we were able to achieve the benefit of a rich and thick description of the potential benefits of program participation across multiple social levels and for all principal stakeholders.

The potential for researcher bias was strong because the professionals in the focus group also served on the research team. This could potentially result in both biased researcher interpretation and biased data from FESEP program participants who might have felt they could only report positive outcomes. Still, for the purposes of this study, the researchers felt the benefits of this approach outweighed the potential negatives, including group learning across the different types of program participants, the synergistic thinking that would be generated, and what the researchers as professional participants could bring to the study’s findings.

Because one criterion was longterm duration of participation, the sample was likely to be composed of those FESEP program participants who were most helped and therefore most appreciative and most likely to talk positively about the program. This points to the need for further studies with larger samples, randomized designs, and outside interviewers.

None of the questions focused on negative outcomes or problems with the program. This appears to be a tendency in other qualitative mental health studies of self-help groups reviewed in this paper as well. Future studies should include questions about negative effects, shortcomings and problems with user and family self-help programs, groups and organizations.

The right to choose between being compliant or non-compliant with medication adherence and other doctor/hospital/intervention demands was not discussed in this study [63–65]. This human rights concept impacts how studies and programs view and measure indicators of wellness. Future studies should acknowledge this debate and account for non-compliance as potentially both a positive and negative indicator.

While we were able to comply with most criteria in the Consolidated Criteria for Reporting Qualitative Studies (COREQ) 32-item checklist [40], some criteria we were unable to meet. Data saturation was not possible due to the limitations of time and cost (#22). Transcripts were not returned to participants for comment and/or correction (#23), but participants did provide feedback on the findings (#28). Participant quotations were presented to illustrate the findings, but each quotation was not identified by a participant number (#29). With a small sample, any attempt to describe diverse cases would have been difficult (#32).

## Additional files

10.1186/s13033-016-0058-6 Consent to Participate in Research.
10.1186/s13033-016-0058-6 Questionnaire for Focus Group Study.
10.1186/s13033-016-0058-6 Transcripción de audios sobre investigación del programa, realizada los días viernes 22 y sábado 23 de marzo de 2013.
10.1186/s13033-016-0058-6 Focus group questions and summary of responses.

## Footnotes

1 The estimates for Central American countries are not based on solid information. In El Salvador, for example, Ministry of Health officials admit they have no idea how much is spent on mental health, since budget items are not categorized to capture that kind of information (personal communications with anonymous officials in El Salvador and Nicaragua in May and July, 2015).

2 In general, advantages of the focus group technique include ecological validity, the chance for participants to express data in their own words (an emic approach, in which data is collected with participants as the starting point) and to qualify their answers, the ability of the researcher to observe non-verbal communication and to follow-up for clarification, and group synergy and learning. Potential disadvantages include interaction effects, subjective interpretation and moderator bias [35].

3 Direct quotes appear in figures or within quotation marks in the text. We add footnotes on cultural context that help explain focus group responses for readers outside of the Salvadoran context.

4 This concept is akin to helping and accompanying one another along the journey, which the authors consider a type of “horizontal leadership.”

5 This is consistent with the lack financial resources in El Salvador and that three-quarters of the population is not covered by any health insurance program.

6 Civil society is related to three frameworks—associational life, the good society, and the public sphere [66]. In this study, civil society refers broadly to the nonprofit sector, whether formal or informal, legal or not legal. Laws regulating nonprofits are different in El Salvador, but the civil society sector is largely the same as the US However, many more groups do not have legal status due to the bureaucratic and financial difficulties of achieving that status. The benefit of legal status is the ability to participate in coalitions, whereas in the US the benefit is the ability to receive tax-deductible donations.

7 External stigma refers to society’s negative views and treatment of persons with mental illnesses, while internal stigma is the incorporation of those views by users into their own belief systems, causing them to benefit less from evidence based practices [45, 46]. Similarly, this may cause them to see themselves as people who do not have the capacity to be leaders.
