# Supplementary material for: A qualitative exploration of a family self-help mental health program in El Salvador
Source: Int J Ment Health Syst. 2016 Apr 1;10:26. doi: 10.1186/s13033-016-0058-6 (PMC4818454; doi:10.1186/s13033-016-0058-6)
Supplement: Supplementary file 1 — 10.1186/s13033-016-0058-6 Consent to Participate in Research. [file 13033_2016_58_MOESM1_ESM.docx]

# Consent to Participate in Research

# Identification of Investigators & Purpose of Study

You are being asked to participate in a research study conducted by Samuel Nickels and Dr. Susan Murphy from James Madison University. The purpose of this study is to obtain evidence regarding whether the community based intervention in El Salvador is effective at improving mental health wellbeing for families and consumers of mental health services. The intervention is the Family Education, Support and Empowerment Program (FESEP). This study will contribute to the researcher’s completion of Mr. Nickels’ doctoral studies in nonprofit leadership.

# Research Procedures

Should you decide to participate in this research study, you will be asked to sign this consent form once all your questions have been answered to your satisfaction. This study consists of an information and question sheet, followed by two focus group sessions, each two hours in length, that will be carried out at the offices of ACISAM. Researchers will be taking notes during the focus group sessions, and we will be audio recording the sessions. Your information will remain confidential and private to the researchers and those participating in the focus group sessions. An incentive will be provided to each participant of $10/visit to the ACISAM office, primarily to help reimburse transportation costs. These costs are coming from personal out-of-pocket of the researcher Sam Nickels, not from any JMU departmental funds.

# Time Required

Participation in this study will require a total of 6 hours of your time, not including transportation to and from ACISAM. This includes one hour for completing the information and question sheet at the ACISAM office, two hours for the first session, and two hours for the second session, plus one half hour for meal times after both sessions.

# Risks

The investigators perceive the following are possible risks arising from your involvement with this study: fear of loss of program access due to criticizing the program, confidentiality of personal information and comments, increased stress for persons with mental illness due to the length of the focus group and content information raised. While the intent of the research is not to induce emotional stress in any way, it may arise. We will reduce the potential for these risks through the following means: informing you fully of your rights, including your right to stop participating at any time you wish, and assuring participants they will not lose access to the program even if they criticize the program. We will also provide you with this informed consent, we will explain guidelines at the beginning of each session to help people ask for breaks or other assistance when stress levels rise, and we will have a psychiatrist and/or psychologist available to help monitor participant emotions or behaviors that reflect concern. The focus group sessions will be taped in order to provide researchers the ability to go back and clarify comments; taping will occur only if all participants give consent. At the end of the research process all recordings and notes will be destroyed in order to protect the confidentiality of participants. Any quotes or comments that are used in summaries of research results, publications and presentations will refer to anonymous persons or will use pseudonyms to protect the privacy of participants, and care will be taken to remove other potentially identifying information.

# Benefits

Potential benefits from participation in this study include participants having the opportunity to express their views about mental health issues, programs, services, organizations, and needs. Participants, as part of this research project, will contribute to a greater understanding of mental health wellbeing in El Salvador, as well as the benefits that can be obtained through programs such as the one they have participated in. Participants, including researchers, will learn from one another and we hope become more effective participants in the FESEP program. We also hope this work will lead to improvements in the current FESEP program and this program will become a model for other persons with interest in mental health around Central America. Finally, there is a small financial benefit of $10 per visit to ACISAM for participating in the study and to help cover transportation costs to and from the office. Meals or snacks will be provided at both focus group sessions.

# Confidentiality

The results of this research may be presented at conferences, as an article in a journal, or shared with faculty and students at James Madison University. A summary of the results will also be made available through ACISAM to any individual wishing to learn about the results of the study, including any and all participants in the focus group. The results of this project will be coded in such a way that participants’ identities will not be attached to the final form of this study. The researcher retains the right to use and publish non-identifiable data. While individual responses are confidential, aggregate data will be presented representing themes and generalizations about the responses as a whole. All data will be stored in a secure location accessible only to the researchers. Upon completion of the study, all information that matches up individual respondents with their answers, including audio recordings and notes, will be destroyed.

# Participation & Withdrawal

Your participation is entirely voluntary. You are free to choose not to participate. Should you choose to participate, you can withdraw at any time without consequences of any kind. Participants will be paid only upon completion of both focus group sessions.

# Questions about the Study

If you have questions or concerns during the time of your participation in this study, or after its completion or you would like to receive a copy of the final aggregate results of this study, please contact:

Samuel Nickels, Researcher Dr. Susan Murphy, Advisor

School of Strategic Leadership Studies Director, School of Strategic Leadership Studies

James Madison University James Madison University

[Nickelsv@dukes.jmu.edu](mailto:Nickelsv@dukes.jmu.edu) Telephone: (540) 568-7020

[Murph2se@jmu.edu](mailto:Murph2se@jmu.edu)

# Questions about Your Rights as a Research Subject

Dr. David Cockley

Chair, Institutional Review Board

James Madison University

(540) 568-2834

[cocklede@jmu.edu](mailto:cocklede@jmu.edu)

# Giving of Consent

I have read this consent form and I understand what is being requested of me as a participant in this study. I freely consent to participate. I have been given satisfactory answers to my questions. The investigator provided me with a copy of this form. I certify that I am at least 18 years of age.

I give consent to be audio taped during my interview. ________ (initials)

______________________________________

Name of Participant (Printed)

______________________________________ ______________

Name of Participant (Signed) Date

______________________________________ ______________

Name of Researcher (Signed) Date
