# Supplementary material for: A qualitative exploration of a family self-help mental health program in El Salvador
Source: Int J Ment Health Syst. 2016 Apr 1;10:26. doi: 10.1186/s13033-016-0058-6 (PMC4818454; doi:10.1186/s13033-016-0058-6)
Supplement: Supplementary file 2 — 10.1186/s13033-016-0058-6 Questionnaire for Focus Group Study. [file 13033_2016_58_MOESM2_ESM.docx]

**Questionnaire for Focus Group Study**

1. ***Defining participant characteristics***

Focus group participants will complete an information and question sheet ahead of time, which will be reviewed by the researcher in preparation for the focus group sessions. Assistance will be provided to persons with mental illness to complete the form. Information will include:

- Full name:
- Contact info (address, phone, email):
- Are you a family caregiver, a person with a mental illness (consumer), or both?
- What is your, or the person with illness in your family, diagnosis or diagnoses? (if known)
- Gender: male / female
- Educational level (last grade completed):
- For each family member who lives together with you, please list:
  - Place in family (father, mother, daughter, etc.):
  - Age:
  - Whether this person also has a mental illness and his/her diagnosis:
- Do the family caregivers live with the consumer?
- Number of hours weekly that you have paid employment:
- Number of hours weekly that you volunteer:
- Your average weekly income:
- The average weekly income of the consumer (if different from you):
- Your family’s average weekly income, if known:
- Your age:
- The age of the consumer (if different from you):
- Number of years that consumer has had his/her illness:
- How has the illness changed over that time period?
- Have those changes been related to the FESEP? How?
- Are there more than one person in the family with mental illness? How many? How has the FESEP been helpful or not helpful to a family with more than one consumer?
- Participation level questions
  - Which program components have you participated in?
    - How many years of participation in each component?
  - Which components have you taken on leadership responsibilities?
    - How many years have you been in a position of leadership?
  - Which program components if any your consumer family member participated in?
    - How many years of participation in each component?
    - Has the consumer taken leadership roles in the program?
  - What MH services/programs have you participated in outside of ACISAM/ AFAPDIM FESEP program?
  - What MH services/programs has your SMI family member participated in outside of ACISAM/AFAPDIM program?
- General effectiveness and satisfaction:
  - How effective is the program at improving mental health wellbeing for families and consumers of mental health services? (likert 1-5)
  - How satisfied are you with the program? (likert 1-5)
  - To what extent does the FESEP program provide you with a sense of community? (likert 1-5)
  - What other communities are you a part of:
- Trust:
  - In general, do you think other people can be trusted? (likert 1-5 scale)
  - Do you think ACISAM can be trusted?
  - Do you think AFAPDIM can be trusted?
  - Do you think CHHD can be trusted?
  - Do you think the National Psychiatric Hospital can be trusted?
  - Do you think private mental health providers can be trusted?
  - Do you think the Salvadoran government can be trusted?
  - Do you think the Salvadoran mental health system can be trusted?

1. ***Defining variables***

FOCUS GROUP SESSIONS: Qualitative questions that will be discussed in the focus group sessions are underlined below.

Orientation: introductions, guidelines, facilitators’ roles, defining terms for common understanding. Guidelines include confidentiality, use of focus group technique as a way to hear all perspectives and especially to hear what marginalized persons have to say, we encourage the expression of differing opinions (obtaining contrasting perspectives is a prime goal of focus group research), when a question is addressed to a particular group, eg consumers, that group will be asked to comment first and then we can hear from everyone else, there are no right or wrong answers, the sessions will be taped recorded and we will also be taking notes, you are free to end your participation at any time, if you feel uncomfortable please let us know so we can address you concern. Anticipate 2 sessions: Hypotheses 1 and 2 (9 questions) will be covered in the first session; hypothesis 3 (9+ questions) will be covered in the second session, depending on how quickly the session moves.

**Hypothesis 1: FESEP program participants have improved levels of mental health wellbeing (MHWB) compared to non-participants.**

- How would you define MHWB?
- What would be good ways to measure MHWB for a consumer? For a family caregivers?
- Facilitator will bring the following into the discussion if participants do not:
  - relapse rate,
  - socialization, self-esteem, sense of empowerment
  - psychological stress, ability to enjoy life, family stress
  - work, play, volunteerism
  - family finances, personal finances
  - relationships with family, relationships beyond family, friends, neighbors, MH professionals, community orgs, religious community
  - how you view your illness, how you view MH professionals, how you view yourself
  - family support to SMI person, family attitude toward SMI person
- Do you think that consumers and family caregivers who participate in the FESEP have better mental health wellbeing than those who do not? Why or why not?
- What are the biggest impacts of the program you have seen on others?
- Are there additional ways the FESEP program has impacted you?
  - Facilitator review Johnson (2005) & Lucksted (2008) reasons for FESEP programs.
  - Facilitator review consumer literature reasons consumers state for participation in programs/needs in HICs.
- What would be good ways to measure the impact of the FESEP program on individuals and families?

**Hypothesis 2: Mediating variables significantly impact MHWB.**

- Why do some family caregivers benefit more from the program than others?
  - Alternative question: Why does one family get involved while another doesn’t?
- Why do some consumers benefit more from the program than others?
  - Alternative question: Why do some consumers get involved while another does not?
- What other differences between families or situations might influence whether someone is helped by the program or not?

Facilitator will bring the following into the discussion if participants do not:

- - family participation in FESEP
  - level of family support
  - length of time of illness
  - level of compliance/denial
  - type and severity of illness

**Hypothesis 3: FESEP program participants and staff believe the program has positive impacts on their lives and the lives of others at the individual, family, community, organizational, and societal levels.**

- What is the importance of organizations like ACISAM and AFAPDIM?
- What are the goals of FESEP programs at the individual, family, organizational and national levels?
- Does the FESEP program meet these goals? Why or why not?
- Facilitator: service provision, advocacy, collaborative efforts, public education against stigma and about solutions, empowerment, leadership development, inclusion of persons with disabilities, research, other?
- How does the FESEP program compare with other services and programs available to consumers or their families in the community?
- What unique roles do organizations like ACISAM, AFAPDIM and CHHD play in helping consumers and family caregivers to achieve MHWB?
- How important is leadership in these organizations to achieve their goals and bring satisfaction to their members? Why?
- Do these organizations help develop leaders and leadership skills among participants? How? How could they do a better job in leadership development?
- Has the FESEP program helped to create greater social capital for individuals and organizations, both bonding and bridging capital?
- Facilitator: Discuss trust, efficiency, and satisfaction results from information sheets completed before the session, including how these might best be measured. Explain social capital theory: SC 🡪 performance 🡪 desired outcomes (eg, satisfaction with and perception of efficiency for organizational goals related to MHWB).
- How could we do a better job of creating social capital?
- Facilitator: Regional network for MH would help create social capital?
