# Supplementary material for: A qualitative exploration of a family self-help mental health program in El Salvador
Source: Int J Ment Health Syst. 2016 Apr 1;10:26. doi: 10.1186/s13033-016-0058-6 (PMC4818454; doi:10.1186/s13033-016-0058-6)
Supplement: Supplementary file 3 — 10.1186/s13033-016-0058-6 Transcripción de audios sobre investigación del programa, realizada los días viernes 22 y sábado 23 de marzo de 2013. [file 13033_2016_58_MOESM3_ESM.docx]

**Transcripción de audios sobre investigación del programa, realizada los días viernes 22 y sábado 23 de marzo de 2013.**

**Audio 800278**

1: primeramente quiero agradecer a ustedes por venir y dar a este proyecto su tiempo, todos entienden que estamos llevando el estudio del programa y también que queremos sus pensamientos sobre varias preguntas.

La primera cosa que yo quería hacer era darles la bienvenida a todos y discutir un poco la agenda: primeramente voy a dar algunas pautas o guías o directrices para que todos entendamos las reglas e información importante, y después de esto vamos a responder a diferentes preguntas y luego tendremos la cena.

Algunas de las directrices son:

1. Lo que es dicho en este grupo se queda en grupo. Es decir, tenemos que tener respeto de confidencialidad, no quiero salir y decir a “D” “mira “7” dijo….” Queremos respetar totalmente la confidencialidad de lo que dicen todos.
2. Recesos y otras necesidades, si está cansada, si no puede enfocarse más, si necesita un receso para ir al baño o cualquier cosa pueden notificarnos y decirnos esto y lo hacemos.

Soy el facilitador hoy, pero me ayuda “2” y también “3”, cuando llegue.

1. A parte de esto también todos somos iguales en este proceso.

Entonces, la idea de este grupo focal es que hay preguntas sobre estos programas que son importantes, pero no es importante solamente la perspectiva del profesional o de mí como investigador, sino que también las perspectivas de los familiares y los usuarios, por eso tenemos este tipo de grupo hoy;

1. Usted tiene derecho de salir en cualquier momento del estudio, tiene derecho de criticar el programa o decir cosas negativas sin pérdida de su participación en el programa.
2. No hay respuestas buenas ni malas puede decir lo que piensa.

Estamos usando esta técnica del grupo focal porque es una manera en que todos pueden escuchar los puntos de todos los demás especialmente de la gente más marginada. También es para fomentar la expresión de opiniones diferentes, y esto es importante porque lo que queremos no solamente es la misma cosa de cada persona, estamos muy interesados en si alguien tiene una opinión diferente, perspectiva diferente, información diferente o experiencia diferente puede usted compartirla también.

Cuando una pregunta se dirige a un grupo particular, por ejemplo si es una pregunta diferente, particularmente desde la perspectiva de los usuarios ese grupo se le pidió que comentara primero y entonces podemos saber de todos los demás, entonces preguntamos a usuarios primero o a familiares y ellos responden y después los demás pueden responder.

1. Las sesiones serán grabadas y también vamos a tomar notas.
2. Si te sientes incómodo (mal) por favor háznoslo saber para que podamos atender la preocupación.
3. Y por último, estamos pensando en dos horas y medio pero con varios recesos.

Antes de la primera pregunta, quiero saber si ustedes ¿tienen preguntas sobre el proceso o la agenda de hoy? ¿No? Comenzamos entonces.

La primera pregunta es ¿Cómo definirías el bienestar de salud mental? cualquier persona puede empezar.

- 4: el bienestar de salud mental es estar uno no enfermo y tener una mente sana, cumpliendo las responsabilidades que como individuo uno tiene.
- 6: cumplir actividades
- 1: Cuando dice cumplir las actividades ¿es tener la capacidad para hacer esto o solamente para cumplirlas?
- 6: tener capacidad para cumplirlas y saber cumplir-
- 5: para mí el bienestar para salud mental es lo contrario de cuando uno está en crisis, porque cuando uno se encuentra en crisis o tiene algún defecto, uno vive una vida muy sufrida; mientras que con el bienestar de salud mental uno puede tener una vida en felicidad y tener lo que todos tienen, estar libres, no estar encerrado, no es necesario que la familia lo tenga a uno controlado sino que uno puede disponer de su tiempo y puede también hacer las cosas cotidianas de la vida: trabajar, tener esposa y también la familia está con uno congratulada, no hay problema entre padre e hijo, entre hermanos, sino que se vive en armonía.
- 1: Dijo no controlado por la familia ¿qué significa esto?
- 5: o sea que la familia [no] lo vigila a uno lo que anda haciendo, y tal vez a uno eso le molesta, si uno sale a alguna parte la familia está pendiente de eso y pueden preguntar y “¿viste a “5”? ¿Lo viste en tal lugar? ¿Y qué estaba haciendo?” y (con bienestar) confían en uno, ya no piden información de terceras personas-.
- 4: también significaría tener una actividad productiva que genere algún ingreso para uno mismo o la familia y no tener ningún vicio que afecte la salud física o mental de una persona.
- 1: Una sugerencia, cuando haya una pregunta permitir que todos hablen para hablar por lo menos una vez y después seguir una segunda o tercera vez si queremos.
- 9: en mi opinión pienso de que la enfermedad siempre está ahí, ya se sabe que es una enfermedad que tiene que estar controlada, y para mí bienestar es que a ellos (usuarios) no les faltara el medicamento, eso fuera un bienestar y que ellos tengan esa mejor vida, una claridad (lucidez) para salir a divertirse, tener un rato ameno con la familia, sin tener que estar pendiente de ellos (diciéndoles) “pórtate así, pórtate allá”, sino que ellos solos se coordinen, porque en mi caso yo le digo a Jorgito: “mire hijo compórtese así, compórtese de otro modo”. Quizás a ese control se refería “5”. Pero eso es así, para mí eso sería: que no les falte el medicamento, tener una buena comunicación con ellos, que por lo bien que se sienten con el medicamento y el ambiente en que están con la familia se siente uno bien, aunque sabe uno que el mal está ahí, que si se descuida puede entrar en crisis otra vez.
- 8: en mi caso lo definiría como el estado de toda persona de gozar a plenitud de nuestras capacidades cognitivas, emocionales, al mismo tiempo como el derecho que tenemos todas las personas de disfrutar de un ambiente, y este es un ambiente general, no solo algo en particular, donde la persona pueda….ya perdí el hilo (carcajadas del grupo)….bueno hablaba de un ambiente, pero no solo es un ambiente como lo verde que conocemos, sino que un ambiente general como la familia, una sociedad donde se respete los estados psíquicos de cada una de las personas.
- 7: yo comparto la opinión de “8” al decir que es el estado donde la persona puede expresar sus cualidades, sus capacidades al máximo, sin embargo pienso también, que el bienestar de salud mental no solo es ausencia de enfermedad, sino también a partir de esta condición de una enfermedad mental, poder gozar de respeto y también de ejercer el derecho de la persona desde su condición y que esto le permita desenvolverse desde ese contexto como persona y como todo lo que es.
- 1: bueno voy a hablar yo, que soy participante también (risas). Para mí bienestar es como calidad de vida, entonces son todas las cosas que contribuyen en cada personas en su calidad de vida, y por cada persona puede ser diferente, pero en general incluye muchas cosas, quizás uno de los más importantes es la habilidad, la capacidad de relacionarse. Yo pienso en mi hermano, por muchos años no podía relacionarse con ninguna persona, y todavía tiene un poco de trabajo para relacionarse pero no es como antes, es mucho mejor ahora, y él perdió su sanidad cuando tenía 15 años era solo un adolescente, y la adolescencia es un periodo muy importante para la gente para desarrollarse entre niñez y adulto y él perdió esta parte de su vida, no podía desarrollarse a sí mismo, pero aparte de relacionarse no tener discriminación, tener trabajo, tener sus necesidades como comida, casa, por lo menos algo de dinero para vivir una vida sencilla, por lo menos, y quizá la última cosa es el amor, y el amor creo que viene cuando uno está feliz pero más que todo tiene relaciones buenas con la familia y con los demás. ¿Alguién más quiere comentar otra vez?
- 4: sí, yo quería comentar de que ayer mi papá me decía que en EEUU unos psiquiatras tenían un estudio y decían que las personas que tienen un vicio y lo han dejado han demostrado que son sanas mentalmente, pero las personas que tienen un vicio ahí demuestran que no tienen salud mental, entonces yo le dije a mi papá “a pues yo tengo que demostrar que yo no tengo problemas de salud mental porque puedo dejar el tabaco” y le decía que podía demostrarle que no tengo problemas de ningún tipo de salud mental dejando el tabaco, porque todo aquello que atente contra la salud física y mental de una persona es lo que hace daño y una persona pasa de la salud a la enfermedad, entonces también parte de la salud mental es ser feliz, como dicen mente sano, cuerpo sano, pero una persona feliz o contenta y feliz por las cosas que se le permite vivir o por las cosas que uno tiene, es también parte de la salud mental.
- 6: para mí ser esquizofrénica no es tan duro, pero es por la medicina, porque a veces se haya y a veces no. El problema es que cuando uno vive con esquizofrenia todo le dicen que está loco, pero a mí eso no es una enfermedad sino que es parte de una enfermedad común y corriente.
- 1: ¿Entonces parte de su pensamiento sobre que bienestar de salud mental es que una persona entienda que la enfermedad es de por vida y puedan ajustarse, acostumbrarse a esta enfermedad? ¿Está diciendo esto?
- 6: sí, exactamente. Solo hay que aprender a vivir con la enfermedad.
- 4: una vez vi una película que se llama “El secreto”…se llama así porque el secreto que dan ellos es darle gracias a Dios por las cosas que uno tiene y no por las cosas que uno no tiene, y así va a ser feliz y agradecido con Dios.
- 5: también bienestar de salud mental es que no tiene que haber represión contra uno porque cuando uno está enfermo a lo primero que uno tiene miedo es a la policía, y tiene muchos temores también, porque la gente por ejemplo la casa de uno la tildan como “la casa del loquito” y tal vez está haciendo algún trabajo uno y los vecinos están al tanto de lo que uno está haciendo, entonces una persona vecina puede denunciarlo a uno por algo que no está haciendo, o sea que también uno se expone a ser calumniado [SLANDERED] y cuando uno está bien eso no sucede porque el que anda en el bien a nada teme, pero si siento yo y tengo mucho temor que los vecinos se quejan de uno, pero si puede uno tener amigos, ir a la iglesia y llevar la fiesta en paz.
- 3: desde mi punto de vista bienestar de salud mental es tener una libertad de expresión independientemente del lugar donde usted se encuentre, aparte de eso tener buenas relaciones interpersonales, tener comprensión, algo muy importante pienso que es tratar de sobrellevar la situación ante factores estresantes, saber cómo salir de un problema sin dañar a otros, y quizá por último tratar de ser muy empático con la gente con la que usted se interrelaciona, acuérdese que tenemos diferentes ámbitos: laboral, de casa, de comunidad. Tratar de ser empático en todos los roles de la vida, y quizá por último es tratar de disfrutar cada año que vivimos, o sea, cuando me tocó ser adolescente fui feliz siéndolo, hoy voy para mis 50 años y tratar de gozarlos y vivir a plenitud.
- 2: yo estoy de acuerdo con lo que el grupo ha planteado, pero tengo dos ideas aunado a esto: una es aceptarse a sí mismo y la otra que es un poco revolucionaria es transformarse y transformar, es decir, salud mental es la capacidad de transformarse y transformar.

- 1: explique más estas dos cosas por favor.

- 2: todas las personas cambiamos constantemente, somos personas que todos los días cambiamos, entonces, bienestar de salud mental es cambiarse cada día, ser mejor cada día y también implica o es cambiar las cosas que tiene uno alrededor, entonces en la medida en que uno cambia, cambian las cosas que están alrededor, en la medida de que las cosas que están alrededor uno también cambia, entonces es una relación dialéctica de cambiar y transformarse. Yo me transformo en la sociedad, para mí eso es importante en el caso de la salud mental, capacidad de transformarse, ser mejor y capacidad de transformar a la sociedad.

DIFERENCIA ENTRE **BIENESTAR de SALUD MENTAL y SALUD MENTAL** (pregunta de clarificacion)

- 1: y qué piensan ustedes ¿bienestar de salud mental es lo mismo con salud mental o las dos frases tienen diferente significado?

6: para mí son distintos.

- 1: ¿cómo?

- 6: son muy distintos porque salud mental es como decir que uno está bien o está mal, [COMO MEDICAMENTO, REFIERE A LA ENFERMEDAD, PERO ] bienestar significa estar en una forma tranquila, positiva y relajada-.

- 8: yo quisiera decir algo más y es sobre el estigma, con todo lo que hemos expresado acá, también bienestar de salud mental implicaría eso, que vivamos en una sociedad también libre de todo estigma, donde no juzguemos a las personas por su modo de vida o por su modo de pensar si no sabemos que es lo que pasa a cabalidad qué es lo que pasa en cada una de nuestras, eso nada más.
- 5: para mí salud mental es el medio donde se llega al bienestar o sea que si nosotros tenemos una salud mental medianamente buena, vamos a tener un bienestar producido por esa salud mental, porque cuando yo andaba en crisis no me daba cuenta de que en mi cabeza estaba funcionando algo mal porque al principio reprimía [REPRESSED] todo, pero después me solté y anduve en la calle, y yo miraba que eso era lo “in”, lo máximo, que yo salía a la calle y no le tenía miedo a nada ni a nadie ni me importaba lo que iban a decir de mi los demás y tampoco me ponía pensar que la gente miraba mal eso y para mí, en la familia todavía no había entrado esa palabra “locura”, yo estaba saliendo (de la adolescencia) a ser adulto, pero ahora que estoy con medicamentos yo digo “púchica” (frase que expresa admiración sobre algo: qué barbaridad, no lo puedo creer), “si anantes (por poco) no morí en esas locuras que anduve haciendo”. Entonces el medio por el que tengo este bienestar es porque medianamente tengo salud mental, que quiero colarme [SNEAK] entre los que tienen la salud mental buena y relativamente estable pero si siento la diferencia, no puedo decir que tengo todos los beneficios de la salud mental, pero si comparado como cuando yo estaba en crisis hay una gran diferencia,[SO IT’S RELATIVE, HE’S NOT SAYING HE HAS STABLE GOOD MENTAL HEALTH, BUT COMPARED TO HIS PROBLEMS DURING CIRIS THERE IS A HUGE DIFFERENCE] y en verdad cuando yo siento que voy a recaer me da miedo y me desespero, pero luego voy viendo como encamino mi situación y digo “tal vez esta medicina me ayuda más o me tomo otro poquito, o si voy donde el doctor y le digo mire doctor estoy sintiéndome mal, entonces voy buscando salida al problema, pero si pienso que la salud mental es el medio para llegar al bienestar de la salud mental para poder uno funcionar pues en la sociedad.
- 1: bien, te voy a parar, y quiero preguntar a otra persona si puede dar un resumen de esto porque es un dicho muy largo y yo quiero entender mejor el resumen, pero necesito a otra persona para darme el resumen de lo que él acaba de decir.

4: yo le puedo decir.

-ok-

- 4: él ahorita está en una salud mental media y quiere tener una salud mental completa para estar en una salud mental bien, funcionando bien, y que el bienestar de la salud mental él se dio cuenta de que estuvo haciendo cosas muy peligrosas que hasta pudo haber perdido la vida, pero que ahora con este programa de ACISAM y todo esto uno se va encarrilando a conseguir una salud mental.

A parte de todo esto, definiría el bienestar de salud mental, con todas ideas que hemos dado podemos ver que todo eso contribuye a las cosas que hemos platicado y cada uno ha dicho, contribuye a la salud mental.

- 1: muy bien. Disculpas por mi idioma, pero todavía no estoy bien claro de qué es lo que estaba diciendo “5” sobre la diferencia para él entre salud mental y el bienestar de salud mental.

8: -yo creo que dijo que la salud mental es lo que conducía al bienestar.

- 1: ¿cómo?
- 8: “5” dijo que salud mental era el paso o lo que conduce para obtener el bienestar, o sea que yo lo que le entiendo es no habrá [BIENESTAR] salud mental sino tenemos salud mental estable.
- 1: ¿entonces bienestar es como un gran concepto de sano y tener todas estas cosas, y salud mental es como todas las etapas de actividades y proceso de entrar o llegar hasta bienestar de salud mental?
- 5: si, así es.
- 1: ¿es un buen resumen?
- Sí.
- 1: Bien. ¿Alguién más quiere comentar para usted la diferencia entre el bienestar y bienestar de salud mental? Cualquier persona.
- 4: [THIS PART LEFT OUT OF TRANSCRIPTION]: Hay otro punto, olvidado, que es por ej, lo que puede ayudar una persona es la parte espiritual, como assistir a una iglesai Cristiana, habla de Dios, como vivir una vida guardando mandamientos, los leyes naturales, de Dios, si uno quebranta casi siempre es negativa condequencias (carcel, psiquiatra, o al cementario). Entonces no es solamente la parte medicamento, pero la parte espiritual que ensena en las iglesias, no importa Catolico o Protestante.
- 7: yo quiero decir algo, desde mi percepción creo que salud mental es un término mucho más amplio que bienestar de salud mental porque salud mental puede definir ese estado de bienestar o ese estado de no bienestar de la salud mental, y que se engloba en todo el contexto en que vivimos, tanto a nivel individual como social, en todos los niveles: en lo económico, en lo material, en lo espiritual, en lo emocional, es todo, es un concepto mucho más amplio.
- 1: ¿bienestar o salud mental?
- 7: Salud mental. Bienestar es parte de la salud mental.
- 1: ¿cuál parte es?
- Es la parte de lo bueno, porque también salud mental puede ser una salud mala o una salud que no esté en bienestar.
- 3: yo quizá opino un tanto diferente, para mí la salud mental seria como la autoestima, la autodeterminación, la auto realización, la capacidad de distinguir entre lo bueno y lo malo, y el bienestar, considero yo, que es cuando todo tú has cumplido todo eso, entonces, puedes tener una salud mental buena, mala, mediana o baja, mientras que el bienestar consideraría yo que es lo máximo es cuando yo me realice en todas la etapas de mi vida, en todos los aspectos, en todos mis roles. Entonces voy a lo contrario de “7”, pues yo considero que salud mental con como los hitos [MILESTONES] y el bienestar es cuando los he cumplido todos-.
- 8: quizá (voy) en esa misma línea de la doctora, salud metal sería como lo particular, y el bienestar sería como lo general.
- 1: ¿bienestar es lo general?
- Sí.
- 1: ¿está de acuerdo con ella entonces?
- 8: Sí y desde un enfoque de derecho, como yo estudie leyes, recuerdo que cuando nosotros mirábamos en derecho social y empezamos a ver todas las garantías que se nos tendrían que prestar a todas las personas, bienestar lo ubicábamos como lo óptimo o el estado pleno de cada una de las personas-.
- 1: Bueno, soy participante y voy a decir mi opinión, para mí los dos son los mismo, salud mental con bienestar de salud mental. Solamente. Pero estoy de acuerdo con todo lo que están diciendo todos, la razón por la que estoy haciendo esta pregunta es solamente para clarificar en qué estamos pensando cuando hablamos de esta frase de bienestar de salud mental, en mi mente es lo mismo que salud mental, pero quizá para otras personas no es lo mismo, entonces es interesante, pero también es muy interesante que algunos piensen que bienestar es más amplio, como la meta, el fin; pero otros piensan que salud mental es el fin y bienestar es como un parte de esta. Pero en general tenemos muchos vínculos y entendimiento en común. “2” ¿quiere comentar?
- 2: no.
- 1: Está bien. Vamos a tomar un receso de cinco minutos, podemos estrecharnos y hablar con nuestras mamás como queramos pero solamente cinco minutos.

**Inicia audio** **800279**

- 1: Bueno empezamos otra vez, yo estaba hablando con “2” sobre algunas cosas, y le pedí a él que compartiera lo que estaba diciendo porque es uno de los participantes, aunque es un profesional y facilitador, siempre quiere dejar adentro sus propios pensamientos, pero este es un espacio para oír de todos los diferentes actores en este proceso, entonces vamos a escuchar a “2” y después seguir con la próxima parte de esta pregunta.
- 2: es que a mí me llamó la atención mucho el planteamiento que hizo “1” sobre qué es salud mental, porque habíamos planteado bienestar de salud mental como un sinónimo de salud mental en la investigación para este propósito, en ese sentido me despertó mucho la curiosidad de compartir un poco el concepto que maneja la OMS sobre salud mental el cual es “un estado de completo bienestar”, sin embargo el concepto de la OMS sobre salud mental es muy positivista, o sea es muy “solo lo bueno”, desde la perspectiva de ACISAM, “salud mental no solamente es un estado de completo bienestar ni solamente es ausencia de algo o de una enfermedad, es como un proceso de activación”, entonces, entendido desde esa perspectiva, salud mental es activarnos para transformar algo y no solo se entiende como una plena satisfacción, eso estaba compartiendo con “1”, y creo que para ejercicio de la investigación habíamos entendido que bienestar de salud mental era igual que salud mental, entonces agregar esa idea y también una idea que está muy vinculada a lo que entendemos por salud mental que tiene que ver con la justicia, realmente aquí en el país cuesta hablar de justicia, entonces también es una experiencia que nos tiene que evocar el derecho a la justicia.
- 1: Bien, muchas gracias “2”. La próxima parte son algunas preguntas relacionadas a este concepto de bienestar, aquí tengo algunos conceptos que los investigadores han estudiado sobre el bienestar de salud mental, y yo quería la opinión de ustedes sobre estar partes de salud mental, por ejemplo, una cosa de la que hablan mucho es la tasa de recaída, entonces por ejemplo, uno tiene bienestar o más bienestar si su tasa de recaídas está bajando poco a poco, si uno toma medicamentos no tiene que ir al hospital y está bajando su tasa de recaídas, esto es una de las cosas que los investigadores les gusta medir, entonces, otras cosas que los investigadores les gusta medir son: autoestima, sentido de empoderamiento, socialización, calidad para disfrutar la vida, trabajo, etc.

Entonces ustedes pueden hacer comentarios sobre estas cosas también y vamos a pasar uno por uno, entonces ¿piensan que **las tasas de recaídas o el número de veces que tienen que ir al hospital es una buena manera de medir el bienestar de salud mental**? ¿O piensan que no? ¿O qué quieren comentar?

- 9: yo entiendo ahí en la tasa de recaídas que desde hace dos años para acá han sido menos las recaídas de Jorge y de Marvin (hijos), porque en el 2010 casi todo el año estuvo ingresado él, lo ingresaban, me lo daban, ingresaba y así, y en diciembre me le dieron el alta con el medicamento que es el que actualmente está tomando, y desde ahí solo voy a consulta cada tres meses, y le dejan el medicamento cada tres meses, si hay yo lo recojo, sino pues yo siempre voy la fecha que está en la receta, pero ha bajado y yo me siento mejor.

- 1: ¿entonces han bajado las recaídas?

- sí, han bajado.

- 1: ¿entonces ésto implica para usted que es un aumento en el bienestar de sus hijos y su familia? entonces están relacionados: uno baja y otro aumenta.

- Sí.
- 6: yo en mi recaídas subía y bajaba y así iba, cuando estaba con la primera medicina no me caía bien…..pero ME CAMBIARAN, con la otra medicina mejoré…
- 1: una pregunta “6” ¿para usted esta idea de tener menos y menos entradas en el hospital, significa para ti bienestar de salud mental o los números de entrada al hospital no importa como medida de bienestar?
- ....nunca he estado ingresada….
- 1: ¿solamente ha tenido tratamiento privado?
- Sí, exacto.
- 1: **Voy a cambiar la forma de la pregunta ¿ustedes piensan que la tasa de recaídas está relacionada o es importante a la medida de bienestar o no**? Entonces solamente dicen sí o no. ¿todos entienden bien qué es una tasa de recaída?
- (Todos asienten que sí).
- 1: Comenzaré con usted y solamente dígame, ¿está relacionado o no?
- 7: sí está relacionado.
- 8: igual, está relacionado.
- 9: así es, está relacionado.
- Pueden decir no si quieren.
- 3: - a menos recaídas, más bienestar-.
- 6: sí.
- 4: sí.
- Yo también sí.
- 5: yo digo que no.
- 1: ¿por qué?
- 5: porque a aquí en nuestro país los doctores dejan la cita para tres meses, cuatro o cinco meses, y tal vez lo que el paciente necesita es nada más que le suban la dosis del medicamento en el momento exacto, pero si tiene la cita hasta dentro de cinco meses ya se ha profundizado la enfermedad, porque para ir a pasar consulta de emergencia le atiende un doctor de residente (general), si tiene suerte lo ve un psiquiatra, pero si a usted le dan el medicamento al tiempo exacto no tiene que ingresar….
- 1: ¿entonces su problema no es con la idea de las recaídas, sino más con el sistema, tiempo de tratamiento, tiempo para conseguir una cita y todo esto?
- 5: Sí, que el doctor esté a tiempo cuando uno lo necesita.
- 1: Bueno, estos otros: autoestima, sentido de empoderamiento, socialización, las leeré todas y díganme si, ¿**todos entienden sentido de empoderamiento**?
- 4: -no, yo no-.
- 1: ¿puede explicar? (le pregunta a “3”, psiquiatra)
- 3: es cuando usted tiene la capacidad de identificar tu padecimiento y hasta cierto punto comprenderlo, como cuando dijo “5” “yo sé cuándo voy a recaer”, o sea, él ha empoderado de su padecimiento y empieza a identificar los síntomas, los signos, o incluso él sabe cuando no hay medicamento, como dijo Karlita “cuando no tomo el medicamento hay recaída”, a medida tú te vas empoderando de tu salud mental….
- 4: ¿Cómo estar consciente de que uno padece una enfermedad mental y saber el momento en que uno está más grave?
- Exacto.
- 1: creo que esto es empoderamiento en un nivel más personal, también estaba hablando “2” de empoderamiento pero más en el nivel de transformación. Y para mí, empoderamiento es para dar poder a una persona para que ellos puedan entender la enfermedad, el estigma, sus derechos de luchar por una sociedad más justa, todo eso más o menos es empoderamiento.

**¿Todos entienden “socialización”?**

- 6: no, no sé qué es “socialización”.
- 1: “2”, ¿quiere explicar?
- 2: bueno, de forma sencilla podríamos decir que es cuando uno se relaciona con las demás personas, uno se socializa, se relaciona, esa es una forma bien sencilla de entender lo que es socialización y también se entiende que es un proceso en donde uno va aprendiendo las diferentes normas y reglas de una sociedad, básicamente dos ideas bien concretas.
- 1: otra idea es cuando uno está negando la enfermedad y no toma su medicamento y todavía tiene psicosis o muchos problemas, no se puede relacionar con otras personas, pero bajo tratamiento uno se siente mejor, se puede relacionar con la familiar y otras personas, esto es socialización también.

OK, ¿**capacidad de disfrutar la vida, trabajo, jugar, voluntariado** ¿cómo es ésto?, ¿tener la capacidad de ser un voluntario?

- 2: sí, realizar un trabajo por voluntad, es decir sin recibir pago alguno, entonces esa es una manera de estar activo, de participar, de aportar de lo que nosotros tenemos, como nuestro trabajo, por ejemplo, y es una manera de servir.
- 1: OK **¿estrés?** Todos nosotros entendemos esta palabra desafortunadamente, el estrés psicológico es como más personal que tenemos en nuestra mente, estrés familiar son los conflictos y problemas en las familias.

**“Finanzas de la familia”, “finanzas personales”, “relaciones con la familia”** Es como la socialización. **“Relaciones más allá de la familia”, “formas de ver la enfermedad”** Es decir, uno puede tener diferentes formas de ver la enfermedad y uno puede acostumbrarse a manejar su propia enfermedad. La próxima es la **“forma de ver a los profesionales de salud”** entonces una persona puede ver al profesional como el enemigo o puede ser como amigo, o entre éstos dos, ni bueno ni malo. **“Cómo usted se ve asimismo” es como autoestima**, la perspectiva de sí mismo, aunque tenga una enfermedad soy una buena persona o mi enfermedad me hace malo, es cómo se ve usted.

**“Apoyar al usuario/a”** esto es importante para entender, esto significa si la familia da apoyo o no, si uno se siente soportado o no, es cómo actúa la familia hacia la persona que tiene la enfermedad. Y por último **“cómo actúa el familiar cuidador hacia el usuario/a”** ésto es casi lo mismo que el apoyo familiar pero enfocado específicamente el cuidador.

Entonces yo quiero que ustedes tengan unos minutos para que piensen cuáles son los 2 ó 3 más importantes de estos para dar un indicador de bienestar para la persona. ¿Entienden la pregunta?

- 2: -no, te iba a preguntar si la podes hacer otra vez-.
- 1: OK. Quieren ustedes pensar por unos minutos cuáles de éstos son más importantes para una medida de salud mental. ¿está claro?
- SÍ.
- OK
- 3: - “1”, quizá para poner un límite o una diferencia, la perspectiva mía como profesional sería una y como personal sería otra, porque podría decir que bienestar sería reducir las tasas de recaídas, tener sociabilidad, tener el apoyo familiar. Pero a manera personal, quizá me llenaría por la capacidad de disfrutar la vida, buenas relaciones familiares, y en lo particular a mí me llena mucho el voluntariado.
- 1: Y dice todo esto porque quiere votar dos veces (a manera de broma)
- 3: No, porque tengo dos perspectivas diferentes, mi perspectiva hacia el usuario y la mía personal, entonces en ese sentido yo diría con el usuario sería que tenga menos recaídas, mejores relaciones familiares, pero las mías ya quitándome el profesionalismo sería la capacidad de disfrutar la vida, tener buenas relaciones familiares y el voluntariado.
- 1: ¿todos han tenido tiempo para pensar en dos, tres o cuatro cosas más importantes para ustedes?
- 3: (a “2”) si quiere vamos poniendo rayitas: voluntariado, las relaciones interfamiliares, la capacidad de disfrutar la vida y relaciones con la familia. Y por profesional es “disminución de las tasas de recaídas”, “la socialización y el apoyo familiar”.
- 4: ¿puedo hablar?
- 1: Sí.
- 4: Yo quería decir que todas son importantes, pero voy a elegir dos: “las tasas de recaídas” y “cómo usted se ve a sí mismo” porque a mí me cuesta mucho aceptar que tengo un problema de salud mental, porque en mi mente yo estoy lúcido y cuerdo, pero uno de mis problemas ha sido el tabaquismo, no me he podido dominar ni dejar el tabaquismo entonces siento que recaigo y a veces le hecho tanta culpa al tabaquismo “que por eso no he encontrado un empleo”.
- 1: Ok. Vamos a seguir un poco rápido con esta pregunta, entonces ¿tienes dos diferentes prioridades para ti?
- 9: - “apoyo familiar hacia el usuario, tasa de recaídas, cómo usted se ve a sí mismo”
- 7: “autoestima, las relaciones más allá de la familia”
- 6: “trabajo”
- 4: -hay un gran desempleo “6”-
- 6: si, por eso. Y “estrés psicológico”
- 5: “el apoyo familiar, la socialización y el estrés psicológico”
- 1: para mi “tasas de recaídas, socialización”
- 8: “autoestima, las relaciones más allá de la familia y apoyo familiar hacia el usuario”
- 2: “sentido de empoderamiento, autoestima y finanzas personales”
- 4: “2”, ¿podría agregarme ahí “las relaciones con la familia?
- 2: sí, claro.

**QUESTION 2**

- 1: vamos a seguir entonces hasta la pregunta número dos, pero creo que esta primera pregunta era para discutir mucho, estas otras preguntas no tanto. entonces, la segunda pregunta es: **¿crees que los usuarios y los cuidadores que participan en nuestro programa tienen mejor bienestar de salud mental que aquellos que no participan?**
- 4: sí.
- La pregunta es “sí” o “no”
- 6: sí
- 3: sí
- 8: sí
- 7: sí
- 5: sí
- 1: no estoy seguro porque no he medido estas cosas, pero quizá si.
- 2: sí
- 1: La próxima pregunta es por qué, ¿por qué cree usted que los que participan en el programa tienen mejor bienestar que los que no participan? This is NOT the question?!?!
- 9: Porque ellos ahí están con la mente ocupada, se socializan con todos los compañeros, ese espacio para ellos es para que se distraigan y no estar con la mente encerrada en lo mismo.
- 4: canaliza la energía en algo productivo.
- 6: ser más productivo.
- 5: porque tiene uno sentido de pertenencia y está haciendo algo bueno y que está trabajando para tener mejor salud.
- 3: yo dije “sí” porque considero que dentro del grupo hay mucha comprensión y porque se mantienen ocupados en caso del usuario.
- 1: para mí, yo sé que la gente que participa están afuera de casa, no encerrado.
- 8: en mi caso, el programa ha sido más benefactor para mí y bastante rechazado por mi hermana, y hasta el momento no entiendo porque, porque yo veo a los demás usuarios que fácilmente se adaptan, pero en el caso de “A”, “2” lo sabe, la doctora también, es una de las usuarias que se ha resignado a ser partícipe del programa, entonces ahí sería una de las variables.
- 7: yo dije que sí porque hay aceptación, desde el conocimiento que tengo con el grupo, y respecto a lo de Amanda para mí, gran parte de su renuencia a participar en el Programa es porque ella todavía no acepta que tiene una enfermedad.
- 1: ¿Cuándo dijo aceptación antes, era hablando de qué?
- 7: de pertenencia.
- 1: ¿alguien más quiere decir por qué?
- 4: bueno, realmente el asistir a un grupo, llámese a una iglesia o a una actividad deportiva, o a una reunión, el asistir es muy importante, así como cuando uno asiste a clases, es importante asistir porque ayuda y aparte de eso, tratar la forma de no desperdiciar el tiempo.
- 1: ¿entonces está diciendo que lo mejor es que uno asista?
- 4: si o sea que sí es importante asistir, porque ahí se ve que uno está aprovechando el tiempo y que a uno le interesa algo.
- 5: a mí me da bienestar porque recibo dinero, dice mi hermana que “grano a grano se llena el buche (el estómago) la gallina” y si yo voy recibiendo un poquito por aquí y otro poquito por allá, entonces puedo sostenerme económicamente.
- 6: para mi asociarme significa asociarme con los demás, salir, ser más amable, más gentil respecto a lo que era antes….me gusta relacionarme con mi familia…
- 1: una pregunta para ti ¿si no hubiera participado en el programa, hubiera sido diferente tu vida?
- 6: sí
- 1: ¿cómo?
- 6: solo pasaría brava, enojada, tensa por todas las cosas, sería no amable
- 1: OK. Tenemos la pregunta número dos “b” ¿cuáles son los cambios más significativos que usted ha visto en los demás participantes del programa? No estamos hablando de sí mismo, sino que de los demás en el programa.
- 7: yo tengo una pregunta, ¿participantes familiares, usuarios o en general?
- 1: disculpa, buena pregunta. Realmente todo, en la primera parte estábamos hablando mucho de nosotros mismos, pero ahora, hablaremos de lo que ustedes han visto en otros usuarios u otros familiares o podría ser en los profesionales también, ¡no sé si “2” o “3” han cambiado en este tiempo que tienen en el programa o no!
- 4: y se ve una mejoría el salir a pasear
- 5: ¿vamos a hacer una opinión de los usuarios?
- 1: o de los demás, profesionales, familiares, lo que usted ha visto en ellos, pero puede ser otros usuarios también.
- 5: yo quería decir de “4” porque yo he visto en él un cambio bien dramático, no lo comprendo muy bien porque sería mentira decir que yo lo comprendo, pero él me comentaba hace poco que solo iva a saludar a “1” y que luego se iba a ir del programa, y ahora lo veo más optimista, que quiere seguir, no acepta que es enfermo pero acepta que tiene un problema, y he visto que él trata de no perturbarse tanto la mente sino de abrirse y de pensar que él ya se está abriendo a los demás, porque antes era como una islita y decía “no, yo a fulano de tal no lo acepto”, y hoy veo que se está abriendo y piensa más positivamente.
- 8: yo si es con respecto a los usuarios, esto yo siempre lo he querido decir, he notado que es un grupo como dijo “5” “son optimistas”, pero también he notado que son bastantes participativos y al mismo tiempo es un grupo que trata de empoderarse, y no solamente con aceptar su enfermedad, sino que también allá afuera contra todo ese tipo de personas o instituciones que de alguna u otra manera podría decir que el hecho de que van a marcha lo hacen con plena conciencia de por qué están en una marcha, porque tienen el deber y el derecho de reclamar, es una auto exigencia, consiente o inconscientemente, pero si veo que ellos en ese sentido se auto exigen con el hecho de salir a la calle y expresarse. Y respecto a nosotros los familiares, yo llevo tres años de estar dentro del programa, y con la persona que he estado más cerca es con mi hermana (“7”) pero creo que antes de tres años las dos andábamos dando vía en muchas cosas, andábamos perdidas, quizá desubicadas, a pesar de que una esté estudiando no quiere decir que todo lo sabemos, y podemos ser ignorantes en muchas cosas, más en este tema de salud metal que es muy poco abordado aún por las instituciones educativas, entonces he notado en mi hermana un liderazgo, conocimiento (entre llorando), me alegra saber que está ahí y creo que es una tarea compartida que tenemos las dos compartida por el resto de la vida, solamente.
- 1: bueno una cosa que yo he visto es que la gente siempre me agradece por el programa, y creo que ellos se sienten bien descargados y no se sienten tan solos como familiares en su rol de cuidador.
- 7: lamentablemente no me acuerdo muy bien quienes fueron nuestras compañeras cuando estábamos en el curso, pero he tenido la vivencia con mi hermana (“8”) y también se han notado los cambios, comparto lo que ella ha dicho, nada está lejano a lo que ella ha experimentado también. Y respecto a los usuarios lo que he podido ver en el grupo de arte en el tiempo que tengo de estar compartiendo con ellos los sábados, es que he visto bastante o mayor desenvolvimiento hacia las demás personas, incluso hay una persona que me ha sorprendido en el último año porque es una persona bien expresiva ahora y antes no era así.
- 1: es decir su capacidad de expresarse, comunicarse…
- 7: sus emociones incluso, antes esa persona se veía hasta fría, ahora tiene la capacidad de demostrar una alegría cuando ve a otra persona, es capaz de decirle “estoy bien de verte”.
- 2: uno de los cambios más significativos que yo he podido ver a lo largo de los años es que ellos han aprendido a transformarse a ellos mismos.
- 1: ¿quiere decir más? Porque usted ha visto muchas cosas por muchos años
- 2: Bueno, yo podría decir bastante pero no quiero decirlo todo.
- 1: ¿puede decir algunas cosas?
- El conocimiento que tienen de sí mismos por ejemplo es bien importantísimo, porque ahora las personas se conocen más, hay algo importante también como es el compromiso que tienen, a mí me encanta saber que tanto en el grupo de arte terapia como en el de familiares hay gente bien comprometida, y el compromiso lo asumen porque tienen una experiencia de agradecimiento hacia el programa, entonces el agradecimiento les lleva la compromiso, y es un compromiso que ha pasado el nivel personal, el hecho de que salgan a marchar por ejemplo, eso significa un compromiso social, y eso es muy importante, como también es importante el que ellos construyan una identidad propia.
- 3: quizás tres palabras: empoderamiento, responsabilidad y socialización” estoy desde el 2005 a la fecha, siento que hemos avanzado más en los últimos años que al inicio y quizá el avance y el empoderamiento que ha tenido tanto el familiar como el paciente me ha hecho no desistir.
- 1: ¿por qué piensa que hemos avanzado más desde el 2005?
- 3: porque al principio no sentía que el familiar tuviera una responsabilidad, muchas veces llegué a pensar que llegaban por el fármaco, por el medicamento, hoy no, hoy podríamos mencionar de 5 a 6 familiares que se yo que no nos van a faltar, y en cuanto al usuario, lo siento tan responsable que muchas veces quizá el familiar tarda más en llegar que el usuarios, y con la formación de la Asociación de familiares siento que hemos avanzado y me atrevería a decir que a pasos agigantados, porque si yo me había puesto para jubilarme con ustedes tres años hoy me voy a jubilar en uno, porque siento que ya tienen buen empoderamiento y pueden seguir solos.
- 4: yo quiero decir que mi papá, bueno también mi mamá han influido mucho para que yo no desistiera a seguir viniendo y también quiero recalcar de que afuera no se encuentra trabajo, es bien difícil encontrar uno que uno pueda realizar y que uno esté contento, pero gracias a ACISAM uno se siente útil, tiene un lugar donde ir, porque a veces uno pasa toda la semana en la casa y solo los domingos que va a la iglesia, por lo menos yo ando pensando “sábado hay reunión”, y mi papá que siempre me anda empujando, y ahora que estoy aprendiendo hamaca aunque todavía me falta aprender un poco más, pero eso es bien importante para mí porque como uno no recibe ingresos propios por el problema de no tener un trabajo, por lo menos ACISAM compensa el sentirse útil, ocupado y que uno puede hacer algo bueno y productivo.
- 3: quizá “1” para que no se nos quede fuera porque siento que es algo importante que hay que recalcarnos, y que va en los pasos agigantados que hemos dado, antes, “2” y yo dábamos los 17 talleres, hoy solamente doy dos temas y siento que es un gran avance.
- 1: tomaron esta responsabilidad los familiares.
- 3: tomaron la responsabilidad y uno se dedica a otras cosas, también ha sido una enorme satisfacción de que ellos desarrollen los talleres y no nosotros, también otra cosa importante que hay que mencionar es que antes, aparte de eso, teníamos con “2” la responsabilidad de la visita domiciliaria, hoy lo hacemos en conjunto con un familiar y la perspectiva cambió enormemente, ¿por qué? Porque cuando uno va a acompañados de uno o dos familiares y el familiar también expresa hacia el que vamos a visitar “yo tengo un hermano, yo tengo dos hijos, yo soy madre, soy esposo” cambia la perspectiva que vamos a visitar y decir “no, no estoy solo”, desgraciadamente “1”, que nos quiten las vestiduras de profesionales es muy difícil, es muy difícil, siempre existe esa distancia, mientras que cuando llegamos acompañados siento que esa distancia disminuye enormemente.
- 1: cuando dice “quitar nuestra vestidura profesional” ¿para hacer qué?
- 3: de entrada para la comunicación, nosotros manejamos con “2” al menos llamarnos por nuestros nombres, pero si usted se fija, al menos conmigo “doctora, doctora, doctora”, siempre hay una brecha, siento que siempre nos siguen viendo como tales, quizá con un poco más de confianza.
- 1: entonces, ¿hay muchos beneficios de hacer este trabajo juntos?
- Sí, tenemos mayor ganancia.
- 2: Hay algo que también yo he visto en los familiares a lo largo de los años y eso es algo pero bien importantísimo, y hemos dado un paso agigantado y es la conciencia de los derechos humanos que se tiene en el grupo, eso es importante.
- 1: vamos a entrar en un receso de cinco minutos y después tenemos la última parte.

**Inicia audio 800280**

**CUESTION #3**

- 1: Empezamos por la tercera parte con las preguntas número 3,4 y 5, estas preguntas son un poco difícil de entender pero trataremos de que se entienda lo mejor posible, la primera pregunta es: ¿por qué algunos familiares cuidadores se benefician más del programa que otras?, ahorita estamos con familiares, pero después usaremos la misma pregunta pero con usuarios, pero primeramente empezamos con familiares, otra manera de preguntarla es ¿por qué una familia se involucra más mientras que otras no lo hacen?
- 4: yo podría pensar que si para algunos familiares funciona más el programa es porque tanto cuidador como paciente reciben ayuda de un psiquiatra o de un psicólogo, ayuda profesional, más el medicamento, más el asistir en las actividades que tienen estos programas como ACISAM, ASFAE, de salud mental, siento que la confianza que hay entre el familiar y cuidador es lo que hace de que funcione mejor un programa como ACISAM o AFAPDIM o ASFAE.
- 1: ¿la confianza entre quién?
- La confianza entre el familiar y el cuidador, de seguir las recomendaciones del psiquiatra, tomar el medicamento, de venir al programa, eso hace que sea efectivo el tratamiento de una enfermedad de salud mental.
- 7: yo creo que es por la inconsistencia de algunos familiares, o sea, entre menos constante sea mi asistencia, menos me voy a beneficiar porque voy llevando un proceso sistemática y entre más involucrado está el familiar, está consciente, tienen mayor conocimiento y éste lo lleva a la conciencia y a la sensibilización.
- 1: dijo una manera sistemática, ¿pero por qué una familia tiene una asistencia sistemática y la otra no?
- Por diversos motivos pienso, situación económica, no tienen para costearse los pasajes e ir a las reuniones, también para asistir a las reuniones, el familiar cuidador no tienen el tiempo para asistir a las capacitaciones y dejar a su familiar solo, porque no tiene con quién más dejarlo, lo otro es porque, mucha gente llega con un enfoque demasiado clínico, y a veces se desinteresan con este enfoque que es comunitario y de educación entre pares, y como que desacreditan la información que pueden recibir de los mismos familiares.
- 1: explícame un poco más la diferencia entre el enfoque clínico y la educación entre pares.
- La gente aquí está acostumbrada a que el profesional es el que tiene el conocimiento de las enfermedades y que solo el profesional es quien domina esa área, y que los familiares de personas que tienen enfermedades no son capaces de dominar el tema; entonces cuando llegan al programa y se dan cuenta que son familiares los educadores, pierden el interés, que no vale la pena y que no es un proceso de calidad.
- 1: muy bien, entiendo, gracias.
- 8: lo que he observado es que tenemos la cultura en nuestro país, y muy mala, de que siempre andamos buscando un beneficio personal, llegan la primera vez y no consiguieron lo que ellos querían, a lo mejor que se les diera un dote de medicamento o un donativo de “x” cantidad de dinero, pero ven toda la charla que se dio como improductiva, entonces no es mayor ganancia para estas personas ir a recibir un taller psicoeducativo, entonces es hasta el final o a mediados del curso es que uno empieza a ver los frutos de la asistencia, como lo que decía “7”, a medida que el familiar o la persona cuidadora asiste a los talleres, va viendo los resultados, incluso desde la mitad del curso, en mi caso eso sucedió, y ya para el final yo tenía otro tipo de concepción en el tema de salud mental. En resumen sería que los salvadoreños tenemos una cultura de siempre andamos buscando un beneficio personal y poco nos interesa lo que es intangible, siempre vemos lo que podemos tocar, en este caso lo educativo poco interesa.
- 5: yo no soy familiar cuidador, pero yo comprendo lo que dice “8”, el salvadoreño es muy cómodo, y cuando se le empieza a comprometer en algo entonces se hace para atrás y prefieren pasar en la casa con el paciente que lo acomodan, lo arrinconan en un lugar donde lo tienen escondidito y así sienten ellos que han solucionado el problema, pero en realidad es por comodidad del familiar, el familiar quiere algo milagroso que el paciente de la noche a la mañana esté curado y que ya no tenga ningún problema.
- 9: yo soy una madre cuidadora, ¡madre y padre a la vez! Y no soy una usuaria activa porque no he dado talleres, pero activa de estar constante aquí, si lo soy, incluso cuando tengo problemas de salud por mí o por mis hijos le habló a “2” que no voy a poder llegar, yo siento que esa es responsabilidad.
- 2: son constantes, las familiares que son constantes y terminan creyendo que en verdad es la educación una respuesta son los que se benefician más, es la constancia una de las cosas importantísimas para que las personas se beneficien más, una de las familias que han permanecido más tiempo son las más beneficiadas. Y para mí también hay otra cosa bien importante, regularmente la familia siempre llega con altas expectativas de uno y muchas familias solo esperan recibir, pero en la medida que ellos entienden que también se trata de dar, son más beneficiadas, porque beneficia más una relación de dar y recibir, que una relación solo de recibir, entonces la familia que comparte y dan lo poco que tienen, terminan siendo más beneficiadas.
- 3: para mí es por las falsas expectativas que tiene el familiar del programa, como decimos “voy a cambio de algo” y cuando usted lo compromete dice “no, ya no me parece”, existen situaciones muy puntuales por situaciones económicas, tenemos muchos casos de mamás con dos hijos y si lleva a uno no puede llevar al otro, así tenemos como cinco casos y comprendemos, pero la mayoría esperan que le den víveres, medicamentos, que satisfagamos sus necesidades económicas y al ver que eso no es así dan marcha atrás, y a manera de ejemplo de que tan equivocados están, es que hace más o menos un mes, se incorporó una familia que me llamó 35 veces a mi celular durante un solo día, llegó a buscarme alrededor de cinco veces a mi área laboral para que yo hiciera referencia, pienso que las expectativas eran totalmente equívocas y no dan nada a cambio, qué pedimos nosotros, constancia y que se capaciten para ayudarnos a dar talleres. Entonces el programa no llena sus necesidades, todo lo contrario de los que sí son constantes, como dice “8”, a mediados del taller caen cómo es esto y continúan con nosotros.
- 4: yo tenía una observación que una dijo usted “2”, otra “8”, “3” y “7”, y es que uno busca algún beneficio económico, o buscando medicinas como si fuera un hospital, pero también a veces uno se crea expectativas que en el grupo uno se va a conseguir una su novia, en la parte sentimental o emocional, hoy que vinieron las muchachas estudiantes de EEUU yo bien contento y bien feliz “que una muchacha de estas me hiciera caso” decía yo, como soñador e idealista o fantasioso uno, pero también a parte de buscar un beneficio económico o como si fuera hospital que aquí le doy la medicina, también uno puede pensar que “tal vez ahí está mi pareja” o “ahí consigo una novia”. Probablemente podría ser otro punto.
- 1: esto es una buena transición a la próxima pregunta ¿por qué algunos usuarios/as se benefician más del programa que otros? O ¿por qué algunos usuarios/as se involucran mientras que otros no lo hacen?
- 2: “1”, antes de pasar a esa pregunta quisiera aportar otro punto bien importante y eso es un desafío para el programa, muchos familiares se benefician porque también están cerca de las oficinas de ACISAM, ¿por qué no se beneficias algunos familiares? Porque están muy lejos de ACISAM y no pueden participar, asistir. Hace tres años intencionados hacer un grupo a nivel comunitario, pero para eso el programa tiene que tener más capacidad, entonces realmente es un desafío y es una limitante para muchos familiares porque no se benefician, pero, aquí también quiero decir lo contrario, no siempre los familiares que viven más cerca son los que más se benefician, porque influye mucho las condiciones económicas, porque una familiar que tiene una posición económica que le permite comprar medicamentos no tienen la necesidad de educación, o la educación ya la tiene de otra forma. En nuestro grupo, la mayoría de nuestros miembros su nivel económico es bajo, entonces, la conciencia de la educación y la importancia de la educación para nosotros está más cerca en los niveles medios-bajos.
- 1: ¿qué significa eso?
- Es decir que medianamente satisfacen sus necesidades básicas, alimentación, salud, vivienda, etc., en el programa no tenemos a ningún rico o rica, eso era lo que quería mencionar.
- 1: quiero entender mejor, ¿hay una relación entre ingresos del familiar y participación?
- Yo creo que sí, aunque no siempre es determinante, una familia que tenga un ingreso mayor, tiene sus necesidades satisfechas, y entre éstas pueden comprar sin ningún inconveniente el medicamento, entonces creo de que a muchas familias les es más fácil creer solo en el medicamento, y es más fácil creer que una familia con ingresos bajos tome consciencia de la importancia de la educación que una familia con ingresos bajos.

**CUESTION #4**

- 1: OK, entiendo la respuesta ahora. Vamos entonces a la siguiente pregunta ¿por qué algunos usuarios/as se benefician más del programa que otros? O ¿por qué algunos usuarios/as se involucran mientras que otros no lo hacen?
- 5: yo diría ¿a qué beneficio se refiere? Porque al menos entre los usuarios, el que tiene una habilidad la explota, y no veo que haya preferencia para uno o para otros, porque todos tenemos las mismas oportunidades, si alguien puede hacer algo y lo explota ya es habilidad de él, pero yo veo que todos tenemos el mismo beneficio, tal vez alguien se reciente por algo, pero ya es amor propio de él, que se le encaprichó a “2” o a saber qué, pero de los que estamos allá adentro todos tenemos la misma participación y tenemos las mismas oportunidades.
- 1: quizá otra manera de preguntarla es ¿Por qué el programa ayuda a un usuario más que a otro?
- 6: yo creo que el que está más enfermo lo debería de necesitar más que el que no está enfermo, yo he visto que algunas personas necesitan ayuda, espiritualidad, apoyo.
- 1: entonces las personas con trastornos más graves, quizá el programa les ayuda más a ellos?
- El programa puede ayudar más, pero hay personas que no les gusta tomarse la medicina y dicen que están bien o que se sienten bien.
- 1: entonces, ¿el programa es más beneficioso para las personas que no están negando su enfermedad?
- Sí.
- 4: también el programa es más beneficioso para las personas que asisten al programa, se involucran y tienen el apoyo de su familia. Entonces para esas personas es más funcional el programa de ACISAM- AFAPDIM, que las personas que no asisten y se involucran en el programa.
- 1: sí, pero mi pregunta es porque ellos no tienen interés?
- Quizá porque no tienen interés.
- 9: en mi caso, mi hijo Jorgito, yo siento que él no tiene fuerza de voluntad, por más que lo motive, que le diga cosas bonitas, las veces que ha llegado al programa me lo estoy enamorando desde tres días antes, siento que él se siente bien estando en la casa, él así como es en el programa que no habla, así es en la casa.
- 1: ¿no habla tampoco en la casa?
- No, no habla mucho.
- 1: piensan que la gente con trastornos tienen que alcanzar un nivel de estabilidad antes de que puedan beneficiarse del programa o pueden entrar en cualquier momento de su enfermedad.
- 7: desde mi punto de vista creo que pueden entrar en cualquier momento siempre que ellos tengan el interés y las ganas de asistir, porque ya adentro de los talleres de arte terapia, es donde se van a ir dando los cambios a favor de ellos, por ende va a ir mejorando.
- 3: yo no estoy muy de acuerdo con “7”, ¿por qué? Porque si el paciente está, hablándolo con terminología psiquiátrica, psicótico, muy poco va a funcionar y de hecho puede desestabilizar a los que están funcional, y muchas veces es el familiar el que participa primero que el usuario, cuando el familiar comprende en qué consisten los diferentes áreas del programa, como dicen “9”, empieza a enamorarlos y los empieza a traer, y para muestra un botón, veamos a Iván, él no está medicado, muchas veces cuando se incorpora con nosotros no labora como los demás y viene y va, viene iba, y la medicación es básica, otro aspecto muy importante es el apoyo familiar y lo económico, porque qué sucede si este paciente para venir al programa gasta un dólar de venida y un dólar de regreso, son dos dólares, más el acompañante que no lo va a dejar porque él viene a psi coeducación y él a psicoterapia, más que la terapia abarca casi todo el sábado involucra un tiempo de comida, un break, café, entonces ante todo eso tenemos usuarios que por sus aspectos económicos no vienen al programa.
- 1: ¿alguien más? Estamos hablando de por qué uno usuarios se benefician más del programa que otros.
- 8: el ejemplo que traigo es Amanda, con ella quizá tenemos un diagnóstico de su enfermedad es erróneo, es equívoco, y desde el momento que le dicen y nosotras también (sus hermanas),” tu problema es que tienes esquizofrenia”, peor ella es una persona que lee y que investiga, ella se mete a internet y comienza a buscar los síntomas de este trastorno y ella misma me ha dicho a mí “no, yo no tengo esquizofrenia, yo tal vez soy bipolar” entonces no sé si podría ser una de las causales cuando no hay un diagnóstico certero para el paciente, en la medida que no lo tiene poco aceptará su enfermedad, y si no lo acepta, poco va a disfrutar de los beneficios del programa, porque yo estoy en la idea de que en medida que haya aceptado su enfermedad es como se va a beneficiar, va a poder incorporarse a los talleres, pero si no tiene un diagnóstico adecuado, difícilmente se va a incorporar.
- 4: yo tengo una observación, cuando empezamos estos talleres, comenzamos yendo cada sábado a Guazapa, y de repente primero solamente era hasta el mediodía de 8 a 12, y después lo pusieron de 8 hasta las 4, recuerdo que se empezó con el dibujo y la pintura, teníamos un buen profesor, como dos años pasamos viajando a Guazapa y después solo nos quedamos reuniendo en la casa de ACISAM, porque ya no podíamos estar yendo a Guazapa, esa es la observación de que nos adaptamos a la casa de ACISAM y a estar desde las 9 hasta las 4 de la tarde, luego vinieron los paseos, pero se incorporaron otras personas
- Porque siguen las recomendaciones de su psiquiatra y de su familia.

**CUESTION #5**

- 1: muy bien. Vamos a seguir con la última pregunta para hoy y es ésta: ¿qué diferencias entre familias pueden influir para ser ayudados por el programa? Quizá se parece un poco a la pregunta anterior pero es diferente, es decir cuáles son las características que tiene una familia, que influye o determina si el programa es muy bueno para ellos o no muy bueno. Es una pregunta un poco difícil, vamos a pasar por un círculo para dar una respuesta corta, breve, voy a preguntar una vez más y “5” puede empezar ¿qué diferencias entre familias pueden influir para ser ayudados por el programa?
- 5: la diferencia es la perseverancia, hay familiares que perseveran y otros que no, esa es la diferencia mediática, y la demás es la educación, y si el familiar está dispuesto a involucrarse con el enfermo o solo quiere mandar al enfermo a arte terapia.
- 2: aquí influye mucho las condiciones económicas de las familias, y también en alguna medida el nivel educativo.
- 7: lo mismo iba a decir yo. También el grado de sensibilidad o amor que se tenga hacia el ser querido o usuario.
- 8: lo económico, pero las condiciones de accesibilidad sería otra.
- 1: dígame más de esto, ¿qué es accesibilidad, en qué sentido?
- O sea, no sé si está aunado a lo económico también lo accesible porque están lejos, sí tiene que ver con la economía.
- 2: no solo con eso, porque hay familias que están lejos pero se pueden movilizar y no lo reciben y otros que están cerca pero no lo reciben, las condiciones económicas tienen que ver, pero también lo cercano o lo lejos.
- 8: bueno, esas dos cosas serían: lo económico y lo accesible, es decir la distancia.
- 9: pues no es que copie, pero para mí esa es la respuesta, lo económico y lo accesible, porque en Jorgito he notado eso, que si yo vengo con él o su hermana sí viene, o sea que él no se considera con valor, pienso yo, porque cree que se va a perder, me lo ha dicho muchas veces.
- 2: quizá algo que no quisiera que se nos escapara y que yo he percibido, es la composición familiar, primero, no es lo mismo que tenga mamá y papa o un cuidador especifico; y dos, cuando tenemos dos familiares en la familia, lo que decía la vez pasada “llevo uno, ¿con quién dejo el otro?” y tiene que ver mucho lo económico.
- 1: entonces por ejemplo, en el caso de “9” es ella sola, y tiene dos enfermos con trastornos mentales, pero hay otros niños en casa con problemas también, pero aquí está, entonces éste es un caso contrario a lo que acaba de decir usted, porque ella está en esa situación pero aquí está
- 3: pero igual no bota mi hipótesis.
- 1: pero está hablando en general que la mayoría de familias en su situación no pueden asistir.
- Porque éste es un caso puntual para ella, ¿pero todos los sábados? Esta encuesta no es todos los viernes.
- 1: solamente tratando de entender: entonces para usted las familias que tienen un padre o un cuidador o familias que tienen dos o tres personas enfermas, estas familias tienen más obstáculos para asistir a todo esto?
- Sí.
- 6: me puede decir otra vez la pregunta
- 1: las familias son diferentes ¿verdad? Y algunas se benefician más del programa que otras, si se están beneficiando unas familias más que otras ¿por qué? ¿cuáles son las características diferentes que les ayuda a beneficiarse del programa?
- 6: económicamente hay gente que tiene poco dinero y que no se sabe defender con la medicina, le explicare, a veces la gente tiene dinero y a veces no, y así no pueden tener el medicamento, porque los hospitales los dan, pero algunas veces no, y esto e difícil porque algunas medicinas son más baratas y otras son muy caras.
- 4: yo pienso que en el caso mío, dependo económicamente de mis padres, para mí las personas que no se benefician como otros nos beneficiamos es porque no quieren salir adelante, quieren quedarse enfermos, tristes, no quieren buscar una ayuda o respuesta a su problema, que con ayuda de Dios se la brinda el programa, yo pienso que quizá hay familias que no quieren ayuda o que se resisten a que les ayuden y por eso no quieren salir adelante ni curarse de su enfermedad.
- 1: yo tengo algunos pensamientos sobre esta pregunta también, y una idea entre las diferencias entre familias podría ser por ejemplo la duración del tiempo de la enfermedad, por ejemplo si una familia tiene la enfermedad desde un año, diez o veinte años con la enfermedad ¿esto afecta la capacidad de la familia para participar? ¿sí o no?
- 2: eso depende.
- 1: ¿entonces no?
- Como no, puede afectar, pero también depende de la cronicidad de la enfermedad, es decir una esquizofrenia por ejemplo, bien puesta, desgasta a la familia, si no recibe ayuda la familia cae en una desesperación y en una desesperanza, entonces los años pesan, pero también estamos hablando de la gravedad de la enfermedad, si la persona no ha recibido tratamiento entonces va a desgastar a la familia, entonces si influye el tratamiento.
- 1: ¿alguien más quiere hablar del tiempo de la enfermedad?
- 4: yo pienso que el tiempo de la enfermedad uno puede convivir con la enfermedad hasta el tiempo de fallecer, desde que le diagnosticaron a uno la enfermedad mental, mientras se tenga el apoyo de los padres o algún hermano, hermana, se puede llevar hasta 20 años hasta que una persona fallezca.
- 9: ¿de cuánto tiempo hablamos?
- 1: desde uno hasta el fin de la vida
- 9: pues en mi caso que a uno le comenzó en la adolescencia, uno va a cumplir 40 años, el otro 37, ¿cuántos años son ya? ¿Y sumados?
- 1: hubiera sido diferente para tí entrar en el principio de sus enfermedades o después de 10 años o 25 años hoy ¿hubiera sido diferente para tí su involucramiento en el programa o hubiera sido lo mismo?
- Quizá no, porque si a estas alturas hubiera sido el descubrimiento de su enfermedad, yo no tuviera capacidad, yo me siento diferente, ya me siento bastante cansada.
- 1: ¿piensas que sus vidas hoy hubiera sido mejor si hubiera entrado al programa hace 20 años?
- Es posible.
- 7: yo pienso que sí influye el tiempo, porque por ejemplo si nosotras hubiéramos entrado desde el inicio del diagnóstico de la enfermedad de mi hermana y no hubiéramos dejado que la viera un psiquiatra y otros y que le cambiaran el medicamento y el diagnóstico, nosotras hubiéramos tenido la educación y la información suficiente para saber tratar a nuestra hermana, y a lo mejor la enfermedad no se hubiera puesto tan crónica, y el hecho de que nosotras estuviéramos educadas, hubiera influido en trasladar esa información al resto de la familia, para que comprendiera y ayudara también a la situación de nuestra hermana, entonces entre más rápido se entra al programa desde que se inicia la enfermedad, creo que mayor es el impacto que se puede lograr.
- 8: yo creo que el programa, puede tener su factor preventivo en ese sentido (lo que dijo “7”) antes de que la enfermedad se vuelva más crónica, porque en el caso de “mi hermana,” al principio la vio uno de los mejores psiquiatras del país, el Dr. “P”, y no fue el diagnostico de esquizofrenia lo que él dio, sino una depresión crónica nada más y que a raíz de eso alucinó, y que después de tres meses él iba a decir qué era, en ese sentido si nosotras hubiéramos tenido conocimiento, jamás hubiéramos permitido que se la llevaran para otro psiquiatra, y de una sola vez la trataron como esquizofrénica, esa es la desinformación que existe, entonces el programa podría ser un programa preventivo.
- 5: yo quiero participar. A mí me parece que es conforme a la enfermedad lo que limita la enfermedad, a mí por ejemplo con clorpromacina, me limita la enfermedad porque me “noquea” la medicina, al principio yo dormía, me daba sueño tarde en la noche y eran las 12 del mediodía y no me levantaba, entonces me estaba limitando, pero yo dije “voy a buscar una forma de cómo vencer esto” y no someterme a la forma que me estaba diciendo la enfermedad, entonces primero me abrigué en la iglesia, salía de la casa, porque mi familia me criticaba y decía que yo no tenía nada, que solo era haraganería mía de estar en la cama y empecé a buscar un derrotero mejor, como fui venciendo las imitaciones que me presentaba la enfermedad, fui superándolo. Pero sí creo que el tiempo puede ser un obstáculo y puede ser una ayuda, porque no quiere decir que todo paciente que es esquizofrénico, tiene que cronificarse, sino que también tiene que ver mucho las condiciones ambientales en que uno se mueve y las colaboraciones que uno recibe.
- 1: es tiempo de terminar y solo quiero hacer una pequeña encuesta y es sobre la importancia de la actitud de la familia hacia la persona con trastorno en la familia ¿piensa usted que la actitud de la familia hacia el usuario es bien importante, no sé o muy importante?
- (Al unísono todos contestan muy importante), luego en una pregunta individual, todos reafirman que es muy importante.
- 1: vamos a proseguir a la cena, quiero agradecerles mucho por su participación y seguimos mañana a la 9:am.

**(NO SE GRAVÓ LA PREGUNTA) inicia audio 800281**

3: acordémonos que la esquizofrenia tiene un proceso, va a llegar un momento en que mi esquizofrénico va a llegar a un momento residual, cuando yo he tratado de promover el programa, es más fácil que la gente acuda al inicio de la enfermedad, he identificado esquizofrénicos de 50, 55 años. ¿Y qué obtengo cuando promuevo el venir aquí? “fíjese que él está bien así, él es tranquilo, él no molesta” aunque lo tenga encerradito. Esa es mi perspectiva como médico, cuando es un adolescente, un adulto joven el familiar dice “sí, ayúdenme” ¿pero qué pasa en aquellos casos cuando el usuario ya es un adulto de 40 ó 45? Ya tiene los síntomas residuales, y cuando yo hablo con los familiares “ mire fíjese que ahí hacemos terapia, hacen hamacas, bisutería” “no así está bien, no es agresivo”, o sea como que ya no me interesa que él sea sociable, incluso he tenido pacientes que son hijos de profesores y me dicen “no así está bien”, como que pareciera, que vamos a ser más efectivos o nos buscan más en los inicios, desgraciadamente, no conocen mucho estos programas porque muchas veces en la consulta general los he capturado porque el paciente llega por hipertensión o por diabetes y su enfermedad no mejora, y a veces le pregunto ¿tiene problemas? Me responde “fíjese que tengo un hijo con un trastorno mental” entonces es más fácil, siento yo, cuando que ellos vengan cuando están los primeros brotes, que cuando ya están los síntomas residuales, es la perspectiva clínica del tiempo.

- 2: agradecerles, no solo por el hecho de estar aquí, realmente para mí es muy impresionante saber que pueden expresarse con libertad y “6” por ejemplo, se expresa, ¡wow! Muy bien, antes no lo hacía. “4”, la capacidad que tiene de hablar, “5”; eso demuestra que han tenido y están empoderados, solo por la capacidad de hablar, su soltura….me alegra mucho y gracias.
- **Inicia audio 800282**

1: Otra vez quiero agradecer a todos por venir y asistir a esta investigación del programa de familia, quiero darle la bienvenida a “10” por venir también hoy, ayer discutimos el concepto de bienestar de salud mental, también discutimos cómo es el bienestar para usuarios cuáles cambios hemos visto en las personas que han participado en el programa, y por qué algunas familiares y usuarios se benefician y otros no. Tenemos más preguntas hoy, primeramente quiero repasar las directrices rápidamente... la primera pregunta es sobre el efecto del programa, y la primera pregunta es ¿el programa es positivo?, pero la pregunta es ¿cómo, cómo es efectivo? Y queremos empezar con el nivel individual, entonces para los individuos que participan en el programa o que son beneficiados indirectamente, cómo es de beneficioso para ellos también.

**CUESTION #6**

- 4: como individuo el programa es positivo porque me mantiene ocupado canalizando la energía en algo buena.
- 1: muy buena respuesta, porque es directa y muy corta, es lo que necesitamos.
- 5: para mí es positivo porque tengo una atención personalizada.
- 6: para mí es positivo porque aprendo más y mi actualidad mental es aprender más.
- 10: creo que uno encuentra las respuestas, a nivel individual uno anda buscando respuestas de la situación en la que uno está, y el programa le brinda respuestas de qué es lo que está pasando en casa, sale uno de la agonía.
- 8: es positivo porque es un medio de desahogo para el familiar cuidador.
- 1: ¿desahogo es para salir del agua donde uno está?
- 8: o sea, por lo general una guarda todos los sentimientos a escondidas, no los expresan, y en cambio ahí (en el programa) hay otra gente que tiene la misma situación o casi igual y se puede hablar en libertad o en confianza, entonces es un medio de libertad de expresión.
- 7: para mí es positivo a nivel individual porque encontré apoyo y desahogo.
- 3: para mí es positivo porque ayudo sin tener un jefe, soy libre trabajo sin estrés ni presión y también es positivo porque considero que puedo ayudar a más gente que nos necesita, sin necesidad que me busquen en el trabajo, yo me siento bien.
- 1: no entendí la última cosa.
- 3: o sea, no ayudo por obligación sino por voluntad.
- 9: para mí es positivo porque en momentos de emergencia he recibido apoyo y también es un lugar donde uno se va a desestresar, vamos a llorar y sabemos que ahí queda todo, todos nos comprendemos porque somos una familia.
- 2: es positivo porque brinda conocimiento a la persona de sí mismo y de lo que está pasando alrededor de su casa a raíz de la enfermedad mental.
- 1: lo que he visto y lo que me ha dicho la gente es un aumento en la autoestima, por ejemplo cuando uno puede trabajar un poquito y ganar un poquito se siente mejor de sí mismo. Y la otra cosa que yo he visto es un proceso de cambio de empoderamiento, como un proceso de cambio de empoderamiento, de no entender nada, a entender a su ser querido y a sí mismo, entender la justicia a tener el poder de salir a la calle a demandar sus derechos, es un proceso de muchos cambios.
- 4: también sirve para sentirse útil.
- 6: útil a la sociedad.
- 5: para mí es positivo porque en lugar de ponerle una carga a uno le ayudan a sobrellevar el problema, o sea que en lugar de ponerle a uno obligaciones, le ayudan, le quitan las responsabilidades de lidiar con uno a la familia también, o sea yo salgo a un lugar donde no me ponen una obligación de que tengo que portarme de alguna manera o que si hago algo inadecuado no me van a recriminar por eso, me siento libre pues.
- 1: ¿cómo ponemos eso?
- 2: quizá está hablando de que se siente aceptado, una aceptación dentro del grupo, se siente libre, puede comportarse como es, nadie lo limita.
- 10: eso es puede actuar como es él.
- 1: ser sí mismo.

Muy bien, entonces seguimos con la próxima que es el nivel familiar, ¿cómo es positivo el efecto de este programa para la familia?

- 4: yo pienso que mis padres se sienten bien con este tipo de programas donde se recibe apoyo a través del arte terapia y de las actividades que se hacen, entonces la familia se siente bien por la existencia de estos tipos de programas.
- 5: para mí en lo familiar le ayuda porque la familia puede descansar un poquito de tener al usuario en la casa todo el tiempo, o sea que le abre una puerta de esperanza al familiar pueda si uno pasa solo en la casa, uno hastía, la familia se aburre de uno, mientras que si uno platica con más personas y se siente útil ya no hostiga a la familia, porque por ejemplo, yo celaba mucho a mi señora y quería que ella solo estuviera conmigo y cualquier cosa que ella hacía yo la miraba mal, mientras que hoy los dos nos portamos bien, uno haciendo lo que tiene que hacer y otro yendo a arte terapia.
- 6: pues para mí, mi mamá me dice que me ponga a hacer algo en la casa, a barrer, a trapear, a veces hago mandados como ir a traer crema y mi tío ya nos llevamos bien, de vez en cuando peleamos pero ya estamos cambiando, solamente.
- 10: yo pienso que a nivel familiar se elevan los niveles de comprensión, y uno lleva a la familia que no llega al grupo, ese sería quizá el indirecto, pero nosotros tenemos familiares que no llegan, entonces uno leva es nivel de comprensión hacia los familiares, y bajan los niveles de confrontación porque hay una presión, mejora con el tiempo, bajan los niveles de confrontación entre familiares con el familiar.
- 1: diga un poco más de cómo ayuda a los familiares que no llegan al programa.
- 10: yo tengo hermanos que no están muy interesados en saber de mi hermano, porque “R” con sus medicamentos se estabiliza enormemente, entonces la familia olvida ciertas condiciones individuales, en nuestro caso somos tres (los cuidadores) del grupo familiar de seis, a pesar de eso uno no comprende, que recibió el programa, y son muy buenos aportes, mi papá pues ya falleció y hoy estoy yo, pero el hecho de que yo esté en la casa deteniendo a la familia diciendo no estas conductas son bajo lo normal han disminuido, disminuye la presión hacia el familiar “que por qué no trabajas”, “que por qué mucho estas molestando”, “por qué pedís esto”, y hay condiciones muy individuales de él que lo hacen ser como es, entonces comprenderlo es lo que nos ha facilitado bastante, en mi experiencia el programa lo que me dio fue respuestas, porque nosotros nos preguntábamos mil y una cosa.
- 9: en mi caso no hay hermana, solo soy yo, entonces yo he tratado mucho de absorber lo que pasa en el programa para hacerme la vida más fácil, más bonita, sociable, como dice el dicho aquí “se hace una la de los panes”.
- 2: le ha hecho la vida más fácil en el sentido de?
- 9: lo que he aprendido en el programa para entender el cuido de ellos, todo lo que se da en el programa.
- 1: una pregunta para “9”, hemos hablado un poco de su situación, y tiene dos enfermos con trastornos mentales en la casa, pero tiene otra con discapacidad también ¿el programa le ha ayudado con otras personas en la familia y no solamente con los de trastorno mentales?
- 9: pues yo trato de enseñarles lo que he aprendido, “miren, es así” como he aprendido que tenemos que cuidar a sus hermanos y para entendernos a nosotros también, a la que no escucha le digo yo “no le grites porque de toda manera no te escucha, mejor acércate y la tocas y le hablas” es que yo tengo que tener ese cuidado cuando estoy en la casa, pero cuando están solos es otra cosa.
- 10: “1”, por qué hizo diferencia usted ahorita entre discapacidad y trastorno, para ir comprendiendo.

1: bueno no es muy común para tener una familia en la que hay un cuidador estando otras personas con diferente tipos de discapacidades, entonces yo quería entender un poco si este programa tiene efecto no solamente en las relaciones con trastorno ,mental en la casa, sino también los demás en la casa que tienen discapacidades.

10: ¿pero usted ve a estas personas que tienen esta condición de enfermedad mental como discapacidad?

- 1: como no. Y hay muchas opiniones diferentes, hay unos que se sienten “no soy discapacitado” y hay otros que sienten que soy muy discapacitado por mi trastorno, y hay otros que sí se sientes discapacitados pero capacitados a la vez para hacer muchas cosas, pero los usuarios tienen muchas perspectivas sobre esto. ¿alguién más quiere hacer comentario sobre los efectos positivos a nivel familiar de nuestro programa?
- 7: sí, yo. A nivel familiar ayuda a la unificación, porque luego de que se participa en el curso y una se queda en el programa, desaparecen un montón de mitos y también de culpas que en la familia siempre andan rebotando de “vos”, “y que yo”, “y que aquí” “y que allá”, entonces siento que el programa, a partir del conocimiento que brinda, ayuda a unificar a la familia.
- 8: quería comentar de que lo que tal vez puede lograr son mayores niveles de coordinación, en este caso quizá porque “7” y yo hemos recibido el taller, hay más coordinación cuando se ha tenido afuera a “A” del Hogar, en cosas que yo no pueda, ella las puede hacer y cosas que ella no pueda yo las puedo hacer, hay coordinación, pero trasladar ese mensaje al resto de la familia se complica un poco porque al momento nosotras no logramos que los demás de la familia se incluyan en el programa y eso que les hemos hablado un montón, y que “2” les ha hecho visitas y la parte de recibir los talleres, solamente ha correspondido a nosotras y ha habido un interés por nosotras, sin embargo lo que si bajamos y también es notorio entre nosotras es no estar tanto a la defensiva con los demás, porque yo era una, si me echaban la culpa del problema de “A” yo tenía también mi letanía de reclamos para ellos, confrontábamos.
- 3: quizá salga fuera de contexto lo que yo diga, a nivel familiar a mí en qué me ha ayudado.
- 1: o puede hacer comentarios sobre lo que usted ha visto en las familias en su propio nivel.
- 3: ha bueno, en las familias he visto, primero: que disminuye la confrontación, segundo, así de manera puntual, hay más comprensión por el usuario, por ejemplo algo que no se me va a olvidar a mí es cuando conocimos a la Niña “E”, sus primeras quejas eran “es que “J”, ¡como fuma, como fuma!”, luego ella llegó a la comprensión que era parte de, cuando conocimos a Niña “C” y visitábamos a “W”, “es que me da pena que vengan”, y le enseñamos que ese era el mundo de “W” y aprendió a respetarlo. Siento que a nivel familiar, el familiar se vuelve más comprensivo, más tolerante, entiende más la enfermedad que adolece su usuario. ¿Y en mi familia cómo me ha ayudado esto? Quizá a crear conciencia a mis hijos, a mi descendencia de que en este mundo hay que devolver lo que se nos da, sin necesidad de recibir algo a cambio.
- 5: en mi familia, María valora más mi cambio, quizá porque vivimos en el mismo techo, y yo siento que a veces al vida no es justa porque ella disfruta el cambio mío, pero todo lo que yo he recibido se lo debo a ella, y mi familia no saben cómo es que yo puedo comportarme mejor hoy, porque ellos nunca me han preguntado a mí “¿qué es lo que sentís?” ni me han preguntado “¿qué es lo que te pasa?” ni nada, ellos solo toman medidas y yo solo soy el que acata esas medidas, mi mamá nunca me ha preguntado “
  ¿y qué es lo que estuviste haciendo?”, los frutos que estoy dando no los valora ella, ella siente normal que yo me comporte así, pero eso es una lucha, y mi otra hermana es indiferente, ella en lugar de ser más condescendiente conmigo, me ha olvidado, o sea que según el grado de dificultad de la situación, así es el protagonismo del familiar, porque si yo no tuviera a “M”, porque fue una lucha estar con “M”, mi hermana hubiera tomado las cartas en el asunto y yo no sería la persona desenvuelta que soy, sino que estaría todo cohibido, que si mi hermana quisiera que yo haga algo lo tengo que hacer, mientras que yo me siento más desenvuelto y “M” también pero los frutos que he dado son gracias a “M”, y los beneficios que he recibido también, no me lo dice pero ahí vamos metiéndonos poquito a poco en las cosas… a veces el familiar quiere sacar provecho del paciente y no sabe cómo le ha costado a este paciente la conducta que hoy tiene.
- 2: quiero decir algo que me parece importantísimo además de lo que se ha dicho, y es que creo que a nivel familiar el programa ayuda a que las familias comprendan que hay dinámicas familiares o relaciones dentro de la familia que pueden ayudar o pueden afectarle a la personas, por ejemplo, yo tengo una gran diferencia entre la relación que hoy funciona en “7” y “8” con relación a “A”, con relación a la independencia pero por ejemplo, cuando hablamos de “S”, a ella hay que enseñarle que para fomentar un poco la independencia y la autonomía es que no le esté respondiendo constantemente a todo lo que le pregunta “R”, por ejemplo, porque eso crea una relación de dependencia, y este tipo de relación afecta mucho psicológicamente el autoestima, entonces la relación de las dinámicas familiares ayuda mucho a que el familiar cese la presión, la exigencia y disfrute después, un nivel de independencia, porque tanta pregunta a un familiar las 24 horas, todos los días, todos los años, afecta, se cansan hasta de la misma pregunta, entonces para que ella deje de preguntar, el familiar tiene que poner y colaborar de su parte, para que le permita a la persona encontrar por sí misma una respuesta.

1: bueno, seguimos entonces con el nivel comunitario, efectos positivos a nivel comunitario.

- 5: en lo comunitario, cuando tuve la primer crisis, la familia se avergonzó, y me llevó a otra casa, sin motivo aparente abandonaron la casa donde estaba, pero yo me sentí que me quitaron mi arraigo, pero yo siempre pensaba en la casa donde crecí, donde me conocen, donde me han visto desde niño, mayor y me han visto cómo ha pasado mi vida, pero a nivel comunitario yo vería la vida relajada, no tengo una amistad profunda con alguien de la comunidad, cuando salgo los saludo, solo los hermanos de la iglesia me preguntan por qué ya no llego, por qué me he alejado…
- 1: “5” entonces pensando en su participación en el programa, ¿cómo ha cambiado la situación entre usted y las personas de la comunidad por efecto del programa?, ¿ha cambiado sus relaciones con los demás o no?
- 5: bueno, o sea que yo antes del programa no tenía otra alternativa, tenía que quedarme en la casa, porque la comunidad no es un ente que incida en la vida de uno, mientras que con el programa yo puedo elegir entre estar solo con una clase de persona, entre comillas normal, o sentirme bien entre pacientes.
- 1: ¿y ahora?
- 5: ahora yo me siento mejor, la comunidad también, creo que me ve distinto, me preguntan ¿y qué haces en el tiempo libre? Y yo les digo “hago hamacas” y me dicen “hoy es el tiempo de las hamacas porque viene Semana Santa, te va a ir bien”, y me dan ánimo.
- 1: bueno, gracias.
- 9: en mi caso, a nivel comunitario me siento hoy mejor, porque a Jorgito como ya no lo ven con la cajetilla de cigarros, ya no lo llaman “hey! Noyola vení” y era para quitarle los cigarros, y no sé qué cosas le decían que él se enfurecía y se iba a desquitar conmigo, a exigirme otra cajetilla de cigarros; ahora no, hoy solo le dicen “hola Noyola”, “Hola Coqui”, pero hasta ahí, yo les digo que si les dicen bayuncadas (tonterías), no les responda, usted camine, no se quede caminando con ellos porque algo feo le van a decir, pero él les habla, es bien sociable, también “M”, él les habla a los niños, juega con ellos, platica con los adultos, hoy me siento con un poquito de confianza de mandarlo a la tienda a “J” para que valla a traer lo que uno está cocinando, a traer las tortillas, va a dejar la basura al contenedor, ya no tengo aquella cosa de que “se fue! Voy a ver por qué no regresa”, (preocupada) porque él al ratito va de regreso, no es como antes, que él se iba y se lo llevaban a terminarle los cigarros y a molestarlo. Una vecina me dice “me da gusto ver a Jorge porque ya está mejor, él ya saluda y platica; yo quisiera que mi hermana estuviera mejor” porque hay una persona de ahí (de la comunidad) que tiene enfermedad mental, y en ese aspecto ya me siento mejor, gracias a Dios.
- 4: en mi caso vivo en la Colonia “S M”, pero casi no hablo con los vecinos, antes era amigos de algunos, pero mi abuela me dijo que evitar no era cobardía, entonces realmente la mistad con mis vecinos es nula, la gente hoy vive en una colonia, mañana se pasa a otra, sin embargo no me gusta meterme en problemas con las personas, evito hasta donde puedo, quizá solo en la iglesia puedo comentarlo a algunos amigos, solo a ellos le he comentado que estoy viniendo a este grupo de arte terapia, para que vean que no solo paso en mi casa viendo televisión, tipo muñeco de sala, sino que trato la forma de a pesar que no puedo conseguir un empleo bueno, puedo sentirme útil y hacer cosas buenas, y tratarle de decirle a la gente que estoy tratando de salir adelante, a algunos amigos les comento a través del arte terapia aprendo pintura, dibujo, estoy aprendiendo a hacer hamacas.
- 1: ¿y todo esto ha mejorado su relación con el vecindario?
- 4: es que yo no me meto casi con los vecinos, solamente algunos amigos que tengo en la iglesia, ha mejorado más individualmente y familiarmente.
- 6: en mi caso me llevo bien con todos en la colonia, saludo a todos y me he hecho chera (amiga) de toda la colonia.
- 1: ¿y usted era así antes o no?
- 6: no, era más callada, pero siempre saludaba, pero hoy tengo una amiga….ella me ha dicho que he cambiado bastante y me pregunta a dónde voy los sábados…ella comprende la enfermedad somos muy buenas amigas… es muy agradable estar con la familia de ella, porque me lleva a su casa, con el hermano también jugamos pelota y la familia de ella me ha aceptado mucho como soy…
- 1: y ahora que entiende mejor su enfermedad y su sentimientos, y cómo comunicarse ¿es más fácil hablar de todas estas cosas?
- 6: con mi amiga sí, pero con otras personas me cuesta más.
- 7: a nivel comunitario el programa me ha ayudado a ser más comprensiva y sensible ante los problemas de las demás personas, creo que he alcanzado un alto grado de sensibilidad social, y sí puedo decir que el programa fortaleció esa parte en mí, nunca he sido una persona indiferente a los problemas de los demás, pero esta problemática de las enfermedades mentales, creo que es una de las cosas que más puede sensibilizar a las personas por la misma naturaleza de la enfermedad, y ahora cada vez que yo puedo ayudar en cualquier parte que sea, no solo en la colonia o en la comunidad, lo hago, y con mucha mayor empatía que antes.
- 1: ¿ellos a ella (Amanda)?
- 7: No, de mi parte hacia la comunidad.
- 10: yo veo la parte de la pregunta en dos niveles: 1, cómo yo con el programa de familia a familia he mejorado mi partición en la comunidad, y en ese sentido lo que ha mejorado es bajar los niveles del estereotipo, del auto estigma, porque a veces uno se limitaba a querer relacionarse con los demás por la condición de mi familiar, en eso es evidente que el programa a uno le ayuda bastante, luego veo en la otra línea qué es lo que el programa de familia a familia hace en las comunidades, que eso es otro nivel un poco débil porque nunca hemos buscado a fortalecer las comunidades, sino que a nivel departamental, i a nivel regional, entonces ahí habría que fortalecer y formar vínculos de trabajo quizá con las alcaldías, pero mejorar mi participación como individuo en la comunidad sí, pero la sensibilización de las comunidades hay que trabajarlas un poco más.
- 8: fíjense que a mí el programa, con eso de la sensibilidad que siempre ha sido una característica mía, no soy ajena al dolor o problemas de los demás, pero sin caer en romanticismos y cosas así, pero en mi caso personal, el programa me ha ayudado a mejorar el diálogo, que es una de las cosas que la mayoría de salvadoreños tenemos truncado, no sabemos dialogar, no sabemos escuchar, entonces para mí antes, enfrentarme al resto de opiniones de una mayoría era terrible, porque yo quería imponer hasta cierto punto mi postura, y que los demás no estén de acuerdo me molestaba, pero a medida que recibí los talleres empecé a entender y a cambiar esa parte, respetar las opiniones de los demás y llegar a consenso, entonces en la parte comunitaria yo podría decir que el programa es positivo en el fortalecimiento del dialogo.
- 2: yo quiero compartirles algo, creo que el programa tiene un efecto en le familiar para que éste, comprenda y tolere el estigma social, mis familiares que vienen al programa ya no se sienten tan atacados con el estigma social de la comunidad, quizá escuchan lo mismo, pero ahora su comprensión es diferente, y en ese sentido creo que a nivel comunitario el programa crea o ayuda a crear una conciencia de derechos humanos, porque muchos familiares por ejemplo ahora ante el estigma social no se ponen a llorar, ya no se sienten mal, desdichados, discriminados, ahora hay una cierta tolerancia, en el sentido de que el que dice el estigma no conoce, no sabe, esa es una lógica, y por otro lado, en lugar de enojarse y ponerse a pelear con el otro “no digas esto” les habla un poco sobre lo que es un diagnóstico de enfermedad mental y eso sí que es bien importante a nivel comunitario.
- 5: voy a decir esto porque es una espinita que tengo metida, en realidad en mi colonia no hay movimiento comunal, pero la gente con la que uno se topa puede quererlo manipular, mientras que el programa a mí me ha dado un blindaje, porque por ejemplo había un señora que me decía “mira si tu mujer no te hace caso dale” y en verdad sí me ha dado un blindaje, o sea diferencio cuando una persona me quiere ayudar o me quiere hundir, me quiere poner una cascarita para que me resbale.
- 3: desde mi punto de vista, me cuesta no estar fuera de contexto pero considero que a nivel comunitario es donde deberíamos trabajar la salud mental, porque el psiquiátrico es donde deberíamos enviar las crisis, pero a nivel comunitario donde podemos prevenir, donde podemos identificar de forma oportuna, sin salirnos del contexto, sin separar al usuario de la familia; yo me traslado “2”, a cuando comenzamos a trabajar en el Distrito Italia, que es una zona altamente vulnerable, drogas y pandillas, había un respeto hacia las actividades que hacíamos, porque incluso, en la esquina de la unidad se ponían a hacer renta y a traficar droga, y yo sentí que en los cinco años que yo pasé ahí, nunca hubo un problema con un usuario mío, y es ahí donde empezamos a identificar usuarios que hasta el día de hoy todavía están con nosotros, y si me tocara escoger entre trabajar en el hospital o en la comunidad, me quedo en la comunidad trabajando.
- 2: yo quiero reengancharme si es posible “1”. Aunado a lo que dijo “3” como “10”, el programa a nivel comunitario tiene una deuda, porque no hemos hecho trabajo en ese nivel, y el trabajo se ha planteado y se ha dejado ahí esperando, porque sería un enorme beneficio, cuando íbamos a Santo Tomás y también a Tacanagua, llevábamos una estrategia de sensibilidad, porque allá es común que les tiren piedras, que los tengan encadenados, encarcelados, y eso nosotros lo vemos, como también parte de la estrategia era sensibilizar y organizarnos a nivel comunitario, y llegar a hablar y hacer grupos de familia a familia, sería un gran crecimiento y cobertura si a nivel comunitario hiciéramos grupos de familia a familia capacitáramos líderes, y estos mismos lideres multiplicaran lo que nosotros hacemos aquí, en la sede de ACISAM, los familiares multiplican los talleres, pero a nivel comunitario sería más esfuerzo, porque no todos se apuntan a nivel comunitario, y hay que también hablarlo, implica mayor compromiso, y el compromiso del programa es darle las herramientas necesarias, incluyendo los medios para implementarse.
- 3: “1” pero entonces yo tengo una pregunta, ¿ por qué “2” cuando estuve yo logramos hacer un grupo que hasta el día de hoy conservamos muchos usuarios, que de hecho ahí aprendimos a identificar gente que nos apoya como Niña “M”, porque no volverlo a hacer?, ¿en qué está, en los líderes? Yo toda la vida les he dicho, ACISAM o AFAPDIM, no es “2”, no soy yo algún día me voy a jubilar, y entonces qué pasó con ese grupo? Yo me pregunto. “1”, hay otro médico, hay otro psicólogo, de hecho creo que nos ayudaron en nuestra época de terapistas, ¿por qué no siguieron? Esa es mi pregunta.
- 10: yo pienso que también el programa de familia a familia debe de tener visión, misión y objetivos, focalizados a un sector determinado, pienso que para que sea más estable la capacitación o la formación a nivel comunitario, debe de fortalecerse los talleres, ampliarlos, no solamente que sean formativos a nivel de qué tipo de enfermedades mentales hay, sino que otro tipo de temas para empoderar a la comunidad, visualizo que debe de haber un referente clave en los movimientos comunales, o sea líderes de la comunidad para que, si hay una visión de llevar el programa de familia a familia a fortalecer el nivel comunitario, dentro de la comunidad siempre, siempre debe de haber alguien que esté coordinando para que hale a los que están en esa cangrejera. Yo pienso que se debe tener lazos con la gente que coordina…pero pienso que la clave es buscar líderes comunales para fortalecer, si se ve una visión de llevar este programa a comunidades y fortaleciendo el programa en sí, los talleres con otros temas más.
- 1: vamos a cerrar este espacio para tener el refrigerio si nadie están en contra.

**Inicia audio 800283**

- 1: vamos a seguir otra vez y vamos a seguir ahora con cómo es el programa de positivo en el nivel organizacional, y también les animo a tener sus respuestas cortas y muy enfocadas en las preguntas, entonces: efectos positivos del programa en el nivel organizacional
- 8: fomento liderazgos en sentido horizontal
- 1: es decir no solamente liderazgos sino también?
- Es que la mayoría siempre sigue una cuestión jerárquica, o sea de arriba hacia abajo y los de abajo, ni modo, hacia arriba, y este (el modelo del programa) es más así: estamos a la par, ni vos arriba ni yo abajo, a la par.
- 7: empoderamiento.
- 1: ¿cómo?
- La gente se apodera de conocimientos, de sus derechos y a partir de eso lucha, se defiende.
- 1: para mí una cosa sería la organización de instituciones y programas, es decir la formación de este programa en ACISAM, la formación de AFAPDIM y todo lo que implica organización, por ejemplo podemos ser parte de mesas, de foro, de diferentes cosas porque ya tenemos una entidad, una organización.
- 3: yo siento que el nivel organizacional nos ha servido para dejar de ser invisibles y nos identifica.
- 4: yo entiendo por organizacional cómo está constituido ACISAM, quiénes son los líderes, los que dirigen, cómo organizan los programas que van a dar durante el año a todos los usuarios de ACISAM, cómo ustedes organizan, planean y planifican el proyecto.
- 2: todavía no le he captado la idea de cómo ha sido de positivo el programa a nivel organizacional.
- O sea que organizacionalmente todos lo que ustedes planificaron, como los líderes que están a cargo del grupo ha funcionado porque el programa ha salido así como lo planearon, lo han organizado muy bien, por eso es funcional, algo así entiendo.
- 3: me imagino que es algo así como la planificación, sirve para la planificación.
- 1: y con ésta podemos llevar programas.
- 4: sí.
- 6: como planificar, hacer otro grupo en otra parte y nada más.
- 2: creo que el programa ha contribuido a nivel organizacional en dos cosas uno a darle identidad al grupo de familiares, y eso si es bien importantísimo, una identidad. Y lo otro es que ha servido para crear redes de apoyo, profesionales y de otras organizaciones, lo que “1” decía, la Asociación ahora está conectada a diferentes lugares.
- 1: y ésta fomenta a la colaboración.
- 7: es positivo porque también conlleva a la incidencia en políticas públicas.
- 5: despierta conciencia en la persona, o sea que la gente es consciente que si no se organiza, nunca va a superar sus problemas.
- 1: despierta conciencia para organizarse.
- 2: pero también aquí entra la lógica de que uno solo es difícil pero organizados es más fácil. Un ejemplo es la Ley de Medicamentos, ¿cuándo solos, AFAPDIM y ASFAE iban a llenar las calles por la Ley? Y el día de la marcha éramos miles y miles.
- 1: la siguiente es a nivel nacional, es decir de toda la sociedad.
- 2: ¿cómo ha sido positivo el programa a nivel nacional?
- Se ha posicionado en las instituciones estatales, por ejemplo en el Ministerio de Salud ha sido tomado en cuenta. El programa da la pauta para que la membresía se posiciones en las instituciones por ejemplo en el CONAIPD, pero si hablamos específicamente del programa es que ha sido tomado en cuenta, ha llamado el interés en el sector salud, en este caso con el Ministerio.
- 2: es como que le ha facilitado el programa para recibir el reconocimiento de instituciones, y yo pienso de que se posicione con los temas de salud mental y los derechos humanos.
- 8: eso es lo que permite el programa, porque por sí solo difícilmente, tiene que haber un proceso educativo de los familiares, entonces son los familiares quienes lo posicionan.
- 10: “1”, a mí me gustaría que me diera el contexto de esta última parte, por qué se ve, cuál es la misión de esa última parte.
- 1: estamos tratando de entender mejor los beneficios del programa, en general en las investigaciones hechas en diferentes partes del mundo siempre se enfocan a nivel personal de la familia o de la persona con trastorno mental, pero para mí yo digo que el programa tiene mucho más efecto que solo a una persona con una enfermedad o la familia, también hay efecto en la comunidad, en organizaciones de la sociedad civil y también hay efecto a nivel nacional, entonces estamos tratando de capturar toda esta información, pero solamente lo hago por nivel.
- 10: lo que veo yo en el impacto a nivel nacional es de que a través del programa y de la organización, del colectivo a través de asociaciones se han obtenido espacios, lo que dice “8”, de participación, de incidencia, a través de eso, porque este modelo que se tiene el programa es bien visionario y muy positivo y hay gente yo estoy segura que… (interrumpe “4”)
- 1: espérate (a “4”). Pero sí puedes decir más específicamente cómo es muy positivo, por qué.
- Es que el tema de la salud mental, la discapacidad mental o psicosocial, como le quieran llamar aquí no hay instituciones públicas que lo trabajen de manera concreta, entonces al tener recursos reales de dar respuesta más que un asistencialismo, porque el programa ofrece más que eso, se estaría dando respuesta a nivel nacional de la situación de personas en esta condición porque yo no me apego mucho a los conceptos de enfermedad, sino que son condiciones individuales que nos hacen. Pero el modelo, si el Ministerio de salud quisiera adoptar esto como un programa interno, dieran una respuesta masiva a la sociedad, pero eso lo tenemos que impulsar, eso es lo que yo veo.
- 4: yo le iba a decir que entiendo el nivel nacional como poner el programa aquí en El Salvador a través de ACISAM, AFAPIM, entiendo que dentro del estudio está el abrir más programas así en todo Centroamérica, porque el problema de la salud mental es bastante fuerte y este tipo de programas no es el único porque también está el Hospital de día del Psiquiátrico… pero así como este tipo de ACISAM, creo que es el único que hay y ayuda mucho, porque uno pasa ocupado porque afuera no encuentra un trabajo y ACISAM le brinda la oportunidad a uno de estar entretenido y productivo, entonces a nivel nacional hay este problema de salud mental.
- 5: a nivel nacional lo positivo es que por medio de las radios se puede divulgar a nivel nacional.
- 7: para mí a nivel nacional el impacto del programa es que tendríamos una sociedad más educada sobre las enfermedades mentales y también una sociedad menos estigmatizante.
- 2: pero ahorita la idea es recoger lo que el programa ha hecho, ha impactado a nivel nacional, esa es como una proyección.
- 1: bueno ella dijo que es más educada, y ya tenemos más de 10 años de correr con este programa, la pregunta es ¿la sociedad es más educada o no?
- 7: quienes hemos participado en el programa sí, pero no podemos hablar de que esto ha abarcado a nivel nacional porque estaríamos mintiendo, no se ha logrado una cobertura a nivel nacional porque faltan los recursos, sin embargo las necesidades existen aquí y en todos los departamentos del país, pero la familias que hemos participado en el programa, definitivamente estamos más educadas.
- 6: para mí lo nacional es que nos dirán más educación para los enfermos como nosotros, que nos dieran para trabajar, estudiar, algo así….
- 3: hablaré un poco de números, en el 2007 se hizo un estudio centroamericano, no incluía Panamá ni Honduras, en donde decían “¿cuántos hospitales? Tantos”, “¿cuántos psiquiatras? Tantos”, “¿cuántas organizaciones? Cero!” yo pienso que si volvieran a hacer ese estudio, saliéramos identificados, si bien es cierto que el Estado, el Ministerio no nos apoya, habría que ver la perspectiva de ellos, pero hoy sí borramos ese cero de una encuesta, existen organizaciones de familiares contra la defensoría de los derechos, con capacitaciones, con actividades comunitarias, apareciéramos que si tenemos, entonces pienso que a nivel nacional de una u otra forma hemos logrado un avance, y de hecho, si seguimos organizados pensamos que para lograr más cosas a nivel nacional, no solo para nuestro pequeño grupo, sino para todos aquellos que no nos han llegado a conocer, entonces quitamos el cero de las estadísticas y ya pasmos a formar parte de ese uno que tendríamos que poner.
- 1: yo pienso primeramente en los foros, porque algunas eran personas del Hospital Psiquiátrico, Policía Nacional Civil también Escalante de salud mental por el gobierno, y esta era una manera para nosotros de empezar a establecernos como entidad de salud mental en el país, y empezar a dar educación a estas diferentes instituciones sobre el papel de la familia y el derecho de los usuarios, también recientemente nuestro trabajo con la policía, es un grupo pequeño pero tienen jefes, y los jefes bien animados van a hablar con sus jefes, y estos jefes tienen conexiones con la Asamblea y diferentes ministerios y no podemos conocer todo lo que está pasando, pero con este pequeño esfuerzo tenemos más y más efecto, creo que es positivo, también estamos abriendo puertas en estas instituciones para seguir el programa.
- 10: en la línea que dice “1”, uno de las participaciones con mayor incidencia política es desde el CONAIPD, porque ahí tenemos incidencia en seis ministerios, una secretaría de Estado y otras instituciones, lo que yo quiero decir es que todo programa tiene proyecciones, quizá habría que trabajar un poco las proyecciones ¿por qué? Porque como nosotros somos pioneros, ahora si hay asociaciones de familias y personas con discapacidad, el país va en una evolución en el tema de la discapacidad ¿qué pasa cuando quieren expertos? Ese es el concepto que utilizan ellos para personas con discapacidad mental o psicosocial, tenemos que prepararnos porque la presión del impacto nacional nos va a empezar a llamar y hay cosas que no le hemos dado respuesta aún.
- 2: yo quiero decir algo también, y es un impacto bien fuerte del programa, que el programa ha facilitado que la población vea a las asociaciones de familiares y usuarios como referentes y defensoras del tema de discapacidad mental, enfermedad mental y derechos humanos, y eso si es importante, ya hay un trabajo hecho y las dos asociaciones ahora son más conocidas que hace dos años, ahora nos conocen a nivel nacional.
- 7: el trabajo que se ha hecho en los medios de comunicación también ha impactado porque ese es el único medio que por el momento tenemos de abarcar y llegar a nivel nacional y dar a conocer el programa y la asociación.
- 2: y de medios estamos hablando de radio, televisión y hasta la prensa porque también hemos salido en unos artículos del Co-Latino.
- 1: vamos a seguir con la nueva pregunta, ¿cree usted que son importantes las organizaciones familiares como ASFAE, AFAPDIM, Cuenta Conmigo en Nicaragua? ¿cuáles son las funciones, ¿cuáles son los beneficios de las organizaciones de la sociedad civil para lograr el bienestar de salud mental?. Entonces la primera parte de esta pregunta es ¿cree usted que son importantes las organizaciones de salud mental como ASFAE, AFAPDIM, Cuenta Conmigo en Nicaragua? Creo que todos piensan que son importantes.
- (al unísono, todos contestan sí)
- 1: entonces ¿cuáles son las funciones de este tipo de organizaciones?
- 4: primero es atender a personas con problemas de salud mental y enfocarlas a un empoderamiento, a una mejor calidad de vida y a tratar de mejorar su salud mental.
- 1: excelente. ¿quién más? Funciones importantes de este tipo de organización.
- 7: para mí una de las principales es la defensa de los derechos de las personas con enfermedades mentales, la sensibilización hacia las demás personas y especialmente a instituciones, por ejemplo hace poco se estuvo capacitando al personal de la PNC y el resultado es que los jefes ahora son más comprensivos con el personal y ya no tienen tanto peros a la hora de otorgar permisos para las citas, porque a veces son un poco frecuentes, y entre otras cosas la dinámica de trabajo de ellos.
- 5: para mí que es visibilizar la enfermedad, porque estas organizaciones no ponen a las instituciones como primero y al paciente como después, sino que primero ponen al paciente y que sin el paciente no existen las instituciones.
- 2: “5”, ¿le ayudo para entenderle?
- 5: sí.
- 2: primero se visualiza a la persona.
- 5: sí, pero a veces siento que el crédito se lo lleva la institución, de que todo lo que se hace, lo hace la institución, pero no, también el familiar pone de su parte para empujar y visibilizar.
- 1: otra parte de esta pregunta es ¿cuáles son los beneficios de las organizaciones de la sociedad civil como estas organizaciones, AFAPDIM, Cuenta Conmigo, ASFAE? Entonces hemos discutido ayer bienestar de salud mental y qué hacen estas organizaciones para lograr este bienestar.
- 2: yo quiero poner dentro de la mesa un punto que me parece bien estratégico, dentro del programa intencionados la educación entre pares, lo que nosotros le llamamos la psi coeducación y autoayuda, entonces una de las funciones y beneficios que debe de dar las asociaciones de familiares es la psicoeducación y autoayuda, que no es lo mismo que dentro del programa hace cinco años los que facilitaban los talleres de familia a familia era “3”, a que ahora lo facilitan los familiares, eso es un gran avance.
- 10: pero eso siempre ha estado, yo recuerdo que desde el 2004 los familiares capacitaban los cursos.
- 1: pero también con el grupo de arte terapia, empezó con profesionales y ahora con familiares, creo que es un proceso que hemos cruzado poco a poco, también con las visitas domiciliares, primeramente profesionales, ahora “3” dijo el otro día que están involucrados los familiares, y esto ayuda mucho porque pueden conectar muy bien con la familia, no es como solamente “doctor o doctora, licenciado”, no es como “soy familiar también, habla conmigo”. Bueno, realmente creo que hemos respondido de diferentes maneras a esta pregunta, entonces vamos a seguir a la próxima. ¿Cómo el programa nuestro se compara con otros servicios y programas disponibles a los usuarios/as y sus familiares?, ¿existen otros programas?, ¿Cómo comparan?
- 4: solo el Hospital de día, más o menos, pero ahí es de lunes a viernes y éste es casi solo los sábados, un día a la semana, pero este tipo de programa de ACISAM es único en el país, desde la ayuda económica que recibimos hasta el tipo de talleres y todas las actividades, todo lo que programan durante el año, porque el Hospital de día no los llevan a pasear o no hay salidas, no ven películas, entonces prácticamente este programa de salud mental es único en El Salvador, difícilmente se le puede comparar si es que hay otros programas en el país.
- 8: si es para la persona cuidadora, desde mi experiencia no encuentro otra, prácticamente creo que solo éste es el que se encarga de educar al familiar,
- 7: yo tampoco conozco a ningún otro programa igual, ni he oído.
- 10: yo he escuchado que dicen, que en el seguro social dan alguna atención a los familiares, pero del decir, yo personalmente no he averiguado si lo dan o no, pero yo sí tengo plena seguridad que programas con un enfoque de apoyo al cuidador en este tipo de condiciones, es único.
- 6: yo he oído que en el Hospital Psiquiátrico están dando así como nosotros hacemos, a mí me dijo una doctora que hacen grupos para autoayudar a las demás personas, la psicóloga del hospital me dijo que si yo me podía reunir y le dije que no porque yo estoy en otro grupo.
- 1: ¿ella conocía a ACISAM o no?
- 6: no, solo un doctor que conozco el Dr. “C”.
- 3: bueno, yo la experiencia que tengo con otros grupos, y quizá suena risible es que cuando yo identifico a ASFAE, yo llamo a ASFAE, doy mi currículum y me dicen “no, usted no puede participar porque no tiene familiar y es profesional”, me cerraron las puertas así de fácil, entonces para mí es muy difícil tratar de ver cómo podemos ayudar, de hecho, yo expliqué que quería ser voluntaria, yo no voy a cobrar, yo solo quiero participar, y me dijeron que no porque no era ni familiar ni paciente con trastorno mentales. Después conocí a ACISAM, que fue todo lo contrario, se me abrieron las puertas, me dieron oportunidad y lo poco que conozco actualmente del Hospital Psiquiátrico, es que Hospital de día es como venir a trabajar haciendo arte, pero quizá con la visión de ayudar económicamente al usuario, y muchas veces el usuario lo que busca es una ayuda económica, “yo produzco y tú me ayudas a vender” y obtiene una ganancia, mientras que el Hospital de día, pienso que no es esa su perspectiva sino la terapia ocupacional y del seguro no se nada.
- 1: ¿y terapia ocupacional significa manualidades?
- 3: sí.
- 1: muy bien, seguimos a la próxima pregunta son dos sobre liderazgo, ¿cuán importante es el liderazgo en estas organizaciones para alcanzar sus metas y dar satisfacción a sus miembros?
- 4: yo puedo contestar. Por ejemplo, “N”, “C”, “H” con los talleres de arte terapia, “C” con las dinámicas y los talleres psicoeducativos, “N” en la organización y el Programa, usted en los foros, ayuda económica, se coordina con el señor “D”, yo pienso que sí es súper importante, porque depende de cómo organicen lo que se harán cada sábado durante el año, “M” también, así es como funciona el programa, porque el liderazgo es súper importante, porque si no fuera por los que están al frente de la organización, no habría ACISAM, o se coordinaría muy mal, no hubiéramos durado cinco años.
- 8: yo pienso que el liderazgo es importante en las organizaciones, porque solo a través de éste es como se puede hacer ver los problemas, no solo a nivel individual sino también de una colectividad que está atrás de una líder o de un líder, entonces una organización si no tiene líderes conscientes de lo que están haciendo, nunca se van a visibilizar, nunca van a tener voz.
- 1: ¿qué es un líder consciente?
- 8: me refiero a que sepa por qué está haciendo las cosas, aparte de tener sensibilidad, tenga cierto grado de coherencia con el discurso, alguien podría ser demasiado hablador, pero si no es coherente con lo que dice y hace de nada sirve.
- 5: a mí me parece que el liderazgo es importante, siempre y cuando sea compartido, porque hay un tipo de líder que más que todo es protagónico, que cuando la cosa está bonita, si representa al grupo, pero cuando la cosa se pone fea se esconde, entonces para mí el liderazgo tiene que ser compartido, bueno yo veo que “2” siempre está en las buenas y en las malas, y delega, no es él el que protagoniza todo, como si no estuviera él no funcionaría esto, sino que lo comparte entre el grupo y siempre es incluyente.
- 1: incluyente es como que incluye a todos y quiere la participación de todos.
- 5: le da participación a los demás.
- 3: yo hablaré en el sentido de tener algo muy claro “S”. Yo veo un liderazgo muy diferente de estas organizaciones a un liderazgo institucional, porque cuando uno es profesional le ponen una meta, por ponerles un ejemplo “tienes que hacer cuatro grupos comunitarios”, bien yo voy a cumplir con mi meta; mientras que el liderazgo que se desarrolla en estas organizaciones es protagónico, y no es en función de cumplir una meta, sino que en función de defender los derechos, en función de velar por el bienestar de la salud mental, mientras que muchas veces nosotros como profesionales o como entidades públicas hacemos un liderazgo por compromiso, entonces confío más en este tipo de liderazgo porque sé que no va a fallar porque son usuarios, son familiares y el protagonismo va a ser siempre.
- 1: entonces porque no es pagado, es hecho por compromiso, no tiene que tener un estilo de liderazgo diferente, y también la idea es para fomentar trabajo como grupo y no solamente por cumplir metas.
- 7: para mí el liderazgo es una de las partes fundamentales y claves del éxito de las asociaciones, porque no solo se trata de ser líder, sino también de que ese liderazgo sea unificador, y que todo lo que se hace sea en función de la visón y misión de la organización o entidad con la que se está asociada.
- 1: la segunda pregunta sobre el liderazgo es ¿Cómo estas organizaciones ayudan a formar y a desarrollar líderes y habilidades de liderazgo entre los participantes?
- 5: yo solo veo que es delegar responsabilidades, si la persona cumple con su responsabilidad y se ve que le va poniendo amor a lo que hace, pueda que se convierta después en un líder.
- 7: yo apoyo la noción de “5”, creo que es bien importante identificar y reconocer las habilidades y capacidades de cada persona, y así asignar responsabilidades y tareas para que sean compartidas. Voy a hablar de mi caso personal, yo no tenía idea de tener tanta habilidad para las dinámicas, que es una cosita pequeña pero ahora yo me sorprendo, porque tampoco había explotado esa parte que hay en mí y que disfruto y me gusta y lo descubrí aquí en la Asociación…
- 10: es promover el descubrir las habilidades que tenemos ocultas, eso es promover las habilidades que a través de la delegación, que muy oportuno lo que decía “5”, y la renovación, en el sentido de que hay unos que tienen esa gran capacidad de liderazgo pero nunca se atreven a ir y representar, porque a veces en el liderazgo hay voceros, por eso es que hay líderes, hay una vocería, una representación, pero hay que ir renovando con los miembros para que todos seamos líderes de algo.
- 8: también la motivación es muy importante, motivar a los asociados y asociados a que esas habilidades que tienen se exploten por todos los medios, porque tampoco de nada sirve que uno delegue responsabilidades si uno no ve cierto estímulo, y no necesariamente en lo económico, pero podría ser un reconocimiento o algo así, un diploma.
- 3: algo muy importante es que aquí no nos piden un título para servir, un ejemplo es Niña “M”, ella llegó a mi consulta como usuaria, tenía sus habilidades y ahora es una de las que más participa, y es un líder dentro de su área, de las iniciativas económicas, yo le digo a “2” que esa mujer tiene la capacidad de ver el mercado, que muy profesional puedo ser yo pero no trasciendo, no tendría la capacidad, como voy a ir a vender yo bolsos, como iría a vender bisutería, entonces siento que está institución no me pide un título, sino que dentro de nosotros mismos nos descubrimos y nos dan el espacio para ser libres.
- 10: esque se promueve la diversidad de liderazgo, nosotros no tenemos el perfil como alguna gente, el que habla más, el que tiene expresiones ligeras, sino que resaltamos las cualidades, las ubicamos, pero hay quienes, por ejemplo aquí “5” ya ha sido el que ha enseñado a hacer hamacas, porque tiene ese liderazgo, entonces promover esa diversidad de liderazgo.
- 4: dentro del liderazgo también está crear uno su propio negocio a través de las artesanías y de lo que se aprende en ACISAM, una persona podría hacer sus artesanías con algún capital que se tenga, como un medio para ganarse la vida…
- 1: disculpa, vamos a seguir con nuestro último grupo de preguntas, solamente dos preguntas más. Este es un nuevo rumbo, vamos a discutir un concepto que se llama capital social, voy a explicar un poco de este concepto de capital social; social se refiere a las relaciones entre la gente, y capital es un concepto del mundo de finanzas en que uno tiene un recurso para hacer su trabajo, pero hay recursos no solamente material y financiero, también hay recursos de relaciones, hay recursos sociales, entonces estamos hablando más que todo de la idea de qué recursos, qué cosas tenemos como una fuerza social como grupo, y hay dos grupos de capital social, uno se llama capital vínculo, es decir conexiones dentro de un grupo, y el otro es capital social o capital puente, es decir capital que construimos entre grupos, afuera de nuestro grupo con otros grupos como redes, entonces capital vínculo: se da cuando la persona socializa con otros semejantes dentro del grupo. Capital puente: el puente se da cuando se crean lazos con puentes distintos. Bueno, no sé si más o menos entienden este concepto o tienen preguntas sobre qué es capital social.
- 7: el primero es entre grupos y el otro fuera del grupo.
- 1: o entre grupos, por ejemplo todos nosotros tenemos interés de trabajar y ayudar a familiares y usuarios, entonces somos semejantes, tenemos vínculos juntos y tenemos intereses comunes y así trabajamos juntos, con el tiempo formamos relaciones, nos comunicamos, trabajamos juntos, planifican, llevan servicios y todo eso, y esto sirve para hacer más y más fuerte nuestro capital vínculo, y si queremos seguir adelante y alcanzar un mejor nivel de nuestro trabajo, no podemos seguir como grupo de individuos, tenemos que relacionarnos con otros grupos, tenemos que cruzar puentes, por ejemplo la Policía, es muy diferente que ACISAM la policía, o el Hospital Psiquiátrico tienen una manera bien diferente de trabajar que nosotros, pero queremos formar un puente hacia ellos y a la policía y a la gente de CONAIPD, queremos formar estos puentes para que podamos tener un capital social en un nivel más grande. Entonces estos son los conceptos de capital social, y las preguntas son: ¿Cómo el programa nos ha ayudado a crear mayor capital social para individuos y nuestras organizaciones?, voy a repetir: ¿Cómo el programa nos ha ayudado a crear mayor capital social para nosotros como individuos y para nuestro grupo o nuestra organización?
- 6: el capital del grupo de arte, todo el grupo somos bien unidos pero a veces cuando estamos trabajando en todo el trabajo que hacemos en arte nos sentimos bien halagados, porque a veces nos ponemos a chistar, nos ponemos a hacer cosas muy interesantes, a veces a bailar, y nos divertimos, porque solo estar trabajo y trabajo nos aburrimos, todo el tiempo que hacemos arte terapia también necesitamos tener un pequeño espacio para divertirnos un poco.
- 4: nos ha ayudado a socializarnos, a platicar un poquito con las demás personas, a ser un poco más amable, a no fingir lo que uno no es sino que ser uno mismo, a hacer amistad, y cuando vemos que no somos los únicos en tener este tipo de problemas uno ya puede ver que hay personas que también tienen este problema de salud mental. Ahora, con otros grupos casi no hemos tenido contacto, pero sabemos que hay otros grupos de personas…pero con las estudiantes que vinieron se pudo sociabilizar y ese capital vínculo, pudimos conocernos un poquito más, y en puente estamos un poquito mal porque no interrelacionamos con otros grupos que tienen este problema de salud mental.
- 1: antes de seguir voy a hacer más sencilla la pregunta, para tener éxito uno en el programa, como el nuestro, necesitamos relaciones, más que todo relaciones de confianza, cuando los investigadores están midiendo a capital social, casi siempre se enfocan en la confianza, entonces nuestro programa está formando relaciones de confianza entre nuestro grupo y también afuera, y la pregunta es cómo, cómo son nuestros esfuerzos creando relaciones de confianza dentro de nuestro grupo y afuera con otros grupos, cómo.
- 4: primero con el vínculo entre nosotros de amistad, respeto y compañerismo, y con otros grupos respetando a los demás y tratando la forma de ser amable con las demás personas.
- 5: para mí que AFAPDIM y ACISAM ha alcanzado un nivel de convocatoria bastante alto, porque me he fijado que muchas personas conocen el programa y han estado en diferentes etapas con AFAPDIM y ACISAM, porque una gota cayendo y perseverando sobre una piedra, al final la rompe, y las personas a veces no quieren, pero yo me doy cuenta cuando son las asambleas que llegan bastantes personas y se ve que hay bastante capital social, y mucha gente de esa tiene confianza porque cuentan qué es lo que está pasando afuera, qué dicen de ACISAM y AFAPDIM, y esas personas son las que mueven a que los demás tengan confianza y se salga afuera, con el trabajo que hace AFAPDIM y ACISAM, es su carta de presentación para tener un capital social fuerte.
- 8: yo recuerdo que cuando entre a recibir el taller, lo primero que se establecen son normas de convivencia, entonces esas normas de convivencia como nacen de todas las personas que están participando ahí, es lo que permite mantener las relaciones entre los familiares que asisten, yo creería que el capital social crece en la medida que se establecen normas de convivencia que nacen de todos.
- 10: a nivel interno pienso que se generan grandes lazos de confianza y de familia, a nivel externo, por ejemplo cuando uno llega y dice “venimos de ACISAM y AFAPDIM”, hay un respaldo porque se identifica a ACISAM y eso promueve un nivel de confianza, yo y la experiencia en convocatorias por ejemplo, de la convocatoria en foros y en otras actividades de profesionales claves en el país que inciden bien o mal en el país, ellos sabrán, siempre hay apertura, por ejemplo el Dr. “C”, la Dra. “B”, ellos son directores de los hospitales aquí, entonces hay apertura, significa que hay confianza del trabajo que se está haciendo…
- 5: será que AFAPDIM y ACISAM hace trabajo de hormiga a hormiga?
- 1: ¿piensan ustedes que necesitamos crear más capital social?
- 8: sí
- 10: eso sí que da poder de convocatoria, poder social, vean otras organizaciones que tiene más de 3000 afiliados. Yo lo he percibido por ejemplo cuando ALGES está sentado en una mesa es una cosa diferente, porque tienen más de 10,000 afiliados, entonces ahí hay un movimiento social, pero yo me pregunto qué pasaría si aquí nos sentamos otra organización que tiene la capacidad de mover a 200 mil personas…
- 1: pregunta qué necesitamos para crear más capital social dentro y fuera del grupo.
- 2: quizá si hacemos una evaluación de los diez años del programa y cuánto capital ha creado, creo que el número es cada uno de nosotros, y ahora que estamos en red, quiere decir que ahora, que es una suma de 12 años lo podemos hacer en tres años, porque ahora ya estamos más conectados, y para aumentar este capital vínculo tenemos que lanzarnos a nivel comunitario, porque no se trata de que las personas vengan a recibir el servicio de salud mental y educación, sino que nosotros llevar el servicio, en ese sentido lo multiplicaríamos rápido, pero para eso se necesita inyectar recursos, y a nivel del capital puente cuál es ahorita nuestra apuesta? Nuestra apuesta es la red regional, y en capital puente estamos en la Alianza, CONAIPD, que no son organizaciones que hacen lo mismo, sino que hacen cosas diferentes, entonces profesionales, sí los necesitamos, creo que este programa ha alcanzado tres niveles y hemos alcanzado un nivel de éxito con los profesionales primero, combinación de profesionales con familiares, y ahora parece que está despegando más familiares, con un perfil más bajo del profesional.
- 3: me gustaría hacer un enfoque de otra perspectiva, no olvidar los números, porque acordémonos cómo nos miden por pobreza: cuántas muertes por parto, cuántas muertes infantiles, cuántos niños vacunados, pero aquí hay una alta tasa de suicidios y no se investiga, hace poco me preguntó que si las posiciones de directores o ministro tiene que ver con la política, sí tiene que ver mucho con la política, y si no, tomemos las palabras de “10” cuando dice “el Dr. “E”, nos trasladamos a 1972” o sea que lejos de avanzar, retrocedemos. Para mi “1” tiene que ver mucho la legalidad, ¿en qué sentido? Cuando nosotros somos un ente legal yo siempre le he apostado ir a pedir algo de beneficio, pero si no estoy legal, cómo toco la puerta, yo no quiero que perdamos esta perspectiva de “hay sí que chivo que estamos en la mesa”, incluso, esas participaciones muchas veces son muy individuales, yo le apostaría más “1”, primero siendo legal, segundo, fíjense que yo a veces no pretendo sensibilizar al papá o al hermano del esquizofrénico, sino que sensibilizar al profesional, cuántos psiquiatras tenemos, y cuántos les mandaron del psiquiátrico, aquí si vamos a hablar hay que hablar la realidad, le digo porque hay cosas que no han funcionado, pero por qué no han funcionado “1”, esa es la pregunta que hay que hacerse, muchas veces digo vamos a sensibilizar a “10”, a “8”, que han vivido con su familiar? Pero qué sucede con el profesional? doy las recetas y doy fecha para verlo en seis meses, y cuando llegan me ponen un montón de expedientes que tengo que ver en tantas horas, entonces miden la cantidad no la calidad, entonces deberíamos empezar desde arriba a sensibilizar, por ejemplo cuando fuimos con la policía me dice “2” es un grupo muy cerrado y todas las características de este tipo de gente, pero qué pasó? Cuando empezamos a hablar del tipo de patologías se mostraron muy abiertos, y algo muy importante “1” cada homicidio aquí conlleva a un duelo, conlleva a un duelo no resuelto, ¿le importa eso al gobierno? No! A ellos les interesa los municipios libres de violencia entre comillas, yo le apostaría más sensibilizar a nuestro recurso, que no pierdan la perspectiva humanitaria…
- 1: quiero agradecerles a todos y realmente ha sido muy interesante para mí y yo he aprendido mucho de ustedes y especialmente quiero agradecer a “6”, a “4” y a “5” porque ustedes tienen esta experiencia muy personal y pueden comunicarse muy bien sobre estas cosas, es una contribución muy valiosa, gracias a todos.
