# Supplementary material for: A qualitative exploration of a family self-help mental health program in El Salvador
Source: Int J Ment Health Syst. 2016 Apr 1;10:26. doi: 10.1186/s13033-016-0058-6 (PMC4818454; doi:10.1186/s13033-016-0058-6)
Supplement: Supplementary file 4 — 10.1186/s13033-016-0058-6 Focus group questions and summary of responses. [file 13033_2016_58_MOESM4_ESM.docx]

**Additional File 4**

**Focus group questions and summary of responses**

**Question 1.** In response to the question, “Do you believe that users and caregivers who participate in the FESEP program have better mental health wellbeing than those that do not participate?” all in the focus group responded Yes. Focus group participants discussed at length what constitutes mental health wellbeing. We explore this discussion of mental health wellbeing in another paper. For the purposes of this paper, there was sufficient similarity across all three subgroups to indicate that we had a common understanding of the meaning of mental health wellbeing.

We then asked why they thought program participants had better wellbeing. Users responded most extensively to this question. They felt user program participants were more productive and occupied, not trapped in their home angry and tense, in a space where they can work on their mental health, and belonged to a social group where there is understanding. Furthermore, they felt that the more someone participates, the more they benefit from the program. Some are able to earn enough money to survive independently. Within the Salvadoran society there are no governmental or nonprofit community mental health programs aside from the FESEP program. Thus the opportunity for people with mental illness to be able to leave their homes and have a place to go (other than the psychiatric outpatient day program, located in the national psychiatric hospital) was prominent in their comments.

**Question 2.** In response to the question, “What are the most significant changes that you have seen in others resulting from the program?” focus group members’ responses fell into four categories.

***Feelings.*** Program participants were happier with their own lives and with others around them. They often felt free of stress, unburdened, and connected. They could express themselves and talk about their illnesses (which they often cannot do outside the safety of the group), and they feel as though they have a role.

***Behavior.*** Program participants have had others say to them that they have seen dramatic changes in the life of the person. Participants stated they were more optimistic, participative, and social. One caretaker said “Before she was like a little island, but now she can open herself up to others.”

***Attitude and Thinking.*** Progressive understanding and improvement was a consistent theme. “One program participant still cannot accept that he is ill, but he is now able to accept that he has a problem.” Some participants have progressed to the point of being conscious of their human rights as persons with mental disabilities.

***Responsibility.*** Program participants felt useful and engaged. “Before, the professional volunteers carried a huge burden for the program, but now the families are in charge of a lot. For example, they accompany the professionals on home visits, and this has completely changed our interaction with families in crisis because they are peers and they can create a higher level of trust with the families than we can as professionals.” Family leaders have assumed the teaching of educational classes. They have taken on the challenge of forming their organization into a government-approved nonprofit organization, a process that generally takes several years in El Salvador and is very costly for low-income groups. Families have learned how to share the burden of care in their family. Regarding taking advantage of the opportunity for work, “For people with disabilities, employment is really difficult to obtain, but thanks to [the program] there is a place to go and …learn skills.”

Several respondents suggested that users and families are assuming increasing responsibility because they are so thankful to the program. They stated that before the program they felt lost and alone, but now some participants have become highly involved.

**Questions 3.** In response to the question, “Why do some family caregivers benefit more from the FESEP program than others?” focus group members identified barriers caregivers often experience to program participation. One barrier was compliance with taking medication. When this barrier was overcome in a family it allowed family members to more easily participate because the family was not in a crisis all the time. Other barriers included money, the ability of a family member to transport or accompany a user on public transport to the program, the distance from meetings, and fear (of leaving the home, of public transportation, of going to a new place, of the program, of interacting socially with others, of being in public with one’s illness, etc.). Some family caregivers had to have someone to watch their ill relative so they could leave. This is especially hard on single mothers with multiple disabled children who want to participate in the program. Finally, some families (especially those with years of adjusting to the illness) felt out of their comfort zone and were unwilling to change or push for change in the family or with the user, which is what program participation helps families to do. It’s easier to just keep the ill relative housed all the time.

It was stated that wealthier families who can afford regular expensive psychotropic medications may experience greater stability and thus not feel that they have as much need for support and education as poorer families.

The focus group members stated that some families have a better understanding than others of the potential benefits of participating in a program, including intangible benefits such as education. They believed in peer education, which has benefits such as empowering people to become leaders in their neighborhoods and in the FESEP program, offering free education and support to other families, and providing opportunities to families and users to advocate at the highest levels for their rights.

Some families had inappropriate expectations of the program, thinking it would provide them desperately needed medications, or personalized treatment (rather than peer support), or professional clinical treatment in the community (rather than psychiatric visits in the national hospital, where most care in El Salvador is provided). Some families came hoping for miracles rather than long-term solutions that requires engagement in the program.

On the other hand, some families took advantage of all the supports available - professional support, medication, program support, and education groups - perhaps because they had a higher level of trust between the family members and the user. Some families had a higher level of attendance consistency, a higher level of commitment, or a higher ability to manage the give and take of group dynamics. All of these characteristics were felt to add to the ability of families to participate over the long-term in the FESEP program.

**Question 4.** In response to the question, “Why do some users benefit more from the FESEP program than others?” focus group members’ responses were somewhat similar to the responses for families, but emphasized more the personal supports for the user. Some users had spiritual support, professional help, and the support of their families. Focus group members said it is very typical that a caregiver participates first in the FESEP program and then brings the user when he/she is no longer in crisis.

Sometimes there is disagreement over the person’s diagnosis, and this works against the person having clarity about the need to participate in the program. Focus group members felt that sometimes expectations were not met, such as when a user wanted to get money for meds, or to find a girlfriend.

Some users had more personal interest or motivation to participate and they involved themselves, while others are more comfortable staying home. “My son just doesn’t have the strength of will to participate.”

However, some could not participate simply because they didn’t have the resources, or the program was very far from where they live.^[[1]](#endnote-1)^

**Question 5.** In response to the question, “What differences between families could influence the situation in a way that families are helped by the Program?” Focus group members listed education level, economic conditions, family composition (one caregiver or more than one user), and family level of interest and commitment toward the user, or, as one user said, “The level of love the family has toward the user.”

We discussed duration of illness as a potential influence on program participation. People felt that if families had received the training earlier, and received earlier intervention, there could very likely have been a reduction in the chronic severity of the illnesses.

Regarding the attitude of the family toward the user, everyone felt it was a very important influence. Where the family is situated in the history of the illness (stage or phase) is important too. “Families that are new to the situation cry out ‘help us!’” But families that have been managing the illness for years have adjusted to the situation and are not so anxious for help. “The first crisis is the best time to work with families. Early intervention is more efficient in the long run.”

**Question 6.** In response to the question, “Is the program positive?” focus group members all responded yes. Researchers then asked how the program was positive at different social levels: individual, family, community, organizational, and national (across society). Focus group members provided extensive responses regarding the positive effects of the FESEP program at each social level.

Regarding the individual level of social interaction, users and family caregivers identified numerous personal benefits (Figure 3). These contrasted significantly between users, family members and professionals, which reflects how the program has identified and met their diverse needs. Respondents did have common benefits such as gaining a larger perspective, being committed to a larger cause, national advocacy, and discovering each has a useful role in serving others. That is, they found meaning in connecting themselves to the larger needs of others and of society.

User program benefits concerned being active and engaged, receiving attention and understanding regarding the illness, and increased self-esteem. Family benefits focused on reduced burden, increased support and understanding, and assistance in ongoing times of crisis.

In responses related to the family level of social interaction, user comments reflected the two-way benefits of the program: their behavior was improved and reduced problems with the family, but family behavior toward the user was also improved. Users were even empowered to help educate and sensitize non-ill family members (Figure 4).

Likewise, family members reflected the two-way benefits of the program from their perspective. Family caregivers noted secondary or indirect benefits of the program such as improving overall family communication, unifying the family, and applying what they learned to other disabled (non-mentally ill) family members. Finally, grasping concepts like user dependency on family members and caregiver burnout led to improved ways of managing those challenges for family members.

One professional noted how volunteering in the program has raised the consciousness of her children. Through observing her they gained an understanding of mental illness and now strongly support her commitment of time to her volunteer work.

In responses related to the community level of social interaction, users, family caregivers, and professionals clearly defined benefits according to their position in the program. Users were able to relate better to others in their communities, to develop friendships, and to share about their lives more fully with others. Users, in turn, received encouraging feedback from people in their communities that helps to improve their self-concepts (Figure 5).

Family members talked about “before and after” participating in the program. Before participating there was fear, poor communication, and ignorance. Afterwards there was understanding, insight, ability to overcome stigma and talk with others about mental illness, empathy for other families in the neighborhood, increased relational skills, and empowerment to advocate for needed changes.

For professionals the program facilitated work in dangerous communities. It also opened professionals to the idea of working at the community level because they experienced and saw the need, the opportunities, and the benefits of working inside communities and inside people’s homes.

In responses related to the organizational level of social interaction, everyone felt the organization empowered its members to advocate and facilitated that work through organizational structures and collaboration with outside groups. They felt an identity through the organization and a sense of common purpose (Figure 6).

In responses related to the national or society-wide level of social interaction, participants focused on four areas. Positives of the program related to national advocacy included the program filling a unique role, gaining a strong national and international reputation, and the opportunity to educate and sensitize other national NGOs about mental illness and mental health rights. Advocacy activities directed toward overcoming stigma have given program participants the sense that they are changing society and reducing stigma against those with mental illness (Figure 7). Program participants felt they learned to think big, for example, how to expand mental health services in El Salvador and how to support user and family groups in other Central American countries.

**Question 7.** In response to the question, “Do you believe that family organizations [in Central America] like ASFAE, AFAPDIM and Cuenta Conmigo are important?” all focus group members said yes. We followed up by asking what are the functions of such organizations.

Functions fell into four areas. 1) Service to individuals – attending persons with mental illnesses and empowering them to achieve a better quality of life. 2) Advocacy – in defense of human rights for those with disabilities and helping to give visibility to the issue of mental illness while keeping the primacy of each user’s personhood foremost in mind. 3) Sensitization—anti-stigma awareness raising of other individuals and institutions that impact users and family members such as neighbors, the police, hospital personnel, and mental health professionals who work with families. 4) Promotion and use of the methodology of empowerment – promoting peer education, group psychoeducation, volunteerism, and self-help as a complement to professional help.

**Question 8.** In response to the question, “How does the FESEP program compare with other services and programs available to users and/or their families?” focus group members stated that the national psychiatric hospital day-program supposedly serves users and families, but they did not feel the program was significantly helpful.

One other family program exists in El Salvador, which started about the same time as the FESEP program. However, it serves only family members and excludes users and professional volunteers. This program does not have a structured curriculum and is limited in its advocacy and other activities. One professional felt badly at not being allowed to participate in (help) this other program.^[[2]](#endnote-2)^

**Question 9.** In response to the question, “How important is leadership in these organizations for achieving their goals and providing satisfaction to their members?” focus group members agreed that leadership is fundamentally important to a well functioning and successful organization. Leadership helps to resolve problems, lead planning, and create the unity necessary to do the work, thus allowing an organization to endure.

They cautioned it must be quality leadership, by which they meant leading for the right reasons, which include serving the mission and the people, the opposite of egotism. Leaders must have sensitivity, clarity, and coherence. Focus group members believed that good leaders share leadership and are role models to others.

Focus group members stated leaders play an important role in helping the group find its voice by guiding program participants. Leaders coordinate and help people to share the work and apply outside concepts internally in terms of rights and respect for those with mental illnesses. Leaders of FESEP help to unify everyone around the mission and larger vision.

Focus group members noted differences between the public and nonprofit sectors. They stated that an NGO leader is a protagonist, facilitates the work of the group, and does his or her work out of commitment, not just for pay; whereas, in governmental institutions the leadership is vertical, you do what the boss says and your work is based on goals passed down from above.^[[3]](#endnote-3)^

**Question 10.** In response to the question, “How do these organizations help to form and develop leaders and leadership skills among their participants?” focus group members discussed their own experiences, reflecting on their self-discovery of leadership capabilities, the process of nurturing leadership development, and the idea that anyone can and should be a leader. Specifically they said,

- “We discovered leadership abilities because we were given the opportunity to develop our leadership through the group.”
- “I had no idea that I had such a great ability or how much I would enjoy facilitating groups and leading mixer activities.”
- “We develop leadership by delegating responsibilities according to our knowledge of the abilities of each participant. We don’t ask that a person have a title or educational degree to be a leader; we open spaces to anyone who is participating to be a leader.”
- “We promote the diversity of leadership” [across users and caregivers, high and low educational levels, wealthy and poor people].

**Question 11.** In preparation for the discussion of social capital, researchers clarified terminology and discovered that while most focus group members were not familiar with the terms, they were well acquainted with the relational concepts of networking and social benefits tied to expanding social relationships within groups and across groups and society.^[[4]](#endnote-4)^ In response to the question, “How has the FESEP program helped to create greater social capital for individuals and the organization (both linking and bonding capital)?” focus group members discussed the topic at length, noting they had created a lot of social capital in the program’s ten years of existence.

***Bonding social capital.*** Users indicated creation of social capital for them as individuals:

- “We socialize, our group is united, we get along well, we dance, we joke.”
- “The program helps us to be more friendly people and to be able to make friends.”
- “In the program group we have trust, respect, and people to hang with.”

There is also social capital for families at the group level:

- “We have a high level of togetherness, of trust among families.”
- “At an internal level, the program generates trust among everyone. Our group norms are borne from all the participants.”

***Bridging social capital.*** They stated the group creates social capital through linking to other organizations:

- “We have access to and response from national institutions, even up to the director of the national psychiatric hospital and the director of mental health for the Ministry of Health.”
- “We have contact with many groups now including university professors and their classes, the police, leaders in the Ministry of Health, user and family groups in other countries, and nonprofits serving other populations through our work on the national health forum and the national disability council.”^[[5]](#endnote-5)^

***More social capital.*** Finally, focus group members also noted the need for creating more social capital:

- “There is power in social movements such as ALJES, which has 10,000 members. But we don’t know how many people there are with mental illnesses in the country, how many members we could have.”
- “Development programs have traditionally focused on poverty, infant mortality, and so on. Why not suicide?”

1. In El Salvador, as in other Central American countries, this problem confronts the whole mental health services system, which is concentrated in a central psychiatric hospital in the country’s capital city. Rarely are there community services, or even decentralized psychiatric units in regional hospitals. For a family to obtain psychiatric services they might have to get a psychotic person on a public bus for 6 hours, or pay a private truck driver to transport the person. Far from home, there is no connection with family and no interaction between hospital staff and family caregivers. When the person is released there is no ongoing medication monitoring or adjustments. [↑](#endnote-ref-1)
2. As of 2015, this program ASFAE now provides services to consumers, and has adopted the FESEP structured educational program. Additionally, through a grant from the Inter-American Foundation, ACISAM is now training family and user groups in Nicaragua, Costa Rica and Panama in the same family education program. [↑](#endnote-ref-2)
3. In El Salvador these two sectors are very distinct and separate. The government rarely funds or contracts with nonprofits. Nonprofits instead depend on funding from international foundations or foreign governments. Many NGOs face an uphill battle to overcome the culturally ingrained tendencies toward obeisance to authority and dependence on the boss/owner, who typically is in direct control of all financial assets and human resources, and which is typical of governmental structures as well. [↑](#endnote-ref-3)
4. El Salvador is a very relational society: family is a big priority; work is often obtained only because you have a connection to the boss; and respect for authority is a guiding principle for accomplishing tasks. [↑](#endnote-ref-4)
5. The national health forum is known as el “Foro Nacional” (National Forum Against the Privatization of Health) and the national disability council as “CONAIPD” (National Council for Integral Attention to Persons with Disabilities). [↑](#endnote-ref-5)
